# ﻿Taxonomy and distribution of *Taraxacum* sect. *Erythrosperma* (Asteraceae) in Poland

**DOI:** 10.3897/phytokeys.224.99463

**Published:** 2023-04-05

**Authors:** Mateusz Wolanin, Ewelina Klichowska, Iwona Jedrzejczyk, Monika Rewers, Marcin Nobis

**Affiliations:** 1 Institute of Biology, University of Rzeszów, Zelwerowicza 4, 35–601, Rzeszów, Poland University of Rzeszów Rzeszów Poland; 2 Institute of Botany, Faculty of Biology, Jagiellonian University, Gronostajowa 3, 30–387, Kraków, Poland Jagiellonian University Krakow Poland; 3 Laboratory of Molecular Biology and Cytometry, Department of Agricultural Biotechnology, Bydgoszcz University of Science and Technology, Kaliskiego 7, 85–796, Bydgoszcz, Poland Bydgoszcz University of Science and Technology Bydgoszcz Poland

**Keywords:** Dandelions, distribution, ecological modelling, flow cytometry, genome size, identification key, morpholo­gy, nuclear DNA content, SCoT markers, taxonomy

## Abstract

The dandelions from Taraxacumsect.Erythrosperma are taxonomically well distinguished and ecologically restricted to warm and sunlit habitats of steppes, dry and sandy grasslands, and distributed in temperate regions of Europe and Central Asia, with some being introduced to North America. Despite the long tradition of botanical research, the taxonomy and distribution of dandelions of T.sect.Erythrosperma is still underexplored in central Europe. In this paper, by combining traditional taxonomic studies supported by micromorphological, molecular and flow cytometry analyses as well as potential distribution modelling we shed light on taxonomical and phylogenetical relationships between members of T.sect.Erythrosperma in Poland. We also provide an identification key, species-checklist, detailed descriptions of morphology and occupated habitats as well as distribution maps for 14 Polish erythrosperms (*T.bellicum*, *T.brachyglossum*, *T.cristatum*, *T.danubium*, *T.disseminatum*, *T.dissimile*, *T.lacistophyllum*, *T.parnassicum*, *T.plumbeum*, *T.proximum*, *T.sandomiriense*, *T.scanicum*, *T.tenuilobum*, *T.tortilobum*). Finally, conservation assessments performed using the IUCN method and threat categories for all the examined species are proposed.

## ﻿Introduction

Human activity, climate warming and biological invasions have recently become the main factors threatening natural biodiversity in many regions of the world (Robinson et al. 2020; Wallingford et al. 2020). Environmental changes, to cite some examples, the deforestation of tropical forests, drying of bogs and meadows and their conversion to arable fields as well as expansive urban development, have a strong negative impact on the diversity of many groups of organisms. Many species become extinct even before they are discovered and described (Costello et al. 2013; Lees and Pimm 2015). This phenomenon is clearly visible in tropical regions, and the most prominent example is orchids, one of the most diversified and still underexplored groups of species (Szlachetko and Kolanowska 2021; Kolanowska et al. 2022). However, the pro­blem also concerns species within taxonomically problematic and species-rich genera with morphologically similar or cryptic species, such as *Rubus*, *Hieracium*, *Campanula*, *Orobanche*, *Stipa*, *Oxytropis*, etc. (Wolanin et al. 2016, 2020; Aleksić et al. 2018; Piwowarczyk et al. 2019; Chen et al. 2020; Nobis et al. 2020a; Baiakhmetov et al. 2021; Kosiński et al. 2021; Trávníček et al. 2021; Havlíček et al. 2022; Szeląg 2022; Vintsek et al. 2022; Wang et al. 2022; Nobis et al. 2023). Globally distributed dandelions (*Taraxacum*, Asteraceae) can also be included in this group (Uhlemann 2002, 2016; Scott and Rich 2013; Štěpánek and Kirschner 2017; Marciniuk et al. 2018; Kirschner et al. 2020, 2022).

The genus *Taraxacum* Wigg. comprises ca. 3000 species classified into 60 sections distributed globally throughout the temperate zone (Vašut 2003; Reisch 2004; Kirschner et al. 2015; Vašut and Majeský 2015). Most of them are apomicts, sexually reproducing diploids being rare (Richards 1970; Mogie and Ford 1988; Van Dijk 2003). Diploids coexist with polyploidy apomicts in most sections. Only a few of them are considered primitive (T.sect.Piesis (DC.) Kirschner & Štěpánek, T.sect.Dioszegia (Heuffel) Heuffel, T.sect.Biennia R. Doll, T.sect.Glacialia Handel-Mazzetti, T.sect.Wendelboa (Soest) R. Doll), containing exclusively diploid species (Kirschner and Štěpánek 1996). The majority of the young European and Asian sections (e.g. T.sect.Taraxacum, T.sect.Palustria, T.sect.Erythrosperma) originate from hybrid taxa most likely formed during the Pleistocene glaciation as a result of multiple contacts of southern and northern species ranges (Kirschner and Štěpánek 1996; Marciniuk et al. 2010).

Taraxacumsect.Erythrosperma (Lindb.) Dahlst. consists of at least one sexual di­ploid species *T.erythrospermum* Andrz. ex Besser and approximately 150 apomictic polyploid species distributed in Europe, Anatolia, Central Asia (Kirschner and Štěpánek 1994; Wittzell 1999; Vašut 2003; Reisch 2004; Marciniuk et al. 2009, 2010; Mártonfiová et al. 2010; Štěpánek and Kirschner 2012; Wendt and Øllgaard 2015) and introduced to North America. In Poland, however, all confirmed species of erythrosperms were triploids (Wolanin and Musiał 2017, 2018; Wolanin et al. 2018), although some tetraploids were also reported in central Europe (Van Soest 1967). Species belonging to T.sect.Erythrosperma are usually small-sized, forming a tunic of dried leaf leftover, with deeply lobed leaves, narrow lobes and petioles, small outer bracts, and mostly red or straw-coloured, strongly spinulose achenes with long cylindrical cone. Dandelions belonging to this group are adapted to warm and sunlit habitats, such as sandy grasslands, pseudosteppes, steppes, xerothermic swards, and ruderal communities (Dudman and Richards 1997; Vašut 2003; Wendt and Øllgaard 2015).

In his revision of European erythrosperms, Doll (1973a, b) mentioned 115 species. Based on those works, in the first revision of Polish dandelions of T.sect.Erythrosperma, Tacik (1980) listed 18 species as present in Poland (Table 1) and 20 as possible to be found. Twenty years later, four species previously unknown from Poland, *T.disseminatum* G. E. Haglund, *T.bellicum* Sonck (= *T.prunicolor* Schmid, Vašut & Oosterv.), *T.maricum* Vašut, Kirschner & Štěpánek and *T.cristatum* Kirschner, Štěpánek & Vašut, were reported by Øllgaard et al. (2002a), Vašut et al. (2005) and Marciniuk et al. (2009). Subsequently, three species, *T.danubium* Richards, *T.tortilobum* Florstr., and *T.sandomiriense* Wolanin, were very recently recorded by Wolanin and Musiał (2018) and Wolanin et al. (2018). All species reported from Poland so far have been listed in the most recent checklist by Mirek et al. (2020).

**Table 1.** Taraxacumsect.Erythrosperma species reported from Poland. The 'Recent sources' are Øllgaard et al. (2000, 2002a, 2002b), Głowacki and Czarna (2003), Vašut et al. (2005), Marciniuk et al. (2009), Wolanin and Musiał (2018), Wolanin et al. (2018); ? – determination uncertain.

Species	Tacik 1980	Recent sources	Mirek et al. 2020
*T.bellicum*	·	+	+
*T.brachyglossum*	+	+	+
*T.brunneum*	+	·	+
*T.cristatum*	·	+	+
*T.danubium*	·	+	+
*T.discretum*	+	·	+
*T.disseminatum*	·	+	+
*T.dissimile*	+	+	+
*T.erythrospermum*	+	·	+
*T.falcatum*	+	·	+
*T.fulvum*	+	·	+
*T.gracillimum*	+	·	+
*T.lacistophyllum*	+	+	+
*T.laetum*	+	·	+
*T.leptocarpum*	+	·	+
*T.marginatum*	+	·	+
*T.maricum*	·	+?	+
*T.parnassicum*	+	+	+
*T.plumbeum*	+	+	+
*T.proximum*	+	+	+
*T.sandomiriense*	·	+	+
*T.scanicum*	+	+	+
*T.simile*	+	·	+
*T.tenuilobum*	+	+	+
*T.tortilobum*	·	+	+

Due to enormous species-richness in the genus *Taraxacum*, the presence of multiple hybridisation events, frequent polyploidy and apomictic reproduction, as well as the limited number of studies related to the diversity and distribution of its species (Kirschner and Štěpánek 1996; Marciniuk et al. 2010; Kirschner et al. 2016; Jafari et al. 2018; Lee et al. 2021), the biogeography, phylogeny and genetic diversity of dandelions unfortunately still remain poorly explored. Most of the research so far focused on establishing general intrageneric phylogenetic relationships by using representatives belonging to different sections (Van der Hulst et al. 2003; Kirschner et al. 2016) or between selected species occurring in a given area of interest (Majeský et al. 2012; Marciniuk et al. 2020; Lee et al. 2021). Some more detailed studies concerned population genetics (Jafari et al. 2018) or variation between complete chloroplast genomes (Salih et al. 2017; Lee et al. 2021) regarding selected individual species. Research on phylogenetic relationships between species within particular sections is also relatively sparse (Marciniuk et al. 2020). The studies of Reisch (2004) and Majeský et al. (2015) are, to the best of our knowledge, the only ones to concentrate exclusively on species from T.sect.Erythrosperma, and the authors used various analyses such as random amplified polymorphic DNA (RAPD), nuclear Simple Sequence Repeats (SSR), amplified fragment length polymorphism (AFLP) DNA markers and a selected sequenced region of chloroplast DNA to study phylogenetic relationships and genetic differentiation respectively between and within the examined taxa.

In terms of the evolution of the genus *Taraxacum*, other important aspects to consider are the level of ploidy and genome size (Záveský et al. 2005; Macháčková et al. 2018). In this respect, some taxa are better studied (e.g. sect. Taraxacum; Macháčková et al. 2018), whereas others, including T.sect.Erythrosperma, much less so. Although the karyology of Central European representatives of T.sect.Erythrosperma is well known (Gustafsson 1934; Małecka 1967, 1969; Doll 1973b; Dudman and Richards 1997; Uhlemann 2000; Schmid et al. 2004; Grzesiuk et al. 2008; Trávníček et al. 2010; Majeský et al. 2015; Wolanin and Musiał 2017, 2018; Wolanin et al. 2018), nuclear DNA content has been reported only for *T.brachyglossum* (Záveský et al. 2005).

It is assumed that evolutionary processes within different sections of *Taraxacum* are linked to the appearance of new habitats or habitat specialisation within a group of hybrids (Kirschner and Štěpánek 1996), and microspecies commonly show some different eco-geographical patterns (Trávníček et al. 2010; Macháčková et al. 2018). Sexually reproducing diploids and asexual triploids from the same sections have been proven to differ in terms of geographic ranges and occupied niches (Meirmans 2021). Triploid plants from T.sect.Erythrosperma are characterised by a wider geographic range and a much more extensive ecological niche compared to the diploids (Meirmans 2021), which may suggest noticeable differences between triploid taxa within the section. Due to the vegetation period suitable for proper collection of dandelions being restricted to early spring and some of the localities having been found accidentally during excursions, the distribution maps of *Taraxacum* species may be incomplete. Thus, species distribution modelling (SDM) can contribute important information for the studied dandelions.

In this paper, by combining traditional taxonomic studies supported by micromorphological, molecular and flow cytometry analyses as well as potential distribution modelling, we shed light on taxonomical relationships between members of T.sect.Erythrosperma in Poland. In particular we would like to answer the following questions: I) which species in T.sect.Erythrosperma occur in Poland; II) in which regions and types of habitat do the species occur; III) which morphological characters are species-specific and helpful in species identification; IV) could micromorphological characters of achenes be useful in species identification; V) does the molecular analysis confirm the distinctiveness of the taxa identified on the basis of morphological characters, and what are their phylogenetic relationships? This work also contains an easy-to-use identification key, morphological descriptions and photos of representative specimens that significantly facilitate their determination.

## ﻿Materials and methods

### ﻿Field studies, distribution and morphological analyses

Field studies were carried out in 2012–2019, from mid-April to mid-May, and supplemented in May 2021. Plants were initially determined in the field and collected from each population. Individuals that were causing problems with determination (juvenile plants or damaged plants from habitats under anthropopressure) were dug out, cultivated and observed for several seasons. The geographic coordinates of the localities were determined by GPS equipment. For a description of plant communities with a share of dandelions of the sect. Erythrosperma, floristic lists were prepared. The notes were complemented in mid-June, and the names of the species were given after Mirek et al. 2020. The herbarium collection is deposited in the Institute of Biology, University of Rzeszów (UR), with the exception of *T.sandomiriense* types, which were deposited earlier in the Herbarium of the Institute of Botany of the Jagiellonian University (KRA). The revision of plant collections was carried out in the following herbaria: KRA, KRAM, WRAB, KTU, UGDA, SZUB, POZNB, MPD and Herb. J&P Marciniuk. Maps of species distribution in Poland were prepared using the cartogram (ATPOL) method (Zając 1978) on a 10×10 km square grid. Morphological studies were conducted on both living and herbarium plants using a ruler and a stereo microscope equipped with an eyepiece reticle.

### ﻿Macromorphological analyses of achenes

Achenes for macromorphological studies were collected from mature plants, at least 40 achenes per 3–5 plants from each population (Table 2). Five morphological characters were studied: achene length (incl. cone), cone length, achene body width, length of achene body spinose part, and length of beak. Samples are deposited in the Institute of Biology, University of Rzeszów.

**Table 2.** List of populations of Taraxacumsect.Erythrosperma examined for macromorphology of the achenes.

Species	Locality	Geographical coordinates
*T.bellicum*	Kraków Kostrze	50°02'N, 19°52'E
	between Zaklików and Lipa	50°42'N, 22°04'E
	Klimaszewnica	53°28'N, 22°30'E
*T.brachyglossum*	Miasteczko Śląskie	50°29'N, 18°55'E
	Sosnowiec Maczki	50°15'N, 19°17'E
	Olsztyn	50°45'N, 19°16'E
*T.cristatum*	Grząby Bolmińskie	50°48'N, 20°21'E
	Przewodziszowice	50°38'N, 19°23'E
	Grząby Bolmińskie II	50°48'N, 20°21'E
*T.danubium*	Olsztyn	50°45'N, 19°16'E
	Olsztyn II	50°45'N, 19°16'E
	Pychowicka Górka	50°02'N, 19°53'E
*T.disseminatum*	Piątnica (Fort Łomża)	53°12'N, 22°07'E
	Chwałkowo Kościelne	51°59'N, 17°18'E
*T.dissimile*	Osowiec	53°29'N, 22°38'E
	between Krynica Morska and Piaski	54°24'N, 19°31'E
	Hel Leśna Street	54°36'N, 18°49'E
*T.lacistophyllum*	Gdańsk Stogi	54°22'N, 18°34'E
	Gdańsk (Roland pleasure ground)	54°24'N, 18°36'E
	Łeba	54°45'N, 17°32'E
*T.parnassicum*	Kusięta	50°46'N, 19°16'E
	Miedzianka	50°50'N, 20°21'E
	Sąspów	50°13'N, 19°46'E
*T.plumbeum*	between Zaklików and Lipa	50°42'N, 22°04'E
	Stare Bielice	52°51'N, 15°55'E
	Kraków Kostrze	50°02'N, 19°52'E
*T.proximum*	Krynica Morska	54°23'N, 19°28'E
	Piątnica (Fort Łomża)	53°11'N, 22°07'E
	Chwałkowo Kościelne	51°59'N, 17°18'E
*T.sandomiriense*	Kamień Łukawski	50°41'N, 21°47'E
*T.scanicum*	Zbrzeźnica	53°02'N, 22°10'E
	Gdańsk Stogi	54°22'N, 18°43'E
	Ługi	51°59'N, 17°11'E
*T.tenuilobum*	Kroczyce	50°34'N, 19°31'E
	Miedzianka	50°50'N, 20°21'E
	Łeba	54°46'N, 17°34'E
*T.tortilobum*	Gdańsk Stogi	54°22'N, 18°43'E
	Gdańsk Stogi II	54°22'N, 18°43'E
	Gdańsk Stogi III	54°22'N, 18°43'E

### ﻿Micromorphological analyses of achenes (SEM observations)

For the SEM observations, the achenes were attached to aluminium stubs using Pelco conductive liquid silver and sputtered with 20 nm of gold using a turbo-pumped Quorum Q 150T ES coater. Samples (Table 3) were observed using a scanning electron microscope (Hitachi High-Technologies Corporation, Tokyo, Japan) operated at 5 kV and 10 mm distance.

**Table 3.** Samples used in SEM observations of the achenes.

Species	Locality	Geographical coordinates
*T.bellicum*	between Zaklików and Lipa	50°42'N, 22°04'E
*T.brachyglossum*	Kusięta	50°46'N, 19°16'E
*T.cristatum*	Przewodziszowice	50°38'N, 19°23'E
*T.danubium*	Olsztyn	50°45'N, 19°16'E
*T.disseminatum*	Chwałkowo Kościelne	51°59'N, 17°18'E
*T.dissimile*	Łeba	54°46'N, 17°34'E
*T.lacistophyllum*	Grańsk Stogi	54°22'N, 18°34'E
*T.parnassicum*	Sąspów	50°13'N, 19°46'E
*T.plumbeum*	between Zaklików and Lipa	50°42'N, 22°04'E
*T.proximum*	Gdańsk Stogi	54°22'N, 18°43'E
*T.sandomiriense*	Kamień Łukawski	50°41'N, 21°47'E
*T.scanicum*	Łeba	54°46'N, 17°35'E
*T.tenuilobum*	Łeba	54°46'N, 17°34'E
*T.tortilobum*	Gdańsk Stogi	54°22'N, 18°43'E

Micromorphological structures of achenes were observed and photographs taken by means of the scanning electron microscope Hitachi SU 8010 at various magnifications (Figs 5–7). Achenes were studied from base to distal portions. The following qualitative characters were studied: the shape and arrangement of achene spines; details of surface ornamentation of the achene body, achene spines, the upper part of the achene body and the middle part of the cone. Samples are deposited in the Institute of Biology, University of Rzeszów.

### ﻿DNA extraction

Isolation of genomic DNA was performed from dried leaf tissues, which were ground to a fine powder using a mixer mill MM400 (Retsch) and 3–5 mm glass beads. Isolation of genomic DNA was performed using a modified CTAB method (Doyle and Doyle 1987). The isolated DNA was purified using a gDNA Clean kit (Syngen, Poland) when necessary. The purity and concentration of extracted DNA were evaluated using a NanoDrop ND-1000 spectrophotometer (Thermo Fisher Scientific, USA). The quality of extracted DNA was roughly verified by electrophoresis on 1% agarose gels. The total number of samples used for further molecular analysis was 34. We decided to use *T.jugiferum* H. Øllg. and *T.stridulum* Trávn. ined as outgroups. The latter species is easy to distinguish although still not described (*nomen provisorium*; Trávníček pers. comm.) (Table 4).

**Table 4.** List of samples used in molecular analysis.

Sample ID	Species	Section	Locality	Geographical coordinates
T56	*T.bellicum*	Erythrosperma	Siematycze	52°24'N, 22°56'E
T57	*T.bellicum*	Erythrosperma	Klimaszewnica II	53°28'N, 22°30'E
T58	*T.bellicum*	Erythrosperma	Klimaszewnica	53°28'N, 22°30'E
T59	*T.bellicum*	Erythrosperma	Arbasy	52°31'N, 22°32'E
T2	*T.brachyglossum*	Erythrosperma	Kusięta	50°46'N, 19°16'E
T31	*T.brachyglossum*	Erythrosperma	Kusięta II	50°46'N, 19°16'E
T13	*T.cristatum*	Erythrosperma	Grząby Bolmińskie	50°48'N, 20°21'E
T28	*T.danubium*	Erythrosperma	Olsztyn	50°45'N, 19°16'E
T51	*T.danubium*	Erythrosperma	Góra Sfinks	50°44'N, 19°16'E
T52	*T.danubium*	Erythrosperma	Kraków Kostrze	50°02'N, 19°52'E
T10	*T.disseminatum*	Erythrosperma	Chwałkowo Kościelne	51°59'N, 17°18'E
T5	*T.dissimile*	Erythrosperma	Osowiec	53°29'N, 22°38'E
T11	*T.lacistophyllum*	Erythrosperma	Gdańsk Stogi	54°22'N, 18°34'E
T27	*T.parnassicum*	Erythrosperma	Miedzianka	50°50'N, 20°21'E
T30	*T.parnassicum*	Erythrosperma	Kusięta	50°46'N, 19°16'E
T9	*T.parnassicum*	Erythrosperma	Kusięta II	50°46'N, 19°16'E
T14	*T.plumbeum*	Erythrosperma	Dźwirzyno	54°10'N, 15°26'E
T48	*T.plumbeum*	Erythrosperma	between Kębłowo and Świętno	52°03'N, 16°05'E
T49	*T.plumbeum*	Erythrosperma	Sąsieczno	52°57'N, 18°51'E
T50	*T.plumbeum*	Erythrosperma	near Golub-Dobrzyń	53°04'N, 18°58'E
T38	*T.cf.plumbeum*	Erythrosperma	Wola Mała	50°33'N, 22°46'E
T33	*T.sandomiriense*	Erythrosperma	Kamień Łukawski	50°41'N, 21°47'E
T36	*T.scanicum*	Erythrosperma	Łysaków Kolonia	50°45'N, 22°07'E
T44	*T.scanicum*	Erythrosperma	Piła	53°09'N, 16°47'E
T45	*T.scanicum*	Erythrosperma	Młodzieszyn	52°19'N, 20°12'E
T46	*T.scanicum*	Erythrosperma	Sowia Góra	52°42'N, 15°51'E
T6	*T.scanicum*	Erythrosperma	Zbrzeźnica	53°02'N, 22°10'E
T4	*T.tenuilobum*	Erythrosperma	Miedzianka	50°50'N, 20°21'E
T53	*T.tortilobum*	Erythrosperma	Gdańsk Stogi	54°22'N, 18°43'E
T54	*T.tortilobum*	Erythrosperma	Gdańsk Stogi II	54°22'N, 18°43'E
T55	*T.tortilobum*	Erythrosperma	Gdańsk (Roland pleasure ground)	54°25'N, 18°36'E
T21	*T.jugiferum*	Taraxacum	Błażowa	49°53'N, 22°06'E
T20	*T.stridulum*	Taraxacum	Błażowa	49°53'N, 22°06'E

### SCoT-PCR amplifications

Start Codon Targeted (SCoT) polymorphism is a newly emerged DNA molecular marker developed based on the targeting start codon of the genes and their surrounding consensus sequences in a gene family (Collard and Mackill 2009). Due to their simplicity, relatively low cost requirements, high reproducibility, and considerable association with phenotypic data, SCoT markers has been applied to many genetic studies, including analysis of genetic diversity, detecting intra- and inter-genetic variation in different plant species, stability of *in vitro* derived plants, phylogenetic relationships, taxonomy, species/cultivar identification, quantitative trait loci (QTL) mapping and DNA fingerprinting in various plants (e.g. Zhang et al. 2015; Etminan et al. 2018; Jalilian et al. 2018; Jedrzejczyk 2020; Zarei and Erfani-Moghadam 2021; Rai 2023).

Of a set of 20 tested SCoT primers (Genomed, Poland), 19 generated stable band patterns were selected for further studies (Table 5). All the PCR reactions were carried out within a total volume of 12.5 µl, containing 30 ng of genomic DNA template, 0.1 U/µl Taq DNA polymerase, 4 mM MgCl_2_ and 0.5 mM of each dNTPs (2xPCR Master Mix Plus; A&A Biotechnology, Poland), 10 µM of primer and sterile deionised water to the final volume. The DNA amplifications were performed using T100 Thermal Cycler (BioRad, USA) under the following conditions: initial denaturation at 94 °C for 5 min., followed by 35 cycles of 94 °C for 1 min., annealing for 1 min., and extension at 72 °C for 2 min. The last cycle was followed by the final extension step of 7 min. at 72 °C. The annealing temperature for each primer was optimised, and varied from 49.5 °C to 63.5 °C (Table 5). Amplified PCR products were separated by electrophoresis using 1.5% (w/v) agarose gel made in 1.0× TBE buffer and stained with ethidium bromide (0.5 µg/mL). A DNA ladder of 3000 bp (Thermo Fisher Scientific, USA) was used to determine the size of the amplicons. The gels were visualised under UV light and photographed using GelDoc XR+ (BioRad, USA).

**Table 5.** SCoT primers used in the molecular description of *Taraxacum* samples.

Primer code	Primer sequence (5'-3')	Annealing temperature (°C)	No. of total loci	No. of polymorphic loci	Percentage of polymorphism	PIC
SCoT-2	CAACAATGGCTACCACCC	51.0	23	23	100	0.42
SCoT-4	CAACAATGGCTACCACCT	49.5	14	13	93	0.50
SCoT-5	CAACAATGGCTACCACGA	50.0	16	16	100	0.47
SCoT-6	CAACAATGGCTACCACGC	51.0	19	19	100	0.49
SCoT-7	CAACAATGGCTACCACGG	51.0	10	9	90	0.49
SCoT-9	CAACAATGGCTACCACGT	50.0	18	17	94	0.37
SCoT-11	AAGCAATGGCTACCACCA	50.0	15	13	87	0.49
SCoT-12	ACGACATGGCGACCAACG	56.0	17	15	88	0.46
SCoT-14	AGGACATGGCGACCACGC	56.0	15	14	93	0.46
SCoT-17	ACCATGGCTACCACCGAG	54.0	17	16	94	0.33
SCoT-21	CACCATGGCTACCACCAT	51.0	14	13	93	0.46
SCoT-25	ACCATGGCTACCACCGGG	56.0	13	12	92	0.50
SCoT-26	ACCATGGCTACCACCGTC	54.0	11	10	91	0.42
SCoT-27	ACCATGGCTACCACCGTG	54.0	22	22	100	0.50
SCoT-32	CCATGGCTACCACCGCAC	56.0	18	18	100	0.46
SCoT-33	CCATGGCTACCACCGCAG	56.0	26	23	88	0.44
SCoT-34	ACCATGGCTACCACCGCA	54.0	19	18	95	0.48
SCoT-35	CATGGCTACCACCCGCCC	63.5	17	17	100	0.46
SCoT-36	GCAACAATGGCTACCACC	51.0	15	13	87	0.42
**Average**			**18**	**16**	**94**	**0.45**

### ﻿Data analysis of SCoT-PCR products

The PCR-amplified SCoT products were detected on gels and scored as a binary data matrix, as the presence (1) or absence (0) of a band. Only clear, reproducible and well-defined bands were counted. The numbers of monomorphic and polymorphic amplification products generated by each SCoT primer were determined. Polymorphic information content (PIC) was calculated according to Ghislain et al. (1999) by the formula: PIC = 1 – p2 – q2, where p is band frequency, and q is no band frequency. Genetic distances were calculated for all *Taraxacum* accessions, according to Nei and Li (1979), followed by a dendrogram construction using the unweighted pair group method, with arithmetic average (UPGMA), using the Treecon ver. 3.1 software (Van de Peer and De Wachter 1994). Statistical support of the branches was tested with bootstrap analysis using 2000 replicates.

### ﻿2C DNA content measurements

Genome size estimation was prepared according to the procedure of Jedrzejczyk and Sliwinska (2010) with minor modifications. The leaves of *Viciavillosa* 'Minikowska' (2C = 3.32 pg; Dzialuk et al. 2007) were used as an internal standard. Young and fresh leaves of 11 *Taraxacum* species (Table 6) and the internal standard were chopped simultaneously with a sharp razor blade in a plastic Petri dish with 1 ml of Galbraith's nucleus-isolation buffer (Galbraith et al. 1983) supplemented with an antioxidant of 1% (w/v) polyvinylpyrrolidone (PVP-10), propidium iodide (PI, 50 μg/mL) and ribonuclease A (50 μg/mL). The nuclei suspension was passed through a 50-μm mesh nylon and for each sample, 5000–7000 nuclei were measured using a CyFlow Ploidy Analyser (Sysmex Partec GmbH, Görlitz, Germany) equipped with a high-grade solid state laser with green light emission at 532 nm as well as with side (SSC) and forward (FSC) scatters. Histograms were evaluated using the CyFlow Cube software (Sysmex Partec GmbH, Görlitz, Germany). Genome size was estimated using the linear relationship between the ratio of *Taraxacum* and the internal standard 2C peak positions on the histogram. At least three replicates were analysed for each *Taraxacum* species. Mean coefficients of variation of the 2C DNA content were estimated for all samples and ranged from 5.00 to 6.20 (Table 7). The 2C DNA content (pg) was transformed to megabase pairs (Mbp) of nucleotides using the following conversion: 1 pg = 978 Mbp (Doležel and Bartoš 2005). The results were estimated using a one-way analysis of variance followed by Duncan's test (P < 0.05; Statistica v. 13, StatSoft, Poland).

**Table 6.** Samples used in genome size analysis.

Sample ID	Species	Locality	Geographical coordinates
43	*T.lacistophyllum*	Łeba	54°46'N, 17°32'E
9	*T.sandomiriense*	Kamień Łukawski	50°41'N, 21°47'E
10	*T.danubium*	Olsztyn	50°45'N, 19°16'E
12	*T.plumbeum*	Kraków Kostrze	50°02'N, 19°52'E
39	*T.plumbeum*	between Zaklików and Lipa	50°42'N, 22°04'E
13	*T.bellicum*	Kraków Kostrze	50°02'N, 19°52'E
14	*T.bellicum*	between Goniądz and Szafranki	53°29'N, 22°42'E
40	*T.bellicum*	between Zaklików and Lipa	50°42'N, 22°04'E
17	*T.cristatum*	Przewodziszowice	50°38'N, 19°23'E
19	*T.brachyglossum*	Miasteczko Śląskie	50°29'N, 18°55'E
20	*T.brachyglossum*	Kusięta	50°46'N, 19°16'E
38	*T.scanicum*	Łysaków Kolonia	50°45'N, 22°07'E
42	*T.scanicum*	Łeba	54°46'N 17°33'E
27	*T.tenuilobum*	Miedzianka	50°50'N, 20°21'E
28	*T.tenuilobum*	Bużka	52°21'N 22°54'E
41	*T.tenuilobum*	Łeba	54°46'N, 17°34'E
30	*T.dissimile*	Łeba	54°46'N, 17°34'E
33	*T.dissimile*	Hel Leśna Street	54°36'N, 18°49'E
34	*T.parnassicum*	Kusięta	50°46'N, 19°16'E
36	*T.parnassicum*	Miedzianka	50°50'N, 20°21'E
37	*T.parnassicum*	Sąspów	50°13'N, 19°46'E

**Table 7.** Genome size of *Taraxacum* species.

Species	DNA content			CV sample
	(pg/2C)±SD		Mbp/2C	
*T.bellicum*	2.33±0.014	de	2,279	5.61
*T.brachyglossum*	2.35±0.035	cd	2,298	5.77
*T.cristatum*	2.29±0.010	e	2,240	6.12
*T.danubium*	2.29±0.012	e	2,240	5.75
*T.dissimile*	2.37±0.019	cd	2,318	5.99
*T.lacistophyllum*	2.76±0.017	a	2,699	5.00
*T.parnassicum*	2.63±0.034	b	2,572	5.48
*T.plumbeum*	2.36±0.008	cd	2,308	5.75
*T.sandomiriense*	2.31±0.012	e	2,259	6.20
*T.scanicum*	2.36±0.022	cd	2,308	5.88
*T.tenuilobum*	2.38±0.019	c	2,328	6.04

### ﻿Distribution data

Distribution data of species from the Taraxacumsect.Erythrosperma were obtained from herbarium collections, published taxonomic studies (Uhlemann 2003; Vašut 2003; Schmid et al. 2004; Vašut et al. 2005; Dudáš 2014, 2018; Zámečník 2016; Dudáš et al. 2020; Nobis et al. 2020b; Dudáš and Vašut 2022), and our herbarium materials collected in the field. Since the studied species are a group of critical, morphologically similar taxa, we decided not to use data from available online databases. We determined the geographical coordinates of records from herbaria and the literature that had only locality descriptions and entered all coordinates into the WGS84 coordinate system. To avoid statistical artefacts related to pseudo-replications, only one datum of the species was considered for each 1 km cell of the grid (in correspondence with the resolutions of environmental layers used in our study). As a result, we used 633 localities for 11 species, including 113 localities for *T.bellicum*, 33 – *T.brachyglossum*, 32 – *T.cristatum*, 71 – *T.danubium*, 20 – *T.disseminatum*, 32 – *T.lacistophyllum*, 145 – *T.parnassicum*, 52 – *T.plumbeum*, 41 – *T.proximum*, 67 – *T.scanicum*, 27 – *T.tenuilobum*. Many species belonging to the studied complex are rare, known only from single localities; however, to meet the assumptions of ecological niche modelling, we chose only those known from at least 10 localities.

### ﻿Environmental data

In our studies, we used 19 bioclimatic variables, including Annual Mean Temperature (bio1), Mean Diurnal Range (bio2), Isothermality (bio3), Temperature Seasona­lity (bio4), Max Temperature of Warmest Month (bio5), Min Temperature of Coldest Month (bio6), Temperature Annual Range (bio7), Mean Temperature of Wettest Quarter (bio8), Mean Temperature of Driest Quarter (bio9), Mean Temperature of Warmest Quarter (bio10), Mean Temperature of Coldest Quarter (bio11), Annual Precipitation (bio12), Precipitation of Wettest Month (bio13), Precipitation of Driest Month (bio14), Precipitation Seasonality (bio15), Precipitation of Wettest Quarter (bio16), Precipitation of Driest Quarter (bio17), Precipitation of Warmest Quarter (bio18), Precipitation of Coldest Quarter (bio19), derived from the WorldClim 1.4 database (Hijmans et al. 2005; available online: http://www.worldclim.org/). Soil variables, including bulk density in tonnes per cubic-meter (bld), weight percentage of clay particles (< 0.0002 mm; cly), weight percentage of silt particles (0.0002–0.05 mm; slt), weight percentage of sand particles (0.05–2 mm; snd), soil organic carbon content in permilles (orc), volume percentage of coarse fragments (> 2 mm; crf), cation exchange capacity in cmol+/kg (cec), soil water-holding capacities (AWCtS), and pH (soil pH × 10 in H2O), were derived from the ISRIC database (Hengl et al. 2017) (https://www.isric.org/). We used layers at a spatial resolution of 30 arcseconds for both bioclimatic and soil variables.

### ﻿Ecological niche modelling

To model the potential distribution of species from Taraxacumsect.Erythrosperma, we used MaxEnt software version 3.3.3 k., a generative species distribution modelling tool recommended for applications involving presence-only datasets (Phillips et al. 2006; Phillips and Dudík 2008). We ran the model with default values (a maximum of 500 iterations, convergence threshold 0.00001, and five auto feature classes). We opted for a logistic format, as it is currently considered easier and potentially more accurate for interpretation than cumulative and raw approaches (Phillips and Dudík 2008; Baldwin 2009). Because some of the occurrence data were determined on the basis of descriptions of locations and maps, not coordinates obtained in the field, we decided to use 10 percentile training presence as a threshold rule, which eliminated the most outlying data. The model was calibrated using 75% of the occurrence records and tested on the remaining 25%. We performed 20 replicates using the subsample replicated run type and then averaged the results. To provide a different random test/train partition in each replicate, we used the 'random seed' option. We evaluated the final model using the threshold-independent area under the curve (AUC) generated automatically by MaxEnt (Phillips et al. 2006).

To select a set of variables appropriate for all species from the studied section, the initial model was run using all the above-mentioned variables as well as all 711 localities from all species. To avoid overfitting the model, we built a correlation matrix (Pearson's correlation coefficient) and removed highly correlated variables (r > 0.7). To choose which of the strongly correlated variables to remove, we performed a jackknife test of variable importance and eliminated variables that showed low or negative gain values (Baldwin 2009). However, when selecting the variables, we also took into account whether the variable could be easily explained from a biological point of view. Finally, we ran 17 models, separately for each species. To make the niches of individual species comparable, all models were run on the same set of 11 variables, six bioclimatic (bio3, bio7, bio10, bio11, bio18, bio19), and five physical and chemical properties of soil (awcts, cly, crf, orc, pH) (for abbreviation see Environmental data chapter). To make the models easier to interpret, we divided the probability of occurrence into 5 categories: very low (<0.2), low (0.2–0.4), medium (0.4–0.6), high (0.6–0.8), very high (>0.8), by using ArcMap 10.5 software (ESRI Inc 2016).

## ﻿Results and discussion

### SCoT markers analysis

**Figure 1.**
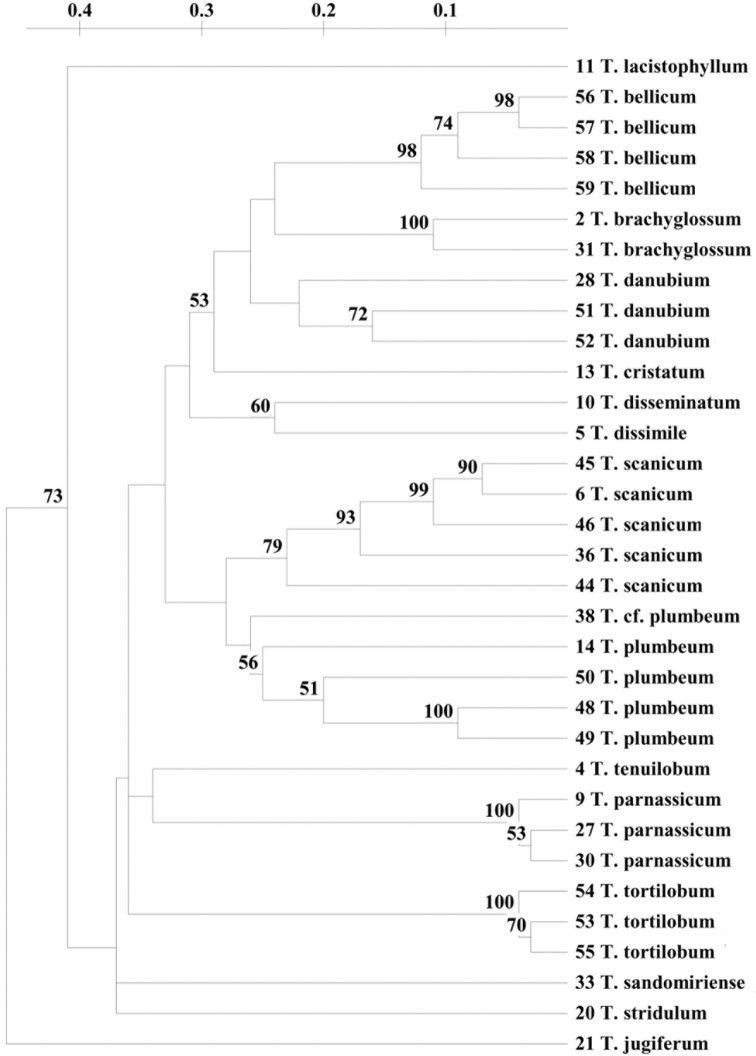
UPGMA dendrogram computed using a genetic distance matrix based on SCoT markers. Only bootstrap values > 50% are indicated. Scales demonstrate genetic distances.

We performed Internal transcribed spacer (ITS) analysis in the initial phase of the studies. The total alignment across the 32 individuals sampled was 680 bp. Although the alignment revealed differences in sequence length between the samples of dandelions, the tree topologies from the Bayesian inference method contained many polytomies, and were inconsistent with the morphological variation of studied species. Thus, we decided to use highly variable SCoT markers to differentiate species and establish the taxonomic relationships within section Erythrosperma. In total, 34 *Taraxacum* samples were analysed using 19 SCoT primers, which revealed reproducible band patterns. The primers amplified 319 loci, with 301 polymorphic bands. The number of bands generated per primer varied from 10 (SCoT-7) to 26 (SCoT-33). The size of the amplified bands ranged between 170 and 3000 bp. The percentage of polymorphism ranged from 87 to 100%, with an average of 94%. The PIC value, which describes the informativeness of the primer, varied from 0.33 (SCoT-17) to 0.50 (SCoT-4, SCoT-25 and SCoT-27), with an average of 0.45 (Table 5). The genetic distances estimated between 34 accessions of *Taraxacum* ranged from 0.03 to 0.56 (Suppl. material 1). In contrast to ITS, the UPGMA analysis based on SCoT markers revealed that samples belonging to the same taxon were grouped together, within the same cluster, thus confirming their proper taxonomic identification. However, the ordination of clusters in the UPGMA dendrogram is partially in polytomy. The largest clade with the two sister subclades comprise eight species in total: *T.bellicum*, *T.brachyglossum*, *T.danubium*, *T.cristatum*, *T.disseminatum* and *T.dissimile* in the first and *T.scanicum* and *T.plumbeum* in the second (Fig. 1). It is in polytomy with the subsequent two clusters, which comprise specimens belonging to *T.tenuilobum*, *T.parnassicum* and *T.tortilobum*. The remaining species, i.e., *T.sandomiriense*, *T.lacistophyllum* as well as *T.stridulum* and *T.jugiferum* form the outermost branches of the three. Although the last two mentioned species, as representatives of the section Taraxacum, represent an outgroup, based on SCoT analysis, *T.stridulum* was located closer to *T.sandomiriense* than to *T.jugiferum*. Whereas *T.jugiferum* was the most distant and not clustered with any of the examined species (Fig. 1). Similar results in terms of clustering of particular samples were presented by Reisch (2004). The author studied six species of erythrosperms, of which five also occurred in Poland. However, compared to our results, the relation of species segregated into particular clusters was somewhat different.

### ﻿Genome size of the examined *Taraxacum* species

The 2C DNA content of the 11 studied species ranged from 2.29 pg in *T.cristatum* and *T.danubium* to 2.76 pg in *T.lacistophyllum*, which corresponds to 2,240 and 2,699 Mbp, respectively (Table 7). All studied species possessed a very small genome size (Soltis et al. 2003), however, this is in line with the genomic size of other triploid representatives of the genus *Taraxacum* studied so far (Záveský et al. 2005; Macháčková et al. 2018). Statistical differences in genome size between species were detected, and two species (*T.lacistophyllum* and *T.parnassicum*) could be distinguished based on 2C DNA content. Within the species examined to date, similar minor variations or even no significant differences in genome size were observed, which may be explained by their asexual reproduction mode (Záveský et al. 2005; Macháčková et al. 2018). The differences in genome size (2.35 pg/2C vs. 2.62 pg/2C) in *T.brachyglossum* between our studies and the previous ones (Záveský et al. 2005) may result from both natural variance in DNA content as well as differences in the measurement methodology (Macháčková et al. 2018). To the best of our knowledge, the presented results provided new data on the genome size for 10 *Taraxacum* species.

### ﻿Macro- and micromorphology of achenes

**Figure 2.**
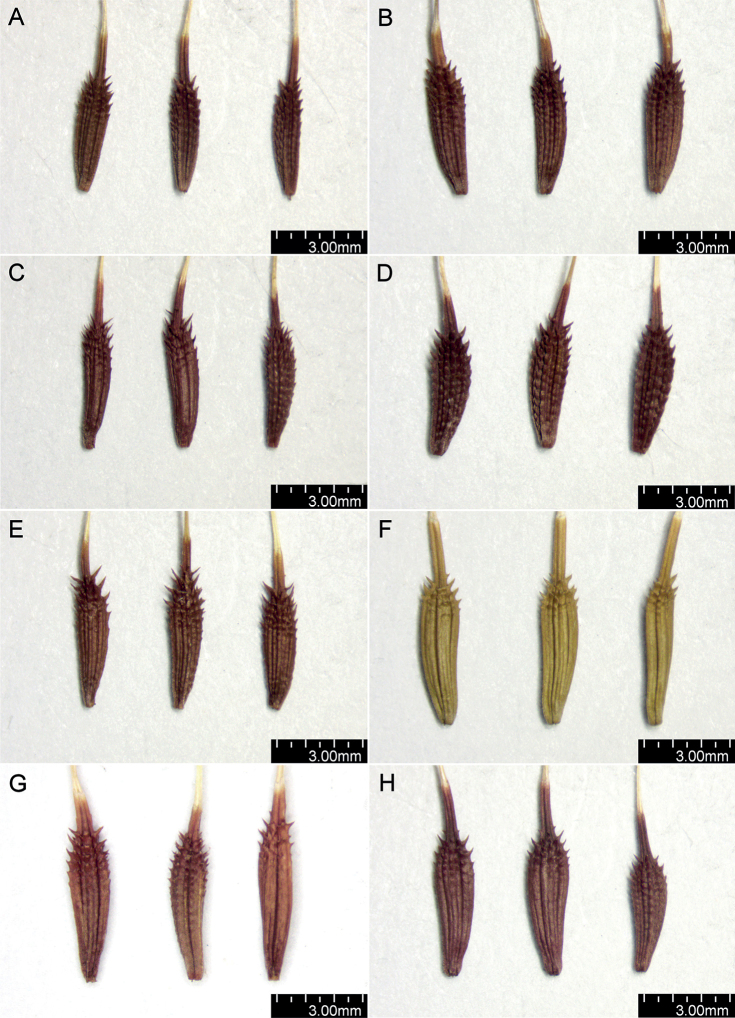
Shape and colour of achenes **A***T.bellicum***B***T.brachyglossum***C***T.cristatum***D***T.danubium***E***T.disseminatum***F***T.dissimile***G***T.lacistophyllum***H***T.parnassicum*.

**Figure 3.**
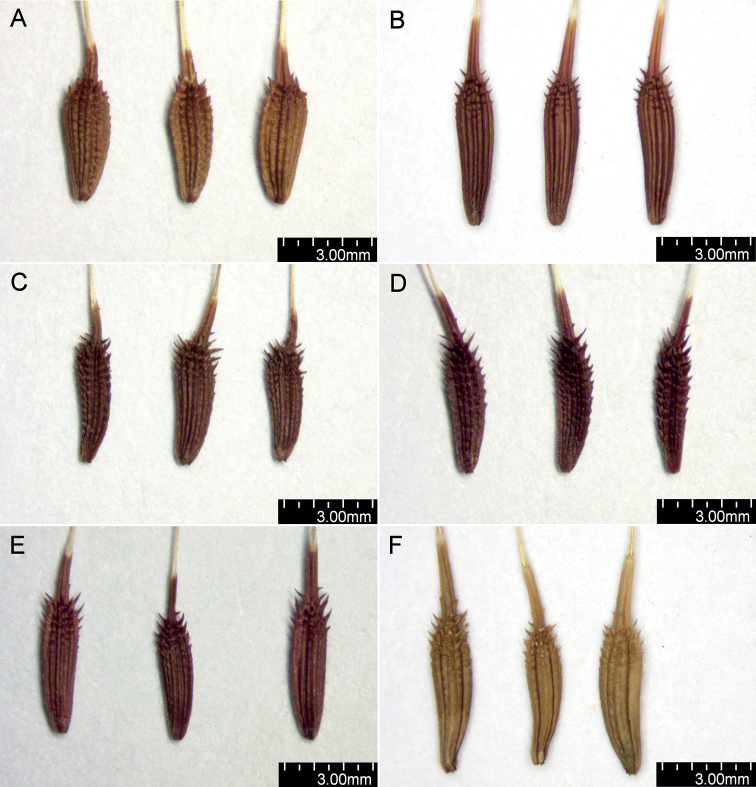
Shape and colour of achenes **A***T.plumbeum***B***T.proximum***C***T.sandomiriense***D***T.scanicum***E***T.tenuilobum***F***T.tortilobum*.

**Figure 4.**
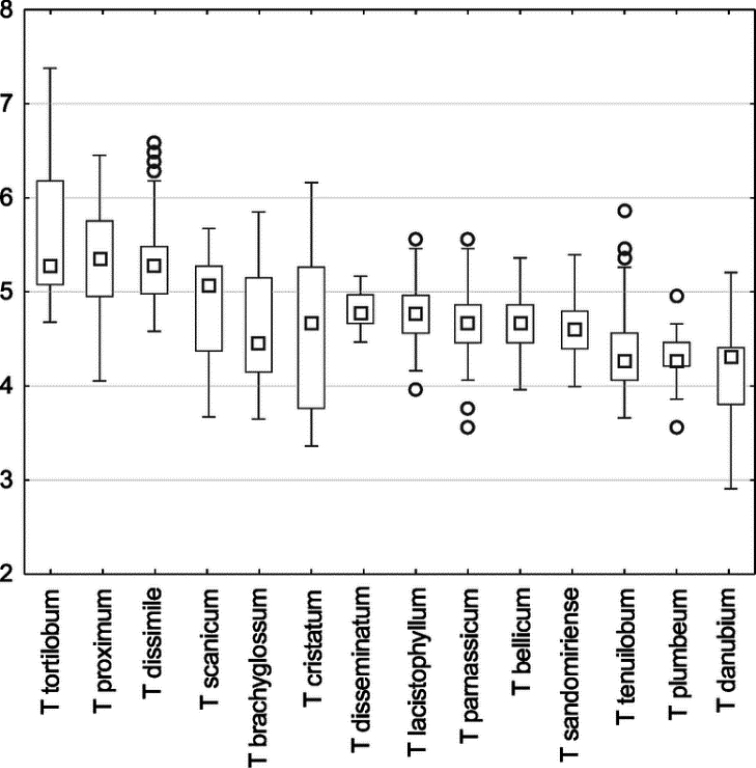
Box-and-whisker plots of achene length (incl. cone) in examined species. White squares indicate mean value (□), boxes represent the 25^th^ and 75^th^ percentile.

**Figure 5.**
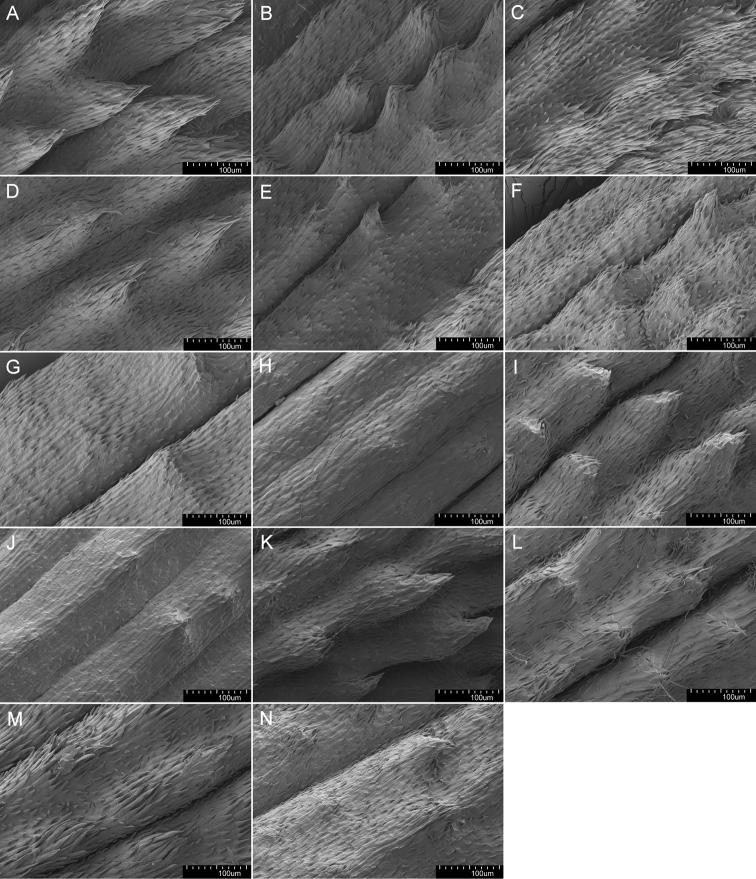
Micromorphology of achene spines of **A***T.bellicum***B***T.brachyglossum***C***T.cristatum***D***T.danubium***E***T.disseminatum***F***T.dissimile***G***T.lacistophyllum***H***T.parnassicum***I***T.plumbeum***J***T.proximum***K***T.sandomiriense***L***T.scanicum***M***T.tenuilobum***N***T.tortilobum* [magnification 300×].

**Figure 6.**
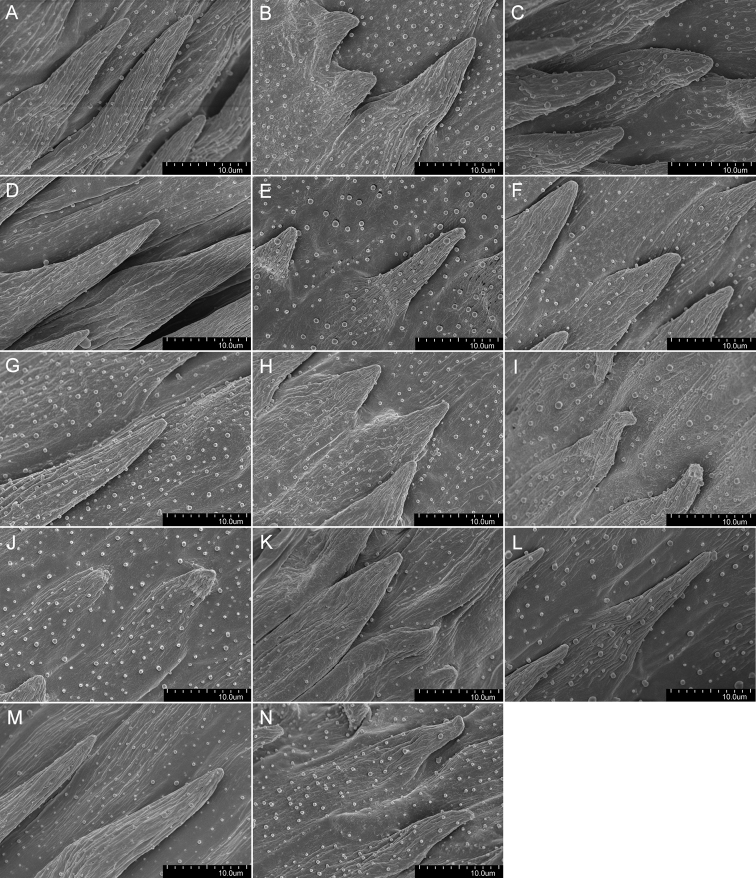
Micromorphology of the upper part of the achene body of **A***T.bellicum***B***T.brachyglossum***C***T.cristatum***D***T.danubium***E***T.disseminatum***F***T.dissimile***G***T.lacistophyllum***H***T.parnassicum***I***T.plumbeum***J***T.proximum***K***T.sandomiriense***L***T.scanicum***M***T.tenuilobum***N***T.tortilobum* [magnification 4000×].

**Figure 7.**
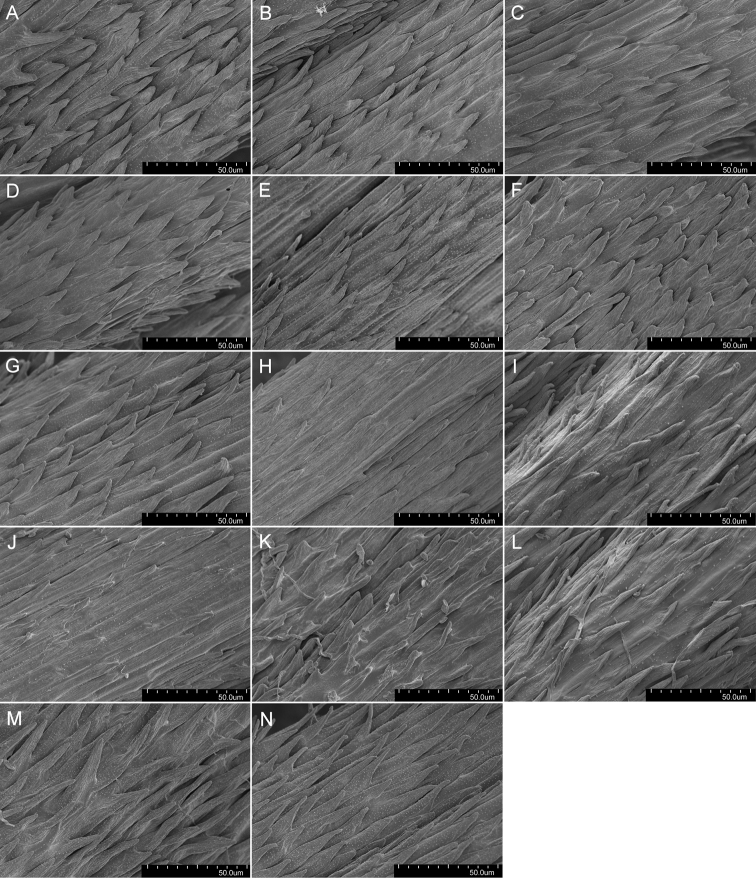
Micromorphology of the middle part of the cone of **A***T.bellicum***B***T.brachyglossum***C***T.cristatum***D***T.danubium***E***T.disseminatum***F***T.dissimile***G***T.lacistophyllum***H***T.parnassicum***I***T.plumbeum***J***T.proximum***K***T.sandomiriense***L***T.scanicum***M***T.tenuilobum***N***T.tortilobum* [magnification 1000×].

The shape and colour of achenes are important morphological features that greatly facilitate the identification of species representing the section Erythrosperma (Tacik 1980; Vašut 2003; Savadkoohi et al. 2012; Rewicz et al. 2020), (Figs 2, 3). During field work, we observed that, depending on the habitat conditions in which particular dandelions grow, the size of their achenes varies considerably, e.g. in the population of *T.lacistophyllum* from Roland pleasure ground in Gdańsk, the achenes of individuals growing in shadow (under the canopy of trees) were almost twice as long as compared to specimens growing in extremely dry conditions a few meters away. Preliminary analysis of five measurable achene features in *Erythrosperma* species showed very high similarity and a similar range of variability. All the examined taxa have rather similar achenes in terms of cone length, achene body width, length of the spinose part of the achene body, and beak length. Achene length (incl. cone) is one of the most species-specific morphological characters. Three species, *T.tortilobum*, *T.proximum* and *T.dissimile*, have the longest achenes as well as the longest cone, whereas the achenes of *T.tenuilobum*, *T.plumbeum* and *T.danubium* are the shortest (Fig. 4). However, there is also a group of the three species, *T.scanicum*, *T.brachyglossum* and *T.cristatum*, in which the length of achenes varies considerably. SEMs observations of achenes showed some morphological differences in the achenes' ornamentation (Figs 5–7), and in particular the spines' shape and extent of their fusion with the pericarp surface. For example in *T.parnassicum* and *T.proximum* (Figs 5–7H, J) the spines protrude only at the ends, while in *T.tenuilobum* the spines are slender and not fused in almost their entire length (Figs 5–7M). Such comparison may be helpful in the determination of juvenile specimens of some taxa, e.g. *T.scanicum* and *T.tenuilobum* (Figs 5–7L, M). The middle part of the cone seems to be a good area for such comparisons.

### ﻿Distribution of Taraxacumsect.Erythrosperma in Poland

**Figure 8.**
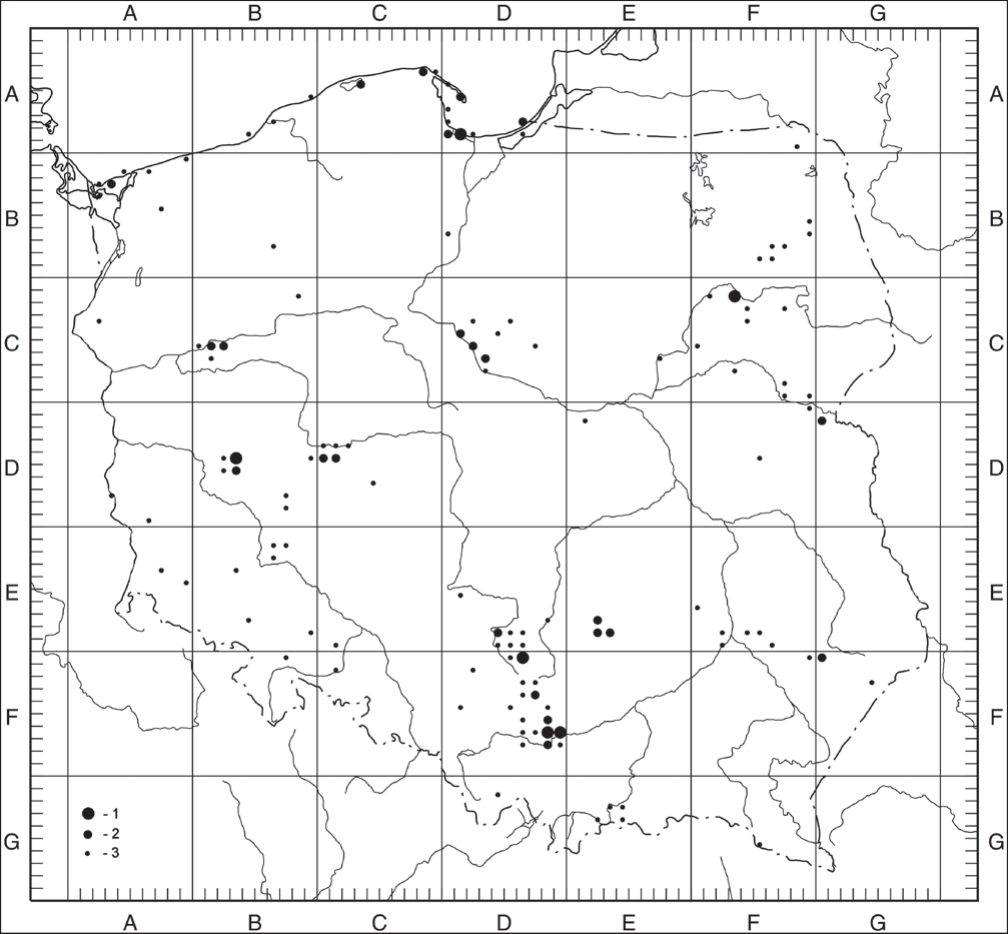
Collective distribution of Taraxacumsect.Erythrosperma species in Poland; **1** – 5–6 species per 10 km × 10 km square, **2** – 3–4 species per 10 km × 10 km square, **3** – 1–2 species per 10 km × 10 km square.

Of the 14 examined species of Taraxacumsect.Erythrosperma in Poland, 7 are definitely rare, known from 3 to 13 localities to date. Three of them (*T.danubium*, *T.cristatum*, *T.sandomiriense*) are distributed in south-central Poland in relatively small areas, the next 3 are known from the north-eastern part of the country (*T.dissimile*) and the Baltic Sea seashore (*T.tortilobum*, *T.lacistophyllum*). Another rare species, *T.disseminatum*, is known from 11 localities scattered over a relatively large area. The other species from the section are fairly frequent (>20 localities), although they are known from no more than 50 localities. The most common is *T.scanicum*, known from 42 localities. As for the concentration of T.sect.Erythrosperma species-localities per grid square, the highest is obser­ved within the Kraków-Częstochowa Upland, the Gdańsk Coastland and the Wielkopolska Lowland (Fig. 8).

### ﻿Potential distribution modelling

The distribution model was performed for 11 species of dandelions. All the models show a high value of AUC (0.98 up to 1.00), which confirmed their reliability (Table 8). Variables with a relatively higher percentage of contribution in the MaxEnt models for the greatest number of species were bio11, bio3 and bio7 and, among soil factors, crf (Table 9).

**Table 8.** Area Under Curve (AUC) values for training and test data. The values shown are averaged over 20 replicate MaxEnt model runs.

Species	Training AUC	Test AUC
*T.bellicum*	0.98	0.98
*T.brachyglossum*	0.99	0.99
*T.cristatum*	0.99	0.99
*T.danubium*	1.00	0.99
*T.disseminatum*	0.99	0.99
*T.lacistophyllum*	1.00	0.99
*T.parnassicum*	0.99	0.99
*T.plumbeum*	0.99	0.99
*T.proximum*	0.99	0.99
*T.scanicum*	0.99	0.99
*T.tenuilobum*	0.99	0.98

**Table 9.** Variables' contribution (jackknife test) to training for model performance with only a particular variable, versus a model without a variable. The values shown are averaged over 20 replicate MaxEnt model runs.

			*T.bellicum*	*T.brachyglossum*	*T.cristatum*	*T.danubium*	*T.disseminatum*	*T.lacistophyllum*	*T.parnassicum*	*T.plumbeum*	*T.proximum*	*T.scanicum*	*T.tenuilobum*
Training gain	without variable	awcts	2.79	3.79	3.22	3.72	3.76	3.48	3.35	3.03	3.56	3.37	2.93
		bio10	2.78	3.85	3.29	3.78	3.78	3.46	3.36	3.07	3.53	3.35	2.95
		bio11	2.78	3.84	3.23	3.75	3.56	3.38	3.36	3.03	3.48	3.34	2.86
		bio18	2.70	3.74	3.04	3.70	3.78	3.46	3.31	3.02	3.53	3.36	2.89
		bio19	2.73	3.79	3.29	3.59	3.36	3.30	3.23	2.91	3.42	3.21	2.68
		bio3	2.80	3.77	3.26	3.77	3.78	3.49	3.29	3.07	3.56	3.32	2.75
		bio7	2.77	3.84	3.19	3.74	3.75	3.24	3.29	3.03	3.52	3.33	2.95
		cly	2.80	3.73	3.25	3.77	3.64	3.48	3.37	3.04	3.42	3.30	2.87
		crf	2.79	3.79	3.11	3.71	3.78	3.47	3.30	3.04	3.51	3.27	2.90
		orc	2.82	3.84	3.25	3.76	3.73	3.48	3.35	3.09	3.56	3.37	2.93
		pH	2.82	3.75	3.28	3.78	3.71	3.44	3.37	3.06	3.53	3.33	2.88
	only with variable	awcts	0.61	0.77	0.68	1.18	0.33	0.15	0.74	0.81	0.77	0.51	0.31
		bio10	1.17	0.69	0.44	0.81	0.44	0.55	1.01	0.91	0.99	1.12	0.55
		bio11	1.42	1.59	1.45	1.66	1.40	1.64	1.57	1.39	1.49	1.39	1.24
		bio18	1.02	1.51	1.16	1.00	0.46	0.70	0.94	0.62	0.81	0.69	0.62
		bio19	0.55	0.61	0.30	0.78	0.47	0.56	0.61	0.58	0.58	0.62	0.51
		bio3	1.16	1.67	1.39	1.64	0.94	0.57	1.66	1.10	1.25	1.09	1.04
		bio7	1.39	1.38	1.44	1.75	1.14	1.82	1.70	1.40	1.35	1.29	0.82
		cly	0.21	0.59	0.31	0.31	0.94	0.39	0.31	0.48	1.02	0.95	0.60
		crf	0.30	0.88	0.30	0.65	0.53	0.62	0.61	0.64	0.83	0.99	0.62
		orc	0.81	1.03	0.89	1.14	0.67	0.40	1.02	0.71	0.68	0.43	0.33
		pH	0.58	0.81	0.57	0.41	0.50	0.52	0.59	0.51	0.58	0.59	0.79

**Figure 9.**
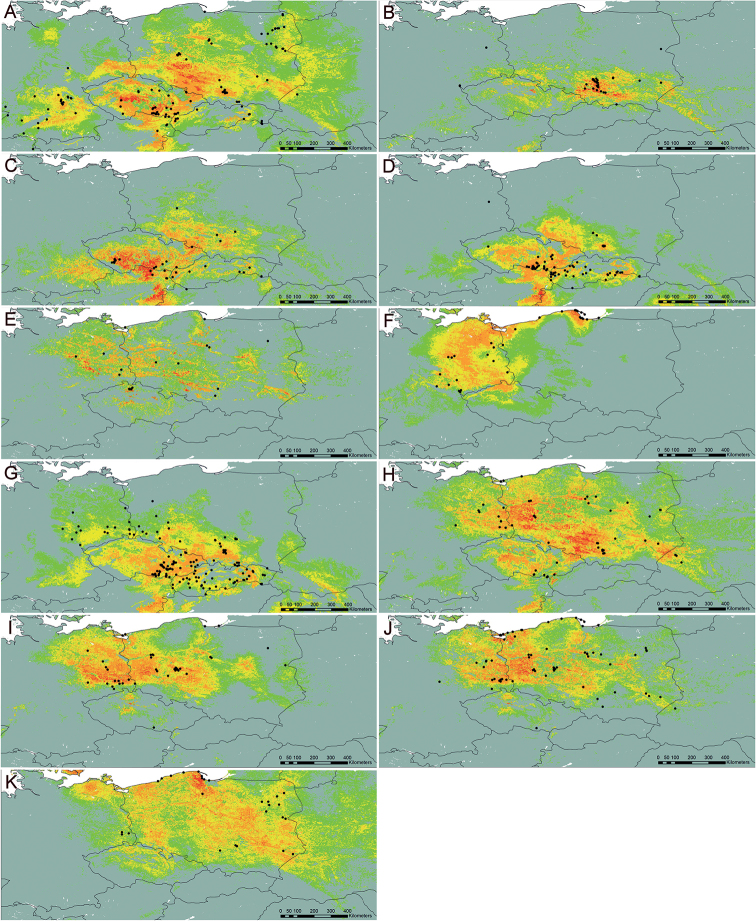
Models of the potential distribution of selected species of Taraxacumsect.Erythrosperma in central Europe **A***T.bellicum***B***T.brachyglossum***C***T.cristatum***D***T.danubium***E***T.disseminatum***F***T.lacistophyllum***G***T.parnassicum***H***T.plumbeum***I***T.proximum***J***T.scanicum***K***T.tenuilobum*; probability of occurrence: very low (grey), low (green), medium (yellow), high (orange), very high (red).

**Figure 10.**
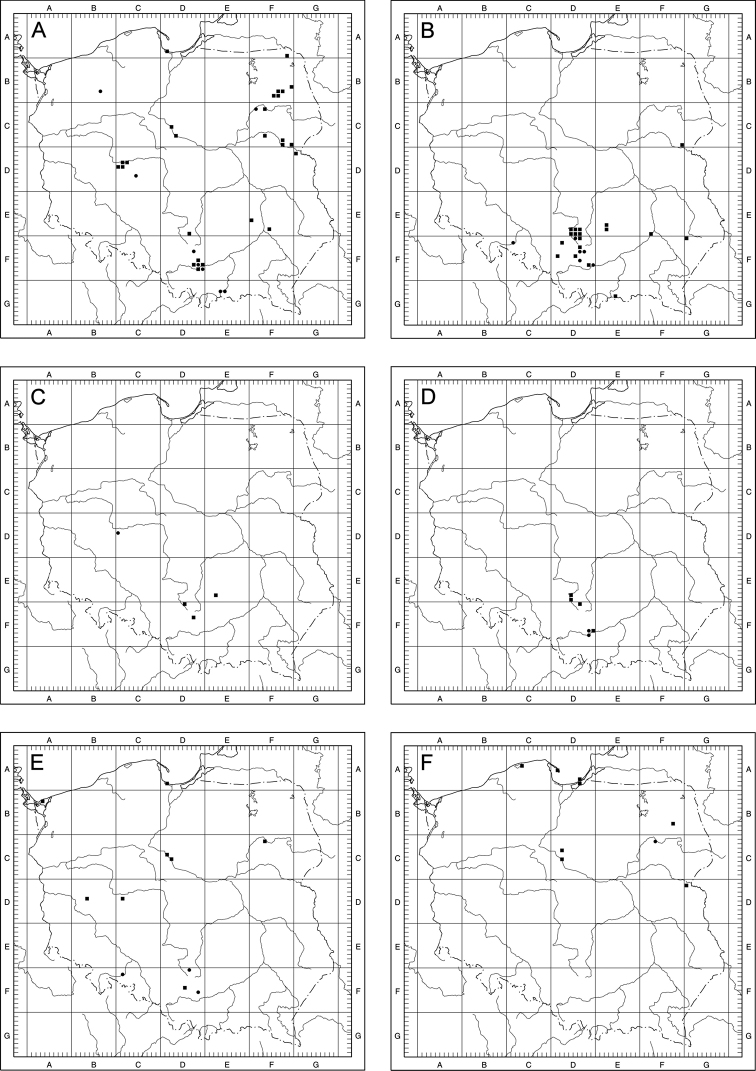
Distribution maps of Taraxacumsect.Erythrosperma in Poland **A***T.bellicum***B***T.brachyglossum***C***T.cristatum***D***T.danubium***E***T.disseminatum***F***T.dissimile*; black square – localities recorded during field studies, black circle – other localities known from herbarium data.

For most species, the area of high and very high probability of occurrence is quite large, indicating that these species may be much more common in Central Europe than previously thought, and their poorly recognised distribution is an effect of insufficient study. Such species include *T.bellicum*, *T.cristatum*, *T.danubium*, *T.parnassicum* or *T.plumbeum*. All of these species are characterised by a similar pattern of potential distribution, covering steppe regions of Central Europe, from southern (Bavaria) and north-eastern Germany (areas on the middle and lower Elbe river valley), through central and southern Czech Republic (including Moravia), northern and central Slovakia, north-eastern Austria (on the Danube), the highlands of Silesia and Central Poland, the valleys of the lower Odra and Vistula rivers, to eastern Poland, and in the case of some species also south-western Ukraine (Fig. 9A, C, D, G, H). For *T.brachyglossum*, the general pattern of distribution is similar, but the area of high probability of occurrence is much smaller (Fig. 9B). Three species (*T.lacistophyllum*, *T.proximum*, *T.scanicum*), are characterised by a potentially more concentrated range in the north-western part of Poland (mostly Pomerania and Silesia) and eastern Germany (the middle Elbe river valley, Saxony and Brandenburg), (Fig. 9F, I, J). This is especially noticeable in the case of *T.lacistophyllum*, which in Poland probably occurs only in Pomerania, but is likely much more common in Germany (Fig. 9F). Two other species, *T.disseminatum* and *T.tenuilobum*, show rather scattered potential distribution patterns, with high and very high probability of occurrence in central and northern Czech Republic, eastern Germany (the middle Elbe river valley, Saxony and Brandenburg) to Pomerania (both in Germany and Poland), the valley of the lower Odra and the Vistula, highlands in Silesia, central, southern, and eastern Poland, as well as western Ukraine (Fig. 9E, K). In the case of *T.dissimile*, *T.sandomiriense*, and *T.tortilobum*, we were unable to construct reliable models due to an extremely small number of known localities (Figs 10F, 23E, 36B).

### ﻿Remarks on plant collection and species identification

Determination of species representing the section Erythrosperma can be difficult for beginners, who are not familiar with the morphological variability that is observed in the field. Except for some features within the inflorescence, most of the measurable features are characterised by a very wide range of variability. During determination, it is extremely important to carefully analyse qualitative features, such as the absence or presence of pollen; the shape, colour and arrangement of the outer bracts; the shape of the capitulum, the shape of the terminal lobe, side lobes, and interlobes; the pre­sence or absence of teeth on lobes and in the interlobes; the colour and hairiness of the leaves and peduncles; the colour of the flowers and achenes. Some quantitative features are also important, e.g. the number of side lobes and outer bracts. Species-specific features are best visible in the field, in numerous populations, preferably in full flowering/beginning of fruiting time (in Poland, this period begins in the se­cond half of April in the Uplands and in the first week of May in the north and in the mountains; in Poland this period overlaps with the flowering of *Prunusspinosa*). Rainless and warm springs are favourable for field research. If spring is rainy and cold, small plants from the Erythrosperma section are usually overgrown by grass and other perennials; they then lose their diagnostic features and are hardly noticeable from a greater distance. In the field, it is worth noting features such as the arrangement and colour of outer bracts, the colour of petioles, and the colour and shape of the capitulum. It is crucial to carefully dry collected plants after the harvest; this makes later determination much easier. All data should be taken into consideration during determination, and the specimen should be compared both with the identification key and the description.

### ﻿Key to species identification

**Table:** 

1	Pollen grains present, and numerous	**2**
–	Pollen grains absent or sparse (a few grains on some stigmas)	**12**
2	Achenes brown-red, purple-brown or yellowish brown-red	**3**
–	Achenes in another colour (without red admixture)	**11**
3	Outer bracts narrowly lanceolate, without or rarely with a very narrow, barely visible hyaline margin (up to 0.05 mm broad)	***T.tenuilobum***
–	Outer bracts lanceolate to ovate, with a clearly visible (sometimes very narrow) white hyaline margin (0.05–0.3 mm broad)	**4**
4	Distal margin of the inner leaves' lateral lobes entire or with occasional teeth at lower lobes	**5**
–	Distal margin of the inner leaves' lateral lobes usually dentate or denticulate, rarely incised	**7**
5	Outer bracts 4–6 mm long and 1.5–2.5 mm broad; regularly spreading to quite regularly arranged and recurved, corniculate	***T.danubium***
–	Outer bracts 7–9 mm long and 2–3 mm broad	**6**
6	Leaves greyish-green; capitulum light yellow, convex, outer bracts elegantly arcuate spreading, corniculate	***T.lacistophyllum***
–	Leaves dark green; capitulum dark yellow, usually opening partly, outer bracts spreading to erect, moderately corniculate	***T.brachyglossum***
7	Outer bracts mostly recurved, corniculate; terminal lobe of the inner leaves usually prolate	**8**
–	Outer bracts differently positioned (erect, subspreading, arcuate-reflexed), with or without corniculation; terminal lobe of the inner leaves triangular or subsagitate, quite often lingulate/lobulate	**10**
8	Terminal lobe of the inner leaves denticulate at the base; leaves usually with 3–4 pairs of lateral lobes	***T.cristatum***
–	Terminal lobe of the inner leaves without teeth at the base, at most incised; leaves with up to 6 pairs of lateral lobes	**9**
9	Lateral lobes of the inner leaves usually dissected; outer bracts grey-green, quite often suffused red-violet, recurved or patent	***T.scanicum***
–	Lateral lobes of the inner leaves narrowly triangular, acute; outer bracts usually red-violet, often regularly recurved	***T.bellicum***
10	Achenes red-brown, 3.5–4.1 mm long (incl. 1.0–1.4 mm long, cylindrical cone); leaves usually 3–4 times longer than wide, lateral lobes' distal margins strongly dentate and often incised	***T.disseminatum***
–	Achenes yellowish light red-brown, 3–3.6 mm long (incl. 0.6–0.8 mm long, subconical cone); leaves up to 7 times longer than wide, lateral lobes' distal margins often denticulate	***T.plumbeum***
11	Achenes pale grey-brown; outer bracts grey-green suffused with purple, loosely adpressed to obliquely spreading	***T.tortilobum***
–	Achenes brown; outer bracts purplish green, recurved	***T.sandomiriense***
12	Achenes yellowish-greyish-brown	***T.dissimile***
–	Achenes brown-red	**13**
13	Outer bracts ovate to wide lanceolate, white hyaline margin distinct (0.1–0.2 mm broad)	***T.parnassicum***
–	Outer bracts lanceolate, hyaline margin indistinct (up to 0.05 mm broad)	***T.proximum***

#### ﻿ Taraxacum sect. Erythrosperma

Taxon classificationPlantaeAsteralesAsteraceae

(H. Lindb.) Dahlst., Acta Fl. Sueciae 1: 36. 1921.

5A0952A6-D607-5F84-9C4A-C93ED9FF20F5

Taraxacum [unranked] ErythrospermaH. Lindb., Acta Soc. Fauna Fl. Fenn. 29(9): 18. 1908. Basionym.
≡ Taraxacumsubsect.Erythrosperma (H. Lindb.) Schischk. In Komarov, Fl. SSSR 29: 497. 1964.
= Taraxacumsect.Dissimilia Dahlst., Acta Florae Sueciae 1: 37. 1921. Type: Taraxacumdissimile Dahlst.
= Taraxacumsect.Fulva M. P. Christ., in Gröntved et al., Botany of Iceland 3(3): 253. 1942. Type: Taraxacumfulvum Raunk.
= Taraxacumsect.Proxima Doll, Wiss. Z. Univ. Rostock, Reihe Math.-Naturwiss. 17: 330. 1968. Type: Taraxacumproximum (Dahlst.) Raunk. (≡ T.erythrospermumsubsp.proximum Dahlst.).

##### Type.

Designated by Doll, 1974: 60; see Kirschner and Štěpánek (1987), Štěpánek and Kirschner (2012): *Taraxacumrubicundum* (Dahlst.) Dahlst. (T.erythrospermumsubsp.rubicundum Dahlst.); lectotype in S, designated by Doll 1973: 19: “Stockholm, Djurgardsfrescati”, 10 June 1898, Dahlstedt.

###### ﻿Overall description of section Erythrosperma

Plants mostly small to middle-sized, often forming a tunic of dried leaf leftovers. Leaves usually deeply lobed with narrow lobes and petioles. Scapes often slender, thin. Outer bracts usually small, often with cornicules. Capitulum mostly small, flowers often light yellow, sometimes golden yellow. Achenes mostly red, less often straw-coloured, strongly spinulose with a cylindrical or narrowly conical cone, narrow at the base. Plants bloom in early spring (from mid-April). Related to warm and sunlit habitats.

#### ﻿1. Taraxacum bellicum

Taxon classificationPlantaeAsteralesAsteraceae

Sonck, Memoranda Soc. Fauna Fl. Fenn. 59: 1. 1983.

88D5FA26-C93A-52FF-AFA2-5AC0D6D528BB

= Taraxacumprunicolor Mart.Schmid, Vašut & Oosterv., Feddes Repert. 115: 221. 2004. Type: Germany, Mittelfranken, Bayern. Erlangen, scattered sandy places under pinewood at the Erwin-Rommel-Wohnheim, Uni-Südgelände (MTB 6432/14; R 4429999 H 5493713), 1 May 2002, *M. Schmid* (holotype in M 0165146; isotypes in M 0165145, L 0538648, PRA, OL, STU, DR).

##### Type.

Finland, Lapponia inarensis, Jnari, church village, Miesniemi (lat. 68°52'N.), 7 July 1981, *C. E. Sonck* s.n. (holotype in H 591459; isotypes in CES, H 591458, S).

##### Description.

Plants small to middle-sized, 5–12(–15) cm tall. ***Leaves*** greenish, almost glabrous, (5–)7–15(–20) cm long and 1.5–3 cm wide, generally 3–6 times longer than wide, blades broadest in middle, with 3–5(–6) pairs of lateral lobes; lateral lobes of the inner leaves patent or slightly recurved, narrowly triangular, acute, with an entire or slightly dentate distal margin, proximal margin usually entire or with a few small teeth; lateral lobes of the outer leaves triangular, proximal margin usually entire, distal margin entire or slightly dentate; interlobes often toothed, sometimes blackish rimmed; terminal lobe of the inner leaves mostly with lingulate apex; terminal lobe of the outer and medial leaves triangular or slightly lingulate, usually packed lateral lobes below; petioles unwinged, pale purple to pale brown-purple. ***Scapes*** as long as or longer than leaves, green suffused with pale purple, hairy below capitulum. ***Capitulum*** slightly convex, 2.5–3.5 cm in diameter, dark yellow, outer strips grey brown; inner bracts greyish-green, corniculate, outer bracts usually 10–14, lanceolate, usually 6.5–9 mm long, 1–3 mm broad, usually red-violet, hyaline margin inconspicuous (up to 0.1 mm broad), regularly recurved, usually with small cornicules; stigmas yellow-greenish, yellow-green-blackish after drying, pollen present. ***Achenes*** greyish purple-brown, sparsely spinulose at the top, 3.5–4.0 mm long (incl. the 1.0–1.3 mm long, narrowly conical cone), rostrum 6.0–7.0 mm long, pappus white.

##### Flowering period.

April (May).

##### Habitat.

In the Polish lowlands this species occurs in a wide spectrum of habitats; mostly in dry, sandy semiruderal locations exposed to the sun, e.g. roadsides, paths in dry pine forests, sandy embankments, dry pastures, sandy paths in cemeteries (especially in Wielkopolska Lowland); plant communities with its participation are dominated by species characteristic to the *Molinio-Arrhenatheretea* and *Sedo-Scleranthetea* classes. In Podlchia (Klimaszewnica) it was reported in a pastured dry grassland with: *Achilleamillefolium*, *Agrostiscapillaris*, *Artemisiacampestris*, *Carexcaryophyllea*, *Cerstiumholosteoides*, *Erophilaverna*, *Galiummollugo*, *Knautiaarvensis*, *Luzulacampestris*, *Medicagofalcata*, *Myosotisstricta*, *Pilosellaofficinarum*, *Plantagolanceolata*, *Ranunculusbulbosus*, *Sedumacre*, Taraxacumsect.Taraxacum and *Trifoliumrepens*. In south Poland, this species often grows in small enclaves on exposed rocky slopes, rock shelves and fissures, in plant communities dominated by species characteristic to classes *Sedo-Scleranthetea*, *Festuco-Brometea* and *Molinio-Arrhenatheretea*. In Kraków-Częstochowa Upland (Kraków Kostrze place) this species was noted in irregular xerothermic grassland (evolved in an old limestone excavation), together with *Achilleamillefolium*, *Acinosarvensis*, *Alyssumalyssoides*, *Arenariaserpyllifolia*, *Artemisiavulgaris*, *Asperulacynanchica*, *Bromushordeaceus*, *Centaureastoebe*, *Dactylisglomerata*, *Echiumvulgare*, *Erodiumcicutarium*, *Euphorbiacyparissias*, *Festucapratensis*, *F.rubra*, *Galiumverum*, *Koeleriamacrantha*, Medicago×varia, *M.falcata*, *Plantagomedia*, *Potentillaarenaria*, *Sanguisorbaminor*, *Sedumacre*, *S.sexangulare*, *Stachysrecta*, Taraxacumsect.Taraxacum, *Thlaspiperfoliatum*, *Thymuskosteleckyanus*, *T.pulegioides*, *Trifoliumrepens*.

##### Somatic chromosome number.

24 (Wolanin and Musiał 2017).

##### General distribution.

Central European species reported in the Czech Republic, Austria, Germany, Poland, Switzerland, Slovakia, Ukraine and Finland (Uhlemann 2003; Horn et al. 2004; Schmid et al. 2004; Nobis et al. 2020b). Populations from Finland are most likely of anthropogenic origin (EURO+MED 2006-onwards).

##### Distribution in Poland.

Scattered localities, quite frequent in Podlachia, the western part of Lesser Poland and Greater Poland (Fig. 10A).

##### Specimens examined.

**BB76** – Borne Sulinowo, woj. zachodniopomorskie, 10 May 2005, *K. Rostański* (122777 KTU); **CD31** – Murzynowo Leśne, square close to shop, 52°09'17"N, 17°20'25"E, 17 April 2016, *M. Wolanin* (003506 UR); Solec, anti-flood embankment on the Warta River, 52°06'08"N, 17°19'53"E, 17 April 2016, *M. Wolanin* (003495 UR); **CD32** – Nowe Miasto nad Wartą, lawn in cemetery, 52°05'14"N, 17°23'57"E, 17 April 2016, *M. Wolanin* (003515 UR); **CD40** – Książ Wielkopolski, roadside in forest, 52°04'02"N, 17°14'44"E, 16 April 2016, *M. Wolanin* (003538 UR); **CD41** – between Radoszkowo and Chromiec, sandy embankment near disused railway track, 52°02'20"N, 17°16'30"E, 16 April 2016, *M. Wolanin* (003519 UR); **CD64** – Las Taczanowski near Ostrów Wielkopolski, 2 May 2013, *A. Czarna* (POZNB); **DA81** – Gdańsk (Stogi), roadside in pine forest, 54°22'31"N, 18°43'27"E, 7 May 2016, *M. Wolanin* (003488 UR); **DC52** – Dąbrówka, forest roadside, 52°53'42"N, 18°57'51"E, 29 April 2018, *M. Wolanin* (003446 UR); **DC52** – Wakole village vicinity, roadside in pine forest, 52°50'34"N, 18°57'07"E, 29 April 2018, *M. Wolanin* (003451 UR); **DC52** – Stare Rybitwy, roadside in pine forest, 52°50'01"N, 18°55'53"E, 29 April 2018, *M. Wolanin* (003457 UR); **DC73** – Włocławek, gap between pavement and kerb, 52°40'29"N, 19°05'12"E, 29 April 2018, *M. Wolanin* (003479 UR); **DE96** – Bystrzanowice, roadside of asphalt road No 46, 50°42'24"N, 19°30'53"E, 20 April 2016, *M. Wolanin* (003532 UR); **DF37** – Jaroszowiec near Olkusz, grassland near forest road, 12 May 1977, *H. Trzcińska-Tacik* (392532 KRAM); **DF58** – Duże Skałki, bonfire-burnt location on grassland, 50°11'19"N, 19°48'23"E, 30 April 2013, *M. Wolanin* (003345 UR); Żytnia Skała, grassland on rocks, 50°11'07"N, 19°48'05"E, 30 April 2013, *M. Wolanin* (003256 UR); Żytnia Skała, fissures in rock, 50°11'09"N, 19°48'04"E, 30 April 2013, *M. Wolanin* (003338 UR); Bolechowice, calcareous rocks, 1 May 1976, *H.*, *T.* & *J. Tacik* (392437, 392438, 575859 KRAM); Bolechowice, path, field road, 21 May 1976, *T. Tacik* (387575, 392460, 575854, 575893 KRAM); Dolina Kluczwody, calcareous rocks, 4 May 1957, *W. Wojewoda* (0129483 KRA); N of the village Biały Kościół, grassland on calcareous rock, 10 May 2013, *M. Nobis* (KRA); **DF67** – Czułów, grassland on rock, SW slope, 50°04'02"N, 19°41'39"E, 20 April 2015, *M. Wolanin* (003282 UR); **DF68** – Kraków (Bielany), forest clearing near road, 27 April 1975, *T. Tacik* (575853 KRAM); between Kryspinów and Bielany, sunny hill, 16 May 1976, H., *T.* & *J. Tacik* (392443 KRAM); **DF69** – Kostrze (Kraków), roadside, quarry, 50°02'18"N, 19°52'10"E, 19 April 2015, *M. Wolanin* (003295 UR), Pychowicka Górka, grassland on rocky-humus soil, 50°01'50"N, 19°53'00"E, 29 April 2013, *M. Wolanin* (003378 UR); Kraków, limestone hill near Pychowice, 8 May 1953, *T. Tacik* (575869 KRAM); Kraków (Zakrzówek), sunny hill, 5 August 1976, *T. Tacik* (392451 KRAM); Las Wolski (Kraków), calcareous rocks, 4 May 1954, *A. Jasiewicz* (437763 KRAM); Pychowice, hill, 11 May 1975, *T. Tacik* (392445 KRAM); **DF78** – Piekary (Kraków), grassland on rock, S slope, 50°00'54"N, 19°47'39"E, 19 April 2015, *M. Wolanin* (003269 UR); Tyniec Podgórki (Góra Wielkanoc), grassland on rocky-humus soil, 50°01'01"N, 19°48'55"E, 29 April 2013, *M. Wolanin* (UR 003337); Tyniec, Juranda ze Spychowa Street, calcareous rocks in former excavation, 50°00'34"N, 19°48'55"E, 29 April 2013, *M. Wolanin* (003366 UR); Piekary Tynieckie, grassland on rock, 20 April 1975, *T. Tacik* (575852 KRAM); Tyniec, hill, 14 May 1967, *J. Błaszczak* (063403 KRA); **DF79** – Kraków (Borek Fałęcki), pine forest, 7 May 1976, *T. Tacik* (392439 KRAM); Podgórki Tynieckie, sunny hill, 11 May 1976, *T. Tacik* (570163 KRAM); Podgórki Tynieckie, Biedzina hill, SW slope, 8 May 1979, *H. Trzcińska-Tacik* (601089 KRAM); **EG23** – Łącko, dry grassland close to Dunajec River, 3 May 1970, *K. Towpasz* (80794 KRA); Maszkowice n. Dunajcem, 13 May 1970, *K. Towpasz* (0123940 KRA); **EG24** – Kadcza (Kotlina Sądecka), rocky slope above road, 20 May 1970, *K. Towpasz* (80793 KRA); **FA98** – Gulbieniszki, dry grassland on SW slope of Góra Cisowa hill, 54°15'05"N, 22°54'33"E, 1 May 2018, *M. Wolanin* (003462 UR); Jeleniewo, pasture on dry rocky SW slope, 54°11'38"N, 22°54'50"E, 1 May 2018, *M. Wolanin* (003444 UR); **FB69** – Zabiele-Kolonia, sandy roadside, 53°33'30"N, 22°59'18"E, 24 April 2016, *M. Wolanin* (003526 UR); **FB76** – Klimaszewnica, sandy roadside, 53°28'50"N, 22°30'48"E, 24 April 2016, *M. Wolanin* (003524 UR); **FB77** – between Goniądz and Szafranki, dry pasture, 53°29'16"N, 22°42'30"E, 24 April 2016, *M. Wolanin* (003529 UR); Osowiec, dry sandy roadside near fort, 53°29'29"N, 22°38'20"E, 24 April 2016, *M. Wolanin* (003528 UR); **FB85** – Chrzanowo, sandy square close to excavation, 53°23'27"N, 22°21'23"E, 24 April 2016, *M. Wolanin* (003492 UR); **FB86** – Klimaszewnica, pastured grassland on sandy hill, 53°28'01"N, 22°30'07"E, 24 April 2016, *M. Wolanin* (003525 UR); **FC11** – Czartoria, hillock close to river, 8 May 2016, *T. Grużewska* (MPD); **FC13** – Piątnica (Fort Łomża), pastured grassland, 53°11'50"N, 22°06'53"E, 25 April 2016, *M. Wolanin* (003534 UR); **FC73** – Poniatowo, sandy roadside, 52°38'26"N, 22°02'44"E, 26 April 2016, *M. Wolanin* (003522, 003523 UR); Przewóz Nurski nad Bugiem, sandy roadside, 52°39'51"N, 22°17'30"E, 26 April 2016, *M. Wolanin* (003565 UR); **FC87** – Arbasy, sandy roadside, 52°30'39"N, 22°32'26"E, 26 April 2016, *M. Wolanin* (003520, 003521 UR); **FC97** – between Pustkowice and Minczew, sandy side of asphalt road, 52°27'16"N, 22°34'47"E, 26 April 2016, *M. Wolanin* (003535 UR); **FC99** – Anusin, sandy roadside at pine forest edge, 52°23'45"N, 22°53'46"E, 25 April 2016, *M. Wolanin* (003308 UR); Siemiatycze-Stacja, sandy roadside, 52°23'32"N, 22°56'10"E, 25 April 2016, *M. Wolanin* (003527 UR); **FE60** – Podgrodzie near Ćmielów, xetothermic grassland on SW slope, 50°54'24"N, 21°32'44"E, 17 April 2012, *M. Wolanin* (003396 UR); Podgrodzie near Ćmielów, xerothermic grassland on rock outcrop, 50°54'24"N, 21°32'44"E, 17 April 2012, *M. Wolanin*, *M. Nykiel* (003397 UR); **FE84** – between Zaklików and Lipa, sandy location at pine forest edge, 50°42'41"N, 22°04'43"E, 19 April 2019, *M. Wolanin* (003585 UR); **GD10** – Serpelice, lawn, 52°16'49"N, 23°03'01"E, 25 April 2016, *M. Wolanin* (003530 UR).

##### Notes.

**Figure 11.**
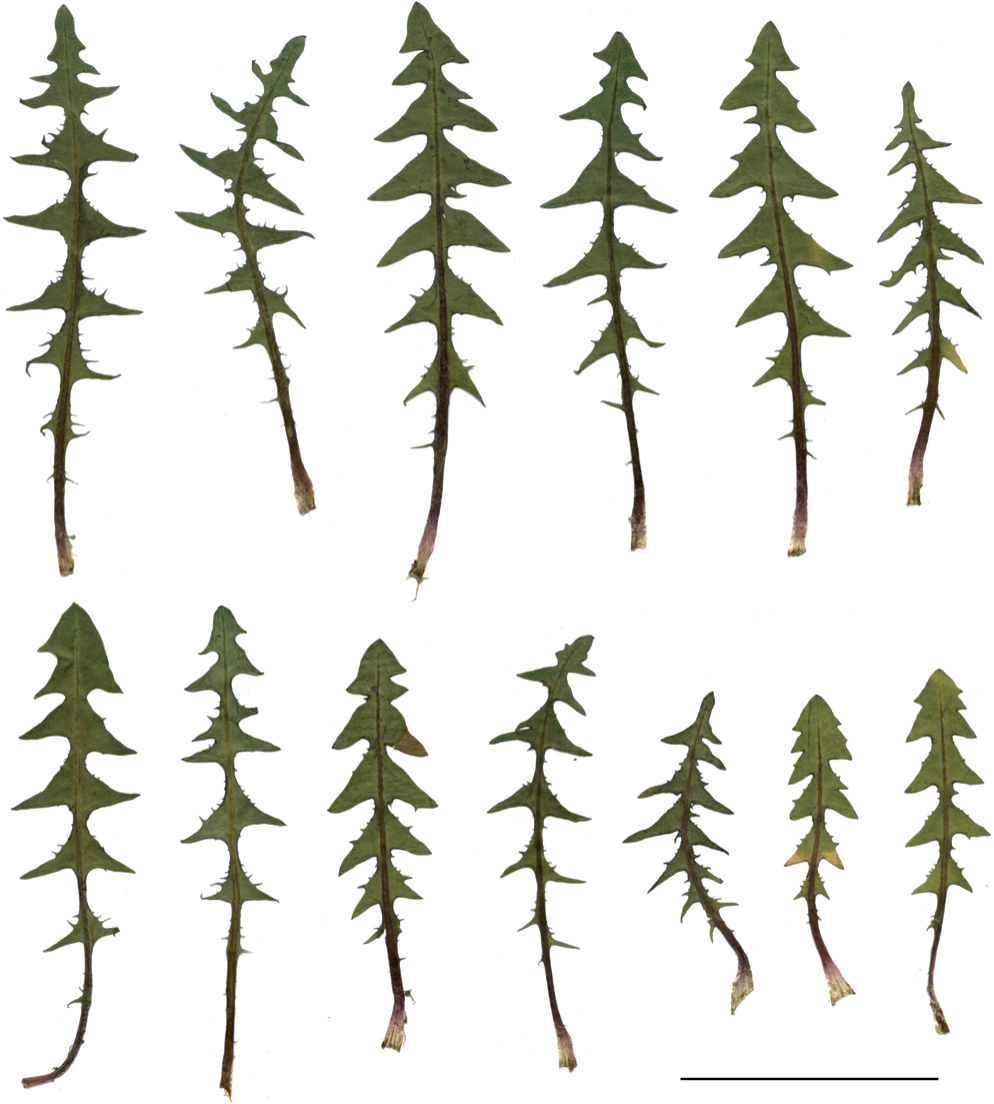
Variation in leaf shape in *T.bellicum*; locality – Nowe Miasto nad Wartą (*M. Wolanin* 2016 UR). Scale bar: 5 cm.

**Figure 12.**
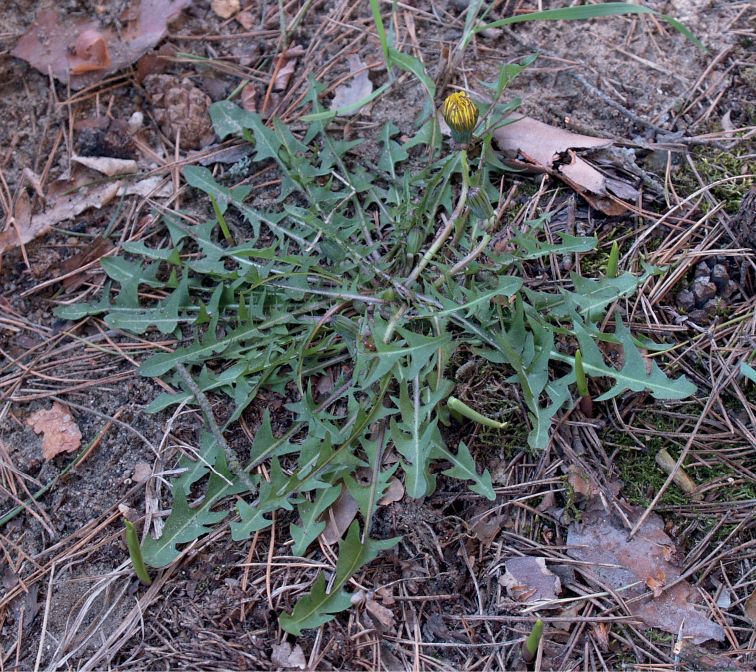
*Taraxacumbellicum*; locality – between Zaklików and Lipa, 2019, photo by M. Wolanin.

The species shows high morphological variability within leaf shape and the position and colour of outer phyllaries. This variability is evident among the populations from the Polish lowlands, often found in semi-shaded semi-ruderal and ruderal habitats such as sandy and gravely roadsides, backyards, sandy roads and paths in the forests and thickets. Features typical of the species, such as regularly recurved, red-purple, narrowly-edged outer phyllaries, or the distinct lingously elongated apex of the inner leaves terminal lobe, are well visible in specimens growing in stable, dry and full sun habitats, e.g. in sandy grasslands and rock grasslands in the south of Poland. Due to high morphological plasticity, the species can sometimes be confused with *T.scanicum*, which differs from *T.bellicum*, e.g. outer phyllaries are distinctly bordered (0.1–0.2 mm), mostly green, and the leaves' side lobes are regularly incised (Figs 11, 12).

#### ﻿2. Taraxacum brachyglossum

Taxon classificationPlantaeAsteralesAsteraceae

(Dahlst.) Raunk., Dansk Exkurs.-Fl., ed. 2: 257. 1906.

C03772D8-216A-50F9-AC1B-F1EF675AB889

Taraxacum erythrospermum subsp. brachyglossum Dahlst., Bot. Not., 1905: 170. 1905. Basionym.

##### Type.

Sweden, Stockholm, Bergian Bot. Garden, sunny lawn, 4 June 1904, *H. Dahlstedt* (lectotype, selected by G. Haglund and designated by Doll 1973: 53, in S).

##### Description.

Plants usually small-sized, 5–10 cm tall. ***Leaves*** dark green, somewhat glossy, almost glabrous or with few barely visible hairs, approximately 3–8(–10) cm long and (1–)1.5–2.5(–3.5) cm wide, usually are 3–4 times longer than wide, blades narrowly oblanceolate, usually broadest in upper 1/3, with 4–5 pairs of lateral lobes; lateral lobes opposite to remote, lateral lobes of the inner leaves narrowly triangular, recurved, the ends somewhat bent, distal margin usually entire, lower lobes slightly dentate, somewhat convex, proximal margin usually entire, often with a distinct tooth at the base; lateral lobes of the outer leaves triangular, entire, usually with a distinct tooth at the proximal margin base; interlobes usually toothed; terminal lobe of the inner leaves tripartite, often shortly lingulate and entire on the margins; terminal lobe of the outer leaves triangular or tripertite and shortly lingulate; petioles unwinged, mode­rately purplish. ***Scapes*** as long as or shorter than leaves, somewhat hairy. ***Capitulum*** often partially open, 1.5–2 cm, dark yellow, outer strips blackish-violet; inner bracts dark green, glaucous, usually with lumps or small cornicules; outer bracts usually 12–15, broadly lanceolate, usually 7–9 mm long, 2–3 mm broad, greyish-purple, with a narrow white hyaline margin (ca. 0.1 mm broad), spreading to erect, moderately corniculate; stigmas greyish-green, pollen present. ***Achenes*** brownish-red, spinulose above, 3.3–3.7 mm long (incl. the 0.8–1.2 mm long, narrowly conical cone), rostrum 7–9 mm long, pappus white.

##### Flowering period.

April–May.

##### Habitat.

Species associated mainly with thermophilic rock grasslands, occurring most often in pastured or trampled places. Moreover, this species is sometimes found in dry and sunny ruderal habitats such as railway tracks or mine slags. *T.brachyglossum* was reported in plant communities accompanied by species characteristic to the *Sedo-Scleranthetea*, *Festuco-Brometea* and *Molinio-Arrhenatheretea* classes. In Kraków-Częstochowa Upland (Olsztyn place) this species grew in a rock grassland together with *Acinosarvensis*, *Alliummontanum*, *Alyssumalyssoides*, *Arenariaserpyllifolia*, *Artemisiacampestris*, *Asperulacynanchica*, *Brizamedia*, *Carexcaryophyllea*, *Centaureastoebe*, *Dianthuscarthusianorum*, *Erysimumodoratum*, *Euphorbiacyparissias*, Helianthemumnummulariumsubsp.obscurum, *Hypericummaculatum*, *Jovibarbasobolifera*, *Libanotispyrenaica*, *Medicagofalcata*, *Phleumphleoides*, *Pilosellaofficinarum*, *Pimpinellasaxifraga*, *Poacompressa*, *P.pratensis*, *Potentillaarenaria*, *Sanguisorbaminor*, *Sedumacre*, *S.sexangulare*, *Sileneotites*, *Stachysrecta*, *Teucriumbotrys*, *Vincetoxicumhirundinaria*. In Pieniny Mts (Jaworki) we reported this species in a pastured rock grassland accompanied by: *Achilleamillefolium*, *Arabishirsuta*, *Arenariaserpyllifolia*, *Brizamedia*, *Campanularotundifolia*, *Carexflacca*, *C.montana*, *Cerastiumholosteoides*, *Convolvulusarvensis*, *Coronillavaria*, *Cruciataglabra*, *Cynosuruscristatus*, *Euphorbiacyparissias*, *Festucarubra*, *Fragariavesca*, *Galiummollugo*, *Geraniumcolumbinum*, *Jovibarbasobolifera*, *Juniperuscommunis*, *Knautiaarvensis*, *Leontodonhispidus*, *Linumcatharticum*, *Loliumperenne*, *Lotuscorniculatus*, *Medicagofalcata*, *M.lupulina*, *Phleumpratense*, *Pilosellaofficinarum*, *Pimpinellasaxifraga*, *Plantagolanceolata*, *P.media*, *Potentillaneumanniana*, *Prunellavulgaris*, *Prunusspinosa*, *Ranunculuspolyanthemos*, *Salviaverticillata*, *Sanguisorbaminor*, *Sedumacre*, *Taraxacumparnassicum*, *Thymuspulegioides*.

##### Somatic chromosome number.

24 (Małecka 1969; Wolanin and Musiał 2017).

##### General distribution.

Widely distributed European species reported in France, Ireland, Great Britain, Italy, Switzerland, Belgium, the Netherlands, Germany, Denmark, Austria, Croatia, Poland, Norway, Finland, Sweden, Romania, Croatia, Moldova, Ukraine and Estonia (Marklund 1938; Van Soest 1967; Doll 1973b; Tutin et al. 1976; Tacik 1980; Fedorov 1989; Mosyakin and Fedoronchuk 1999; Nikolić 2000; Uhlemann 2003; Wendt and Øllgaard 2015).

##### Distribution in Poland.

Scattered localities in S Poland, quite frequent in W part of Lesser Poland (Fig. 10B).

##### Specimens examined.

**CF11** – Nysa (Śląsk), May 1849, *M. Winkler* (WRSL); **DE84** – Kusięta, grassland on rock (path), 50°46'06"N, 19°16'16"E, 13 April 2014, *M. Wolanin* (003395 UR); Kusięta, grassland on rock, 50°46'03"N, 19°16'15"E, 12 April 2016, *M. Wolanin* (003486 UR); Olsztyn (Góra Zamkowa), grassland on rock outcrop, NW exposure, 50°44'55"N, 19°16'30"E, 13 April 2014, *M. Wolanin* (003360, 003454 UR); Olsztyn, grassland on rock, 50°44'55"N, 19°16'36"E, 12 April 2016, *M. Wolanin* (003487 UR); Olsztyn near Częstochowa, Góra Brodła hill, grassland on rock, 26 April 1975, *B. Baczyńska*, *I. Fibich* (017332, 117445 KTU); **DE85** – between Olsztyn and Przymiłowice, grassland on rock, 50°45'10"N, 19°17'05"E, 13 April 2014, *M. Wolanin* (003419, 003316 UR); Przymiłowice, grassland on rock, E slope, 50°45'22"N, 19°18'14"E, 13 April 2014, *M. Wolanin* (003317 UR); Przymiłowice, sandy road, 50°45'19"N, 19°17'48"E, 13 April 2014, *M. Wolanin* (003303 UR); **DE86** – Łutowiec near Mirów, grassland on the SW slope of a calcareous rock, 50°47'40"N, 19°27'19"E, 14 April 2014, *M. Wolanin* (003390 UR); **DE94** – Góra Sfinks hill, grassland on rock, 50°44'15"N, 19°16'17"E, 12 April 2016, *M. Wolanin* (003497 UR); **DE95** – Suliszowice, grassland on SW slope of calcareous rock, 50°40'19"N, 19°21'24"E, 13 April 2014, *M. Wolanin* (003290 UR); **DE96** – Bystrzanowice, parking lot close to road No 46, 50°42'25"N, 19°31'03"E, 20 April 2016, *M. Wolanin* (003413 UR); **DF05** – Przewodziszowice, grassland, 1983, *D. Kospanik* (037425 KTU); Żarki near Częstochowa, pine forest, 1994, *G. Pompa* (058136 KTU); **DF06** – Kroczyce, grassland on SW slope, 50°34'18"N, 19°31'47"E, 1 May 2013, *M. Wolanin* (003264 UR); Góra Zborów (Kroczyce), grassland on rock, 50°34'21"N, 19°31'49"E, 1 May 2021, *M. Wolanin* (003589 UR); Łutowiec, grassland on NW slope below calcareous outcrop, 50°37'42"N, 19°27'15"E, 14 April 2014, *M. Wolanin* (003361 UR); Mirów, grassland below calcareous rock, E slope, 50°36'51"N, 19°28'34"E, 14 April 2014, *M. Wolanin* (003377 UR); Mirów, path near castle, 50°36'53"N, 19°28'51"E, 14 April 2014, *M. Wolanin* (003389 UR); Rzędkowice, path on S slope of calcareous rock, 50°34'31"N, 19°29'07"E, 14 April 2014, *M. Wolanin* (003403 UR); Kroczyce, path on SW slope, 50°34'20"N, 19°31'48"E, 1 May 2013, *M. Wolanin* (003277 UR); close to Jaskinia Głęboka near Kroczyce, old excavation, 50°34'31"N, 19°31'26"E, 1 May 2021, *M. Wolanin* (003588 UR); **DF12** – Miasteczko Śląskie, ruderal square close to railway track, 50°29'14"N, 18°55'14"E, 3 May 2016, *M. Wolanin* (003491 UR); **DF26** – Podzamcze (Ogrodzieniec), to the left of the castle, fissure in calcareous rock, 50°27'15"N, 19°33'03"E, 1 May 2013, *M. Wolanin* (003339 UR); Ryczów (Kolonia Podzamcze), calcareous rock, fissure in NW side, 50°27'05"N, 19°33'14"E, 12 April 2014, *M. Wolanin* (003408 UR); Centuria near Ogrodzieniec, sandy road between pines, 6 June 1975, *T. Tacik* (570164, 570165 KRAM); **DF36** – Pustynia Błędowska, 29 April 1977, *H. Trzcińska-Tacik* (392440 KRAM); **DF37** – near the village Klucze, sandy roadside, 23 May 1955, *T. Tacik* (392459 KRAM); **DF41** – Ruda Śląska, on top of mine dump, 50°15'33"N, 18°50'25"E, 2 May 2016, *M. Wolanin* (003499 UR); **DF45** – Sosnowiec (Maczki), sandy roadside, 50°15'29"N, 19°17'08"E, 2 May 2016, *M. Wolanin* (003490 UR); Sosnowiec Maczki, between railway tracks, 28 August 1979, *A. Sendek* (027734 KTU); Sosnowiec Maczki, sandy square in the valley of the B. Przemsza, 9 May 1978, *A. Sendek* (034398 KTU); **DF56** – North of the Trzebinia (near the Myślachowice village), sandy location at pine forest edge, 27 April 1952, *T. Tacik* (575844 KRAM); **DF68** – Nielepice, fissures on top of calcareous rock, 50°06'20"N, 19°42'23"E, 12 April 2014, *M. Wolanin* (003407 UR); **DF69** – Krzemionki Dębnickie, dry grassy slopes, 8 May 1925, *Zabłocki* (169651 KRAM); Las Wolski, Przegorzały, calcareous rocks, 4 May 1954, *T. Tacik* (439037 KRAM); **EE72** – Miedzianka hill near Chęciny, grassland on rock, 50°50'47"N, 20°21'37"E, 11 April 2016, *M. Wolanin* (003485, 003496, 003540, 003542, 003561 UR); **EE82** – Grząby Bolmińskie, field road, 50°48'59"N, 20°21'17"E, 22 April 2016, *M. Wolanin* (003409 UR); **EG34** – Jaworki (Pieniny), pastured grassland on rock outcrop, 49°24'16"N, 20°32'36"E, 10 April 2014, *M. Wolanin* (003344 UR); Jaworki (Pieniny), pastured grassland on calcareous rock outcrop, 49°24'15"N, 20°32'37"E, 10 April 2014, *M. Wolanin* (003340 UR); Jaworki, grassland on rock, SE slope, 49°24'19"N, 20°32'37"E, 10 April 2014, *M. Wolanin* (003284 UR); **FC99** – Olendry, 52°23'07"N, 22°56'01"E, 25 April 2016, *M. Wolanin* (003541 UR); **FE92** – Kamień Łukawski, path on SE slope, 50°41'04"N, 21°47'10"E, 23 April 2016, *M. Wolanin* (003550 UR); Kamień Łukawski, path on loess slope, 50°41'04"N, 21°47'10"E, 19 April 2019, *M. Wolanin* (003584 UR); **GF00** – Szozdy, sandy road near railway track, 50°34'40"N, 22°56'11"E, 13 April 2019, *M. Wolanin* (003579, 003582 UR).

##### Notes.

**Figure 13.**
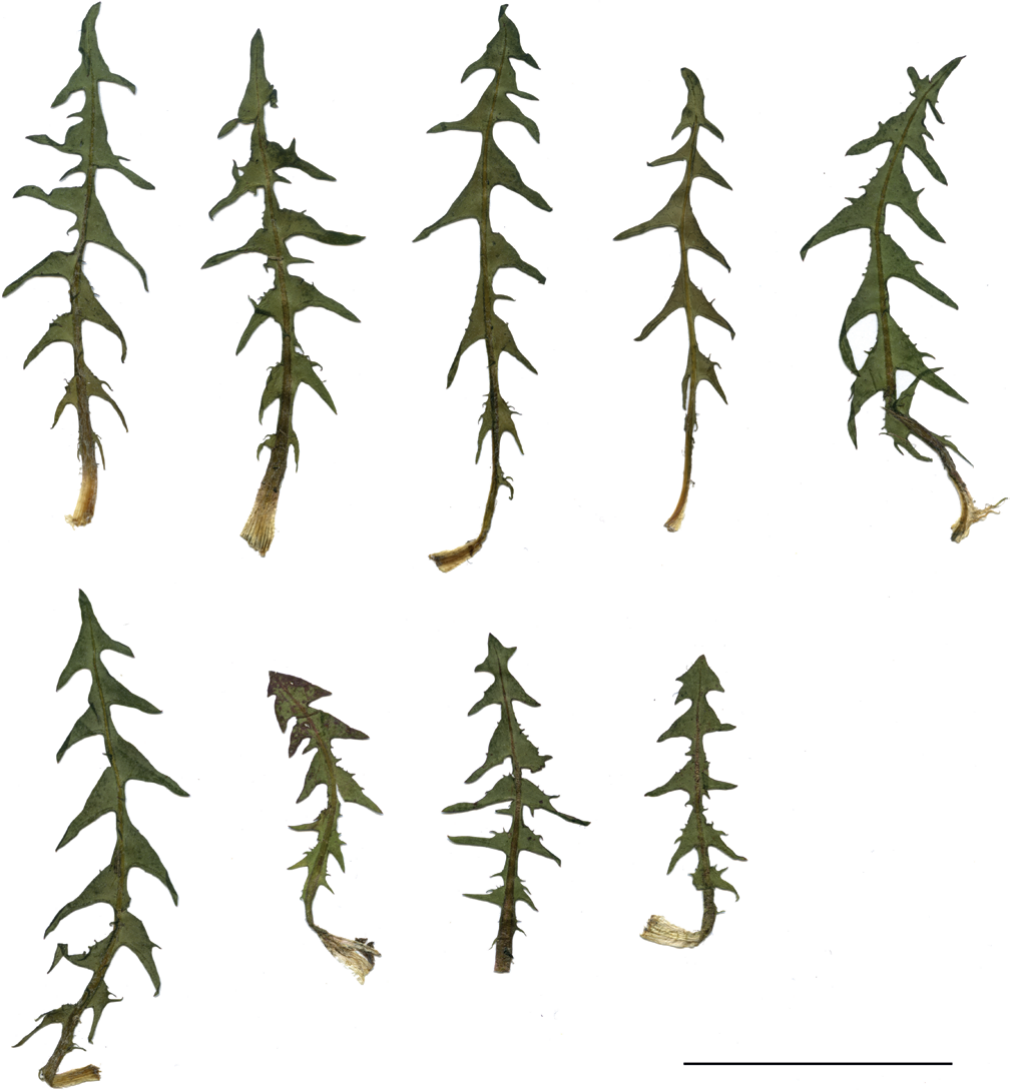
Variation in leaf shape in *T.brachyglossum*; locality – Kusięta (*M. Wolanin* 2016 UR). Scale bar: 5 cm.

**Figure 14.**
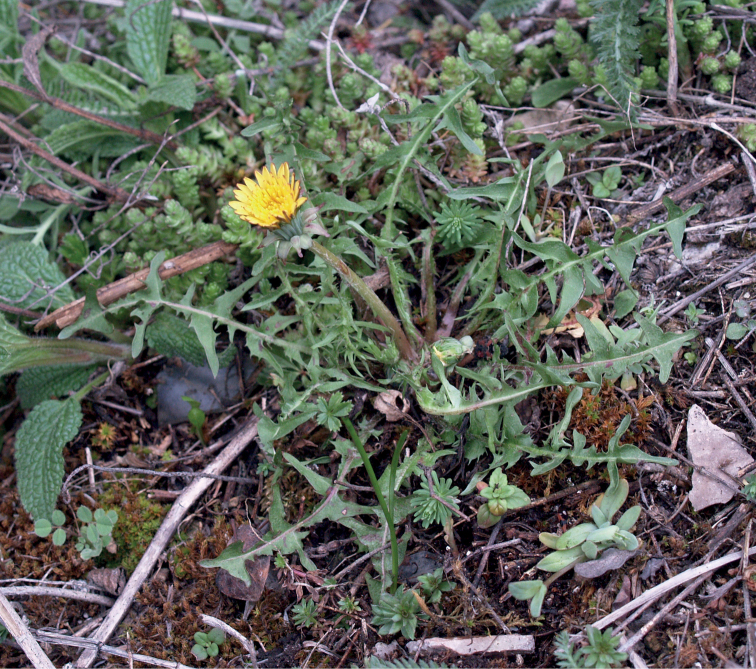
*Taraxacumbrachyglossum*; locality – Kusięta, 2016, photo by M. Wolanin.

Species distinguished by dark green leaves with side lobes narrowly triangle and bent downwards, outer phyllaries relatively wide, greyish-purple, narrowly bordered, and often a fully flowering capitulum partly-opening and dark yellow. Species morphologically variable; in specimens found in very dry, rocky habitats, the side lobes of the tripartite terminal lobe are very often positioned upwards, which often helps in their identification (Figs 13, 14).

#### ﻿3. Taraxacum cristatum

Taxon classificationPlantaeAsteralesAsteraceae

Kirschner, Štěpánek &Vašut, Preslia 77: 204. 2005.

5620B574-D23B-5757-84D7-D1EA88A453FD

[T.cristatum Kirschner & Štěpánek, nomen, in Chán et al. 2001: 151 et in Kirschner et al. 2002: 692].

##### Type.

Slovaciamerid.-orientalis, opp. Rožňava, pagus Krásnohorské Podhradie (Krasznahorkaváralja): in graminosis siccis prope viam ad ruinam castelli Krásna Hôrka, 1 May 2004, *R.J. Vašut*, *M. Vašutová* (holotype in PRA; isotypes in OL, PRC, herbarium R. J. Vašut).

##### Description.

Plants usually small, 5–10 cm tall. ***Leaves*** (pale) green, almost glabrous, approximately (3–)5–10 cm long and (1–)2–2.5(–3.5) cm wide, usually 4–5 times longer than wide, blades eliptical or oblanceolate, with 3–4 pairs of lateral lobes; lateral lobes mostly opposite; lateral lobes of the inner leaves narrowly triangular, falcate, with a dentate, convex distal margin, proximal margin entire or with a few teeth; lateral lobes of the outer leaves triangular, entire or somewhat denticulate at the distal margin; interlobes narrow and long, undulate or denticulate, often dark maculate; terminal lobe of the inner leaves prolate, lingulate and denticulate at the base; terminal lobe of the outer leaves triangular, prolate, undulate at the base; petioles unwinged, reddish-purple, almost glabrous. ***Scapes*** as long as or slightly lon­ger than leaves, almost glabrous or with few barely visible hairs. ***Capitulum*** convex, 2–2.5 cm in diameter, yellow, outer strips greyish-brown-purple; inner bracts greyish-green, often suffused with purple at the ends, corniculate; outer bracts usually 9–11, lanceolate, usually 6–8 mm long, 1.5–2 mm broad, pale green, suffused pale red-purple, with a white hyaline margin (0.05–0.1 mm broad), recurved and corniculate; stigmas olive-greyish, pollen present. ***Achenes*** purplish-brown, with thin spinules in the upper part, 3.5–4.0 mm long (incl. the 0.8–1.1(–1.3) mm long, narrowly conical pyramid), rostrum 5.5–7.1 mm long, pappus white.

##### Flowering period.

April (May).

##### Habitat.

In Bogucin and Grząby Bolmińskie we noted this species on field roads and paths among overgrown calcareous rock grasslands; in Przewodziszowice (Kraków-Częstochowa Upland) on the sandy dry roadside. In plant communities accompanied by *T.cristatum* we noted the species characteristic to the *Festuco-Brometea*, *Molinio-Arrhenatheretea*, *Sedo-Scleranthetea* and *Trifolio-Geranietea sanguinei* classes. In the lar­gest population of this species (Kraków-Czestochowa Upland, Bogucin place), it grew together with *Achilleamillefolium*, *Arrhenatherumelatius*, *Asperulacynanchica*, *Carexhirta*, *C.praecox*, *C.spicata*, *Cerastiumarvense*, *C.semidecandrum*, *Convolvulusarvensis*, *Coronillavaria*, *Dianthuscarthusianorum*, *Erysimumodoratum*, *Euphorbiacyparissias*, *Festucarubra*, *Fragariavesca*, *Hypericumperforatum*, *Knautiaarvensis*, *Libanotispyrenaica*, *Lotuscorniculatus*, *Medicagofalcata*, *Phleumphleoides*, *Pilosellaofficinarum*, *Plantagolanceolata*, *Poacompressa*, *P.pratensis*, *Potentillaarenaria*, *Ranunculusbulbosus*, *Sanguisorbaminor*, *Scabiosaochroleuca*, *Sedumacre*, *Silenenutans*, *Thymuspulegioides*, *Trifoliumrepens*, *Veronicachamaedrys*, *Violatricolor*.

##### Somatic chromosome number.

24 (Wolanin and Musiał 2017).

##### General distribution.

Central European species, reported in Austria, the Czech Republic, Poland, Slovakia and Hungary (Vašut et al. 2005).

##### Distribution in Poland.

Species very rare, found so far in Lesser and Greater Poland (Fig. 10C).

##### Specimens examined.

**CD40** – Książ Wielkopolski, false acacia forest in N part of town, “Torfica”, 2000, *A. Czarna* (POZNB); **DF05** – Przewodziszowice, dry roadside, 50°38'23"N, 19°23'24"E, 12 April 2016, *M. Wolanin* (003543, 003580 UR); **DF37** – Bogucin Mały, grassland on calcareous rock outcrop, SW exposition, 50°18'29"N, 19°34'16"E, 12 April 2014, *M. Wolanin* (003292 UR); **EE82** – Grząby Bolmińskie, field road, 50°48'46"N, 20°21'44"E, 22 April 2016, *M. Wolanin* (003305 UR).

##### Notes.

**Figure 15.**
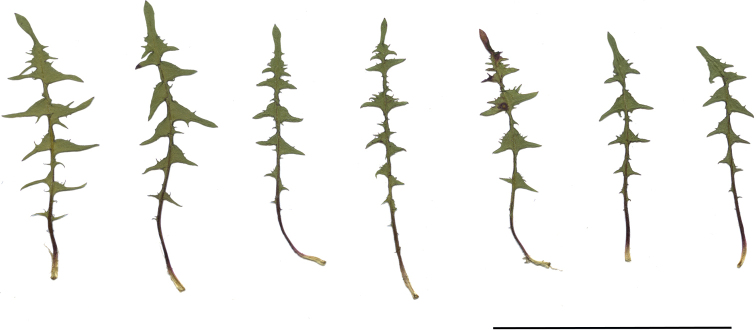
Variation in leaf shape in *T.cristatum*; locality – Grząby Bolmińskie (*M. Wolanin* 2016 UR). Scale bar: 5 cm.

**Figure 16.**
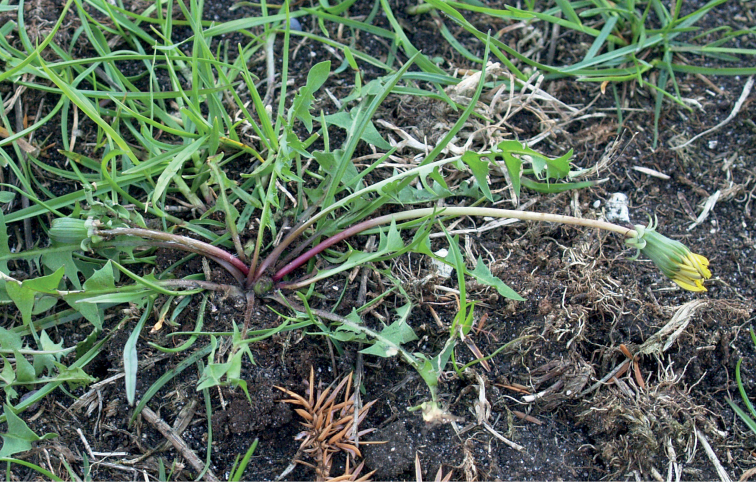
*Taraxacumcristatum*; locality – Grząby Bolmińskie, 2016, photo by M. Wolanin.

Species belonging to the *Scanicum* group, similar to the rest of the species from this group, with an asymmetrically incised terminal lobe. However, compared to *T.bellicum* and *T.scanicum*, the terminal lobe in *T.cristatum* is much more denticulate, as is the distal margin of the side lobes. *T.cristatum* may sometimes closely resemble *T.plumbeum* (especially individuals of *T.plumbeum* growing in extremely dry, rocky habitats), but it differs from it in its purple-brown achenes and lower number of pairs of side lobes (3–4) (Figs 15, 16).

#### ﻿4. Taraxacum danubium

Taxon classificationPlantaeAsteralesAsteraceae

A. J. Richards, Acta Fac. Rerum Nat. Univ. Comen., Bot. 18: 108. 1970.

9CFF6EB2-E86A-5CA9-9C49-28F2C1013445

≡ Taraxacumaustriacumvar.danubium (A. J. Richards) Doll, Feddes Repert. 84: 21. 1973.

##### Type.

Slovakia, Devínská Kobyla u Bratislavy, 1 May 1968, *A. J. Richards* (holotype in OXF).

##### Description.

Plants usually small, up to 10(–12) cm tall. ***Leaves*** greyish-green, dull, sparsely hairy, approximately 3–5(–7) cm long and (1–)1.5–2.5 cm wide, usually 3–4 times longer than wide, blades oblanceolate, usually broadest in upper 1/3, with 3–4 pairs of lateral lobes; lateral lobes opposite to remote; lateral lobes of the inner leaves patent, with a wide abruptly narrowed base and generally slightly widening at the apex, entire or with a few small teeth at the margin; lateral lobes of the outer leaves recurved and obtuse at the apex, entire or occasionally with a few small teeth at the margin; interlobes often with teeth; terminal lobe of the inner leaves triangular, often with a distinct short and obtuse tip; terminal lobe of the outer leaves triangular, obtuse; petioles narrowly winged, pale purplish. ***Scapes*** as long as or slightly longer than leaves, reddish-purplish, sparsely hairy in the upper part. ***Capitulum*** convex, yellow, 2–3 cm in diameter, ligules with greyish brown-red stripes; inner bracts greyish-green, corniculate; outer bracts usually 10–14, lanceolate, usually 4–6 mm long, 1.5–2.5 mm broad, greyish-green, quite often suffused purple, with a white hyaline margin 0.1(–0.2) mm broad, regularly spreading to quite regularly arranged and recurved, 4–6 mm long, 1.5–2.5 mm broad, corniculate; stigmas greyish-green, pollen present. ***Achenes*** dark brown-red, achene body densely spinulose above, 3.3–3.8 mm long (incl. the 0.7–1.0 mm long, narrowly conical cone).

##### Flowering period.

April (May).

##### Habitat.

In Poland, this species was observed only in grasslands and crevices of calcareous rocks. Plant communities associated with *T.danubium* were dominated by species characteristic to the *Sedo-Scleranthetea* and *Festuco-Brometea* classes. In Kraków-Częstochowa Upland (Olsztyn place) it was noted as growing together with *Alliummontanum*, *Anthyllisvulneraria*, *Arabishirsuta*, *Arrhenatherumelatius*, *Artemisiacampestris*, *Asperulacynanchica*, *Brizamedia*, *Carexcaryophyllea*, *Centaureastoebe*, *Cerastiumarvense*, *C.semidecandrum*, *Coronillavaria*, *Dactylisglomerata*, *Dianthuscarthusianorum*, *Erysimumodoratum*, *Euphorbiacyparissias*, *Fragariaviridis*, *Galiummollugo*, Helianthemumnummulariumsubsp.obscurum, *Juniperuscommunis*, *Luzulacampestris*, *Medicagofalcata*, *Phleumphleoides*, *Pilosellaofficinarum*, *Plantagolanceolata*, *P.media*, *Poacompressa*, *P.pratensis*, *Polygalacomosa*, *Potentillaarenaria*, *Ranunculusbulbosus*, *Rhamnuscathartica*, *Sanguisorbaminor*, *Scabiosaochroleuca*, *Sedumacre*, *S.sexangulare*, *Silenenutans*, *S.vulgaris*, *Thymuspulegioides*, *Veronicaspicata*, *Vincetoxicumhirundinaria*.

##### Somatic chromosome number.

24 (Wolanin and Musiał 2018).

##### General distribution.

Central European species reported in the Czech Republic, Hungary, Austria, Slovakia, North-Eastern Germany and Poland (Uhlemann 2003; Trávníček et al. 2010; Wolanin and Musiał 2018; Štěpánek and Kirschner 2023).

##### Distribution in Poland.

Very rare, so far only found in the western part of Lesser Poland (Fig. 10D).

##### Specimens examined.

**DE84** – Olsztyn, rock close to castle, 50°44'59"N, 19°16'47"E, 13 April 2014, *M. Wolanin* (003453); **DE94** – Góra Sfinks, grassland on rock, 50°44'15"N, 19°16'17"E, 12 April 2016, *M. Wolanin* (003483 UR); **DF06** – Mirów, grassland below castle (S slope), 50°36'50"N, 19°28'30"E, 14 April 2014, *M. Wolanin* (003469 UR); **DF68** – between Kryspinów and Bielanany, limestone hill, 16 May 1976, *H.*, *T.* & *J. Tacik* (387573 KRAM); **DF69** – Kostrze (Kraków), grassland on rock, 50°02'19"N, 19°52'09"E, 19 April 2015, *M. Wolanin* (003460 UR); Pychowicka Górka, grassland on rock, 50°01'50"N, 19°53'00"E, 29 April 2013, *M. Wolanin* (003464 UR); Pychowicka Górka, grassland on rock, 50°01'53"N, 19°52'48"E, 29 April 2013, *M. Wolanin* (003445 UR); Skały Twardowskiego, grassland on rock, 50°02'27"N, 19°54'15"E, 29 April 2013, *M. Wolanin* (003448 UR); Kostrze near Kraków, 27 April 1954, *W. Kurek*, *A. Jasiewicz* (439049 KRAM); Las Wolski, Przegorzały, calcareous rocks, 4 May 1954, *T. Tacik* (439037 KRAM); between Skotniki and Pychowice in the vicinity of Kraków, on dry hills, 22 May 1938, *J. Lilop* (036160, 036161 KRAM); on the Kostrze–Pychowice route, dry limestone hills, 27 April 1957, *H. Trzcińska-Tacik* (0378960 KRA); Pychowice near Kraków, calcareous rocks, 27 April 1954, *A. Jasiewicz* (155140 KRA); Pychowice, pasture, 23 April 1951, *K. Szczepanek* (111697 KRA); Pychowice, hillock, *J. Staszkiewicz* (407038 KRAM); behind Pychowice, limestone hillocks, 8 May 1953, *T. Tacik* (387179, 387571 KRAM); **DF78** – Podgórki Tynieckie, hill above water hole, 29 May 1975, *T. Tacik* (387181, 387182 KRAM); Podgórki Tynieckie, limestone hillock, 27 April 1951, *T. Tacik* (387180 KRAM); Tyniec, hillock, 14 May 1967, *J. Błaszczak* (063403 KRA).

##### Notes.

**Figure 17.**
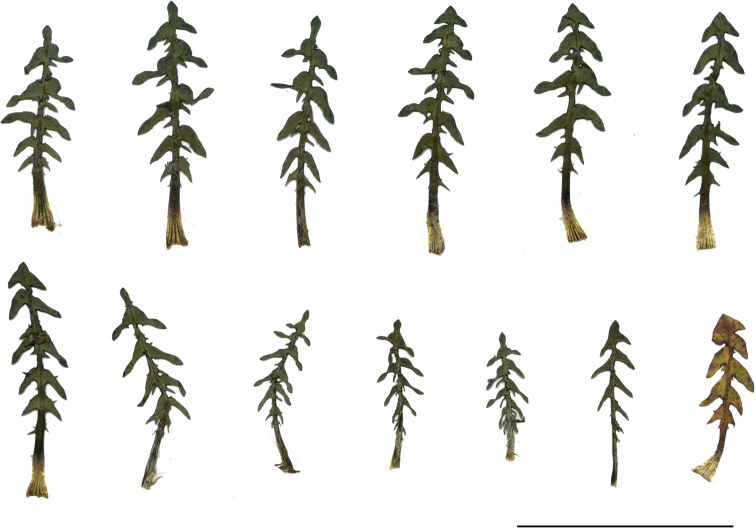
Variation in leaf shape in *T.danubium*; locality – Olsztyn (*M. Wolanin* 2016 UR). Scale bar: 5 cm.

**Figure 18.**
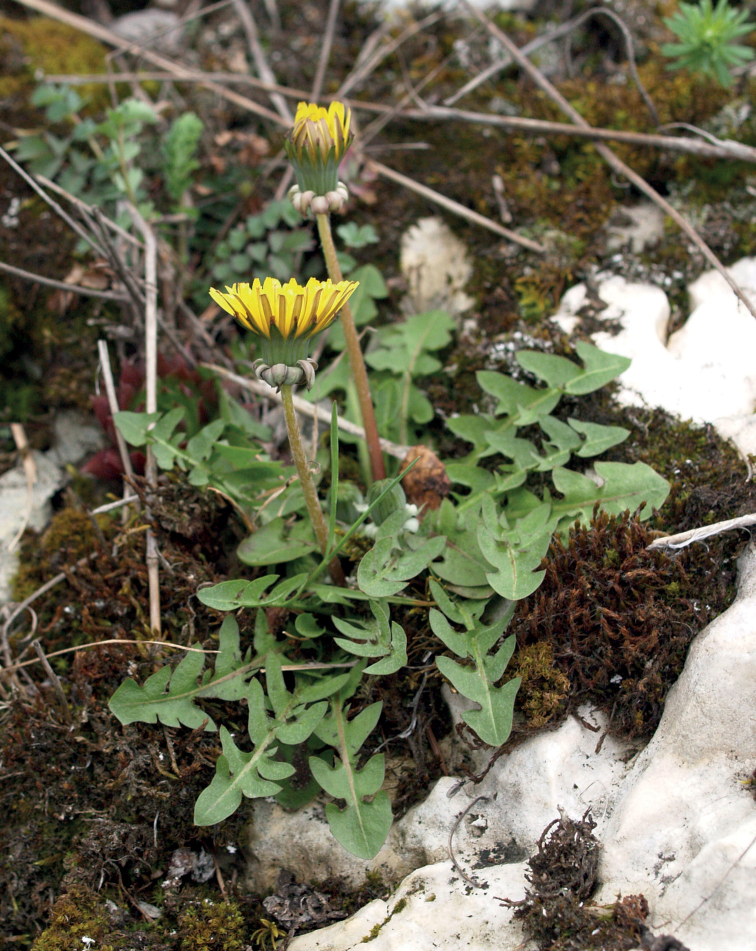
*Taraxacumdanubium*; locality – Olsztyn, 2016, photo by M. Wolanin.

Species quite small, with sparsely hairy and dull leaves, usually narrow interlobes and side lobes patent or slightly bent, often a little bloated near the ends. Juvenile specimens of *T.danubium* often have poorly split leaves in the upper part, which makes them similar to *T.parnassicum*, but due to the presence of pollen, the leaves of *T.danubium* are hairy and its outer phyllaries longer and wider, and therefore these two species can be easily distinguished. In the populations of *T.danubium* observed in Poland, the vast majority were young individuals with small rosettes with leaves shaped similarly to the external leaves of several-year-old specimens (Figs 17, 18).

#### ﻿5. Taraxacum disseminatum

Taxon classificationPlantaeAsteralesAsteraceae

G.E.Haglund, Svensk Bot. Tidskr. 41: 85. 1947.

1B65471C-55A8-5E2F-8CFA-EE925122DCD2

##### Type.

Sweden, Göteborg, 9 May 1943, *T. A. Borgvall* (holotype in S).

##### Description.

Plants middle to quite large-sized, 5–15(–20) cm tall. ***Leaves*** greyish-green, sparsely hairy on the upper side, approximately (5–)7–12(–15) cm long and (1.5)2–3(–4.0) cm wide, usually 3–4 times longer than wide, blades elliptical to oblanceolate, with 3–4(–6) pairs of lateral lobes; lateral lobes opposite to remote; lateral lobes of the inner leaves triangular, broad at the base, with a convex, strongly dentate and often incised distal margin, proximal margin usually entire or with a few teeth; lateral lobes of the outer leaves triangular, uniform, broad and short, with strong teeth at often incised and convex distal margin, proximal margin usually entire and slightly concave; interlobes narrow; terminal lobe of the inner leaves triangular, somewhat elongate, sometimes lingulate, denticulate on the upper margins; terminal lobe of the outer leaves triangular, subacute, entire or with a large tooth on the upper margins; petioles unwinged, purple. ***Scapes*** as long as or longer than leaves, sparsely hairy, especially under the capitulum, their lower parts usually purple in colour. ***Capitulum*** convex, 2.5–4.0 cm in diameter, yellow, medium dense, outer strips grey-purple; inner bracts dark grey-green, pruinose; outer bracts usually 9–12, lanceolate, usually 6–10 mm long, 2–3.5 mm broad, grey-green, with a distinct white hyaline margin (0.1–0.3 mm broad), arcuate-reflexed, without or with a small corniculum; stigmas dark, greyish-green, pollen present. ***Achenes*** red-brown, with thin and long spinules in the upper part, 3.5–4.2 mm long (incl. the 1.0–1.4 mm long, cylindrical pyramid), rostrum 7–9 mm long, pappus white.

##### Flowering period.

April–May.

##### Habitat.

Mostly sunny, termophilic-ruderal places such as roadsides, pastures, forest edges and paths. In Wielkopolska Lowland (Chwałkowo Kościelne) we noted this species at the edge of a pine-oak grove accompanied by *Adoxamoschatellina*, *Capsellabursa*-*pastoris*, *Geraniumpusillum*, *Stellariamedia*, *Taraxacumproximum*, *Veronicahederifolia* s.l. In Podlachia (Piątnica) we found this species in a pastured dry sandy grassland together with *Achilleamillefolium*, *Artemisiacampestris*, *Cerastiumsemidecandrum*, *Echiumvulgare*, *Festucarubra*, *Galiummollugo*, *Pimpinellasaxifraga*, *Plantagomedia*, *Potentillaarenaria*, *P.argentea*, *Salviaverticillata*, *Sedumacre*, *Taraxacumbellicum*, *Trifoliumrepens*.

##### Somatic chromosome number.

24 (Wolanin and Musiał 2017).

##### General distribution.

Central, Western and Northern Europe species, reported from France, Switzerland, Austria, Germany, the Netherlands, Denmark, the Czech Republic, Poland, Finland, Norway, Sweden and Hungary (Van Soest 1967, 1969; Doll 1973b; Tacik 1980; Kerguélen 1993; Lundevall and Øllgaard 1999; Uhlemann 2003; Głowacki et al. 2004; Trávníček et al. 2010; Wendt and Øllgaard 2015). This species is probably not native to Great Britain (Sell and Murrell 2006; EURO+MED 2006-onwards).

##### Distribution in Poland.

Scattered localities, rare (Fig. 10E).

##### Specimens examined.

**AB23** – Międzyzdroje, lawn, 53°56'10"N, 14°27'24"E, 30 April 2017, *M. Wolanin* (003261 UR), **BD43** – Kebłowo, ruderal area near cemetery fence, 52°03'06"N, 16°06'34"E, 19 April 2016, *M. Wolanin* (003326 UR); **CD41** – Chwałkowo Kościelne, roadside in forest, 51°59'41"N, 17°18'12"E, 16 April 2016, *M. Wolanin* (003327 UR); **CF11** – Nysa (Śląsk), May 1849, *M. Winkler* (WRSL); **DA81** – Gdańsk (Stogi), roadside in forest, 54°22'15"N, 18°43'06"E, 7 May 2016, *M. Wolanin* (003313 UR); **DC41** – Sąsieczno, pine forest edge, 52°57'03"N, 18°50'38"E; 29 April 2018, *M. Wolanin* (003471 UR); **DC52** – between Wakole and Dąbrówka, roadside in pine forest, 52°51'40"N, 18°58'09"E, 29 April 2018, *M. Wolanin* (003450 UR); **DF06** – Czarny Kamień near Moczydło, distr. Żarki, 29 May 1976, *A. Sendek* (12513 KTU); **DF45** – Sosnowiec Maczki, roadside at pine forest edge, 50°15'34"N, 19°17'07"E, 2 May 2016, *M. Wolanin* (003300 UR); **DF58** – Bolechowice, below calcareous rock, 21 May 1976, *T. Tacik* (575895 KRAM); **FC13** – Piątnica (Fort Łomża), pastured grassland, 53°11'50"N, 22°06'53"E, 25 April 2016, *M. Wolanin* (003274, 003287, 003567 UR).

##### Notes.

**Figure 19.**
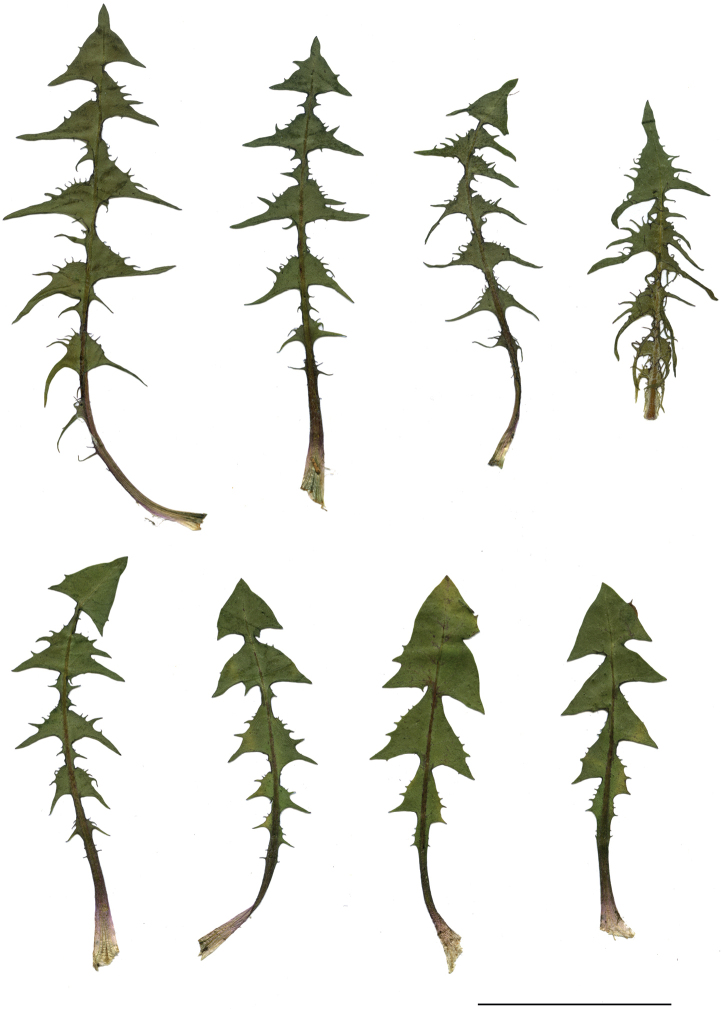
Variation in leaf shape in *T.disseminatum*; locality – Chwałkowo Kościelne (*M. Wolanin* 2016 UR). Scale bar: 5 cm.

**Figure 20.**
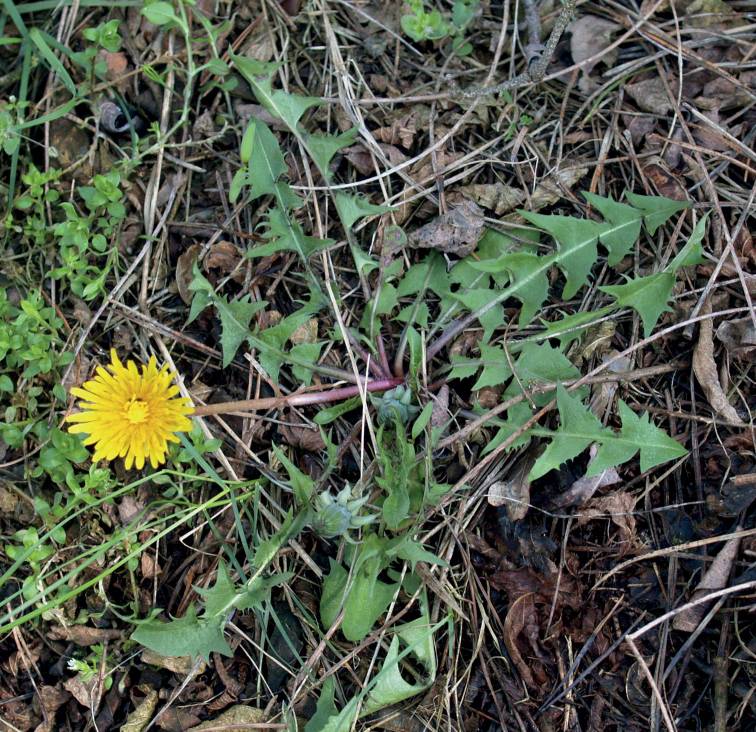
*Taraxacumdisseminatum*; locality – Chwałkowo Kościelne, 2016, photo by M. Wolanin.

Plant quite large with a medium dense capitulum (particularly visible in the peripheral part of the inflorescence) up to 4 cm in diameter. Leaves broad with a rather large triangular terminal lobe. The terminal lobe edge is strongly lobed and serrated in the base part. Outer bracts with significant wide hyaline margin. Species distinct and easy to recognise, although not very common, and usually populations are not numerous (Figs 19, 20).

#### ﻿6. Taraxacum dissimile

Taxon classificationPlantaeAsteralesAsteraceae

Dahlst., Ark. Bot. 10(11): 8. 1911.

727ECAE7-F5E9-5E2F-A2AA-51C1B47457B9

##### Type.

Sweden, Gothenburg archipelago, Branno, seashore, 19 May 1910, *Th. Lange* (lectotype in TURA [sheet No. I, middle specimen], designated by Lundevall and Øllgaard 1999: 78; isolectotype in TURA [sheets No. 2 and 3]).

##### Description.

Plants small to middle-sized, 5–12 cm tall. ***Leaves*** greyish-green, sparsely hairy on the upper side, approximately 5–10(–12) cm long and (1–)2.0–3.0(–4.0) cm wide, usually 4–5 times longer than wide, blades oblanceolate, usually broadest in upper 1/4, with 3–4 pairs of lateral lobes; lateral lobes opposite to remote; lateral lobes of the inner leaves triangular, straight or a little recurved, broad at the base, with an entire or slightly denticulate, straight or somewhat convex distal margin, proximal margin usually entire; lateral lobes of the outer leaves triangular, entire or somewhat denticulate at the distal margin; interlobes usually winged and flat, sometimes crisped, green or blackish coloured; terminal lobe distinct, triangular, often incised, entire or denticulate on the upper sides; petioles unwinged, purple. ***Scapes*** as long as or shorter than leaves, sparsely hairy below the capitulum. ***Capitulum*** convex, 3.0–3.5 cm in diameter, yellow, medium dense, outer strips greyish-red; inner bracts dark green, somewhat pruinose; outer bracts usually 12–16, lanceolate, usually 7–8 mm long, 2.5–3.5 mm broad, grey-green suffused pruinose, with a distinct white hyaline margin (0.2–0.5 mm broad), recurved and corniculate; stigmas blackish, pollen absent or very poorly developed (up to a few grains on the stigma). ***Achenes*** yellowish-greyish-brown, 3.6–4.2 mm long (incl. the 0.9–1.2 mm long, cylindrical cone), rostrum 8–9 mm long, pappus white.

##### Flowering period.

(April) May.

##### Habitat.

Species observed in dry, sandy, semiruderal places such as pastures, lawns and forest road edges. On the coast of the Baltic Sea (between Krynica Morska and Piaski), we noted this species on the forest roadside together with *Achilleamillefolium*, *Arabidopsisthaliana*, *Carexovalis*, *C.praecox*, *Cerastiumholosteoides*, *C.semidecandrum*, *Equisetumarvense*, *Erophilaverna*, *Festucarubra*, *Luzulacampestris*, *Plantagolanceolata*, *Poapratensis*, *Potentillaargentea*, *Ranunculusbulbosus*, *Veronicaarvensis*, *Vicialathyroides*.

##### Somatic chromosome number.

24 (Wolanin and Musiał 2017).

##### General distribution.

Central, North and East Europe; species reported in Germany, the Netherlands, Denmark, Poland, Norway, Sweden, Finland, Ukraine, Belarus, Lithuania, Latvia, Estonia, Central and Northwest European Russia (Van Soest 1967; Tutin et al. 1976; Fedorov 1989; Lundevall and Øllgaard 1999; Mosyakin and Fedoronchuk 1999; Øllgaard et al. 2000; Wendt and Øllgaard 2015). This species is probably not native to Belgium (Lambinon et al. 2004; EURO+MED 2006-onwards).

##### Distribution in Poland.

Scattered localities in north-eastern Poland, quite rare (Fig. 10F).

##### Specimens examined.

**CA43** – Łeba, dry lawn, 54°46'05"N, 17°35'28"E, 3 May 2019, *M. Wolanin* (003572 UR); Łeba, dune, 54°46'05"N, 17°34'05"E, 1 May 2017, *M. Wolanin* (003265, 003278 UR); **DA51** – Hel, Leśna street, lawn, 54°36'05"N, 18°49'16"E, 9 May 2016, *M. Wolanin* (003324 UR); **DA76** – Piaski, sandy roadside, 54°25'12"N, 19°34'04"E, 10 May 2016, *M. Wolanin* (003307 UR); **DA86** – between Krynica Morska and Piaski, sandy roadside in forest, 54°24'21"N, 19°31'44"E, 10 May 2016, *M. Wolanin* (003281 UR); **DC32** – vicinity of the village Golub-Dobrzyń, roadside near Okonin lake, 53°04'27"N, 18°57'40"E, 29 April 2018, *M. Wolanin* (003477 UR); **DC52** – between Wakole and Dąbrówka, roadside in pine forest, 52°51'40"N, 18°58'09"E, 29 April 2018, *M. Wolanin* (003447 UR); **FB77** – Osowiec, dry pasture close to fort, 53°29'25"N, 22°38'32"E, 24 April 2016, *M. Wolanin* (003255, 003268 UR); **FC13** – Stara Łomża, roadside, 17 May 1998, *Z. Głowacki* (527696 KRAM); **GD10** – Serpelice, lawn, 52°16'49"N, 23°03'01"E, 25 April 2016, *M. Wolanin* (003294 UR).

##### Notes.

**Figure 21.**
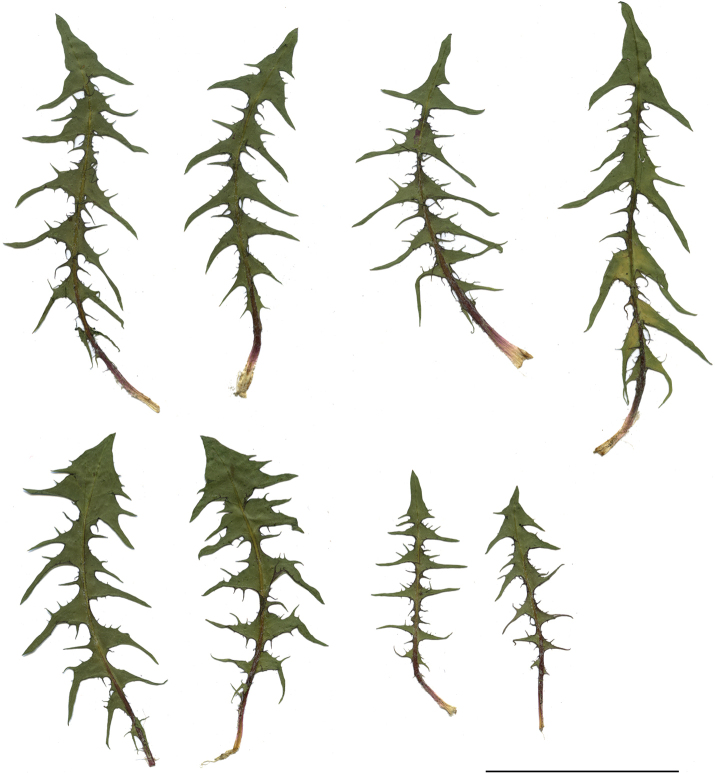
Variation in leaf shape in *T.dissimile*; locality – between Krynica Morska and Piaski (*M. Wolanin* 2016 UR). Scale bar: 5 cm.

**Figure 22.**
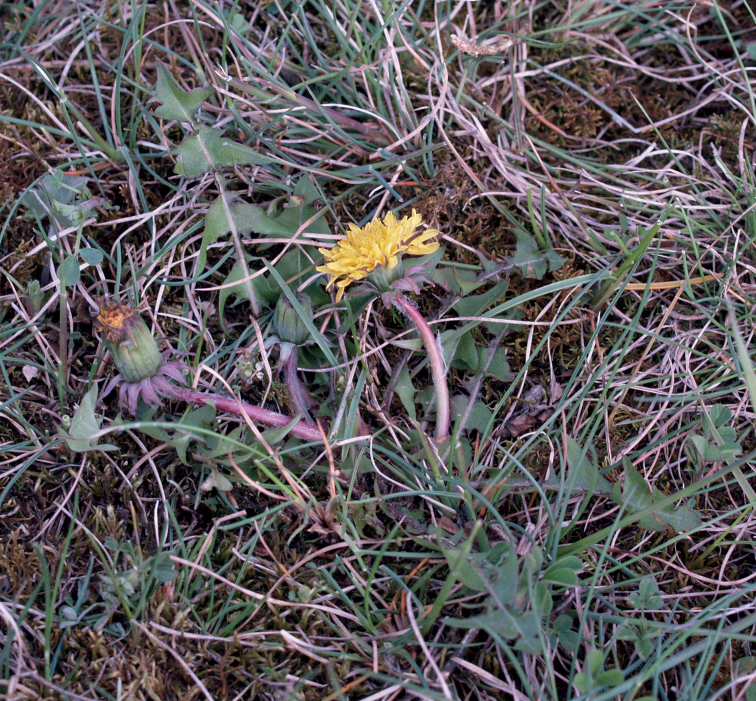
*Taraxacumdissimile*; locality – Osowiec, 2016, photo by M. Wolanin.

This species belongs to the *Dissimilia* group due to its yellowish-greyish-brown achenes, sharply outlined, triangular-sagittate and incised terminal lobe. Outer phyllaries are large, grey-green, pruinose with a wide hyaline border. The plant is dark in colour with dark stigmas and quite bright outer phyllaries, which makes it conspicuous in the field. Pollen is not present (Figs 21, 22).

#### ﻿7. Taraxacum lacistophyllum

Taxon classificationPlantaeAsteralesAsteraceae

(Dahlst.) Raunk., Dansk Exkurs.-Fl., ed. 2: 257. 1906.

0A9D5EFB-12E0-5465-8736-C2DEE281208C

Taraxacum erythrospermum subsp. lacistophyllum Dahlst., Bot. Not., 1905: 153, 168. 1905. Basionym.

##### Type.

Sweden, Ostergotland, Linkoping, Magistratshagcn, 4 June 1889, *H. Dahlstedt* (lectotype in S [bottom specimen], designated by Lundevall and Øllgaard 1999: 81; isolectotype in S).

##### Description.

Plants small to middle-sized, 10–15(–20) cm tall. ***Leaves*** greyish-green, almost glabrous or with few barely visible hairs, approximately (5–)8–12(–15) cm long and (1.5–)2.0–3.0(–4.0) cm wide, usually 3–5 times longer than wide, blades elliptical, with 4–6 pairs of lateral lobes; lateral lobes opposite to remote; lateral lobes of the inner leaves patent and falcate, with a mostly entire or (at lower-positioned lobes) slightly denticulate distal margin, proximal margin usually entire; lateral lobes of the outer leaves triangular, with a mostly entire, convex distal margin; interlobes often crisped; terminal lobe of the inner leaves tripartite, subsagittate, with a somewhat elongated apex, mostly entire at the margins; terminal lobe of the outer leaves triangular, subacute; petioles narrow, unwinged, purple. ***Scapes*** as long as or longer than leaves, somewhat hairy, especially just under the capitulum. ***Capitulum*** convex, 3.5–4.0 cm in diameter, light yellow, outer strips grey-purple; inner bracts dark, greyish-green, pruinose, corniculate; outer bracts usually 12–14, lanceolate, usually 7–9 mm long, 2–2.5 mm broad, greyish-green/violet, pruinose with a white hyaline margin (0.05–0.1 mm broad), spreading-arcuate and corniculate; stigmas greyish-green, pollen present. ***Achenes*** brown-red, with long spinules in the upper part, (3.5–)3.8–4.1(–4.3) mm long (incl. the 0.7–1.0(–1.2) mm long, cylindrical cone), rostrum 6–8 mm long, pappus white.

##### Flowering period.

April–May.

##### Habitat.

Species most often found in semiruderal locations, such as sandy and sunny edges of pine forests, paths, cliffs; less often in ruderal habitats (concrete promenades, walls). On the coast of the Baltic Sea (Gdańsk) we noted this species on the edge of a sandy forest road, accompanied by *Achilleamillefolium*, *Agrostiscapillaris*, *Alliariapetiolata*, *Anthriscussylvestris*, *Artemisiavulgaris*, *Berteroaincana*, *Hypericummaculatum*, *Melandriumalbum*, *Plantagomajor*, *Potentillaargentea*, *Tanacetumvulgare*, *Tragopogonpratensis*.

##### Somatic chromosome number.

24 (Wolanin and Musiał 2017), 25 (Małecka 1969).

##### General distribution.

Central, Western and Northern Europe. Species reported from Portugal, Spain, France, Great Britain, Ireland, Corsica, Italy, Switzerland, Belgium, the Netherlands, Denmark, Germany, the Czech Republic, Hungary, Norway, Sweden, Finland, Poland, Latvia and Lithuania (Van Soest 1957, 1967; Doll 1973b; Tacik 1980; Fedorov 1989; Richards 1992; Dudman and Richards 1997; Lundevall and Øllgaard 1999; Uhlemann 2003; Trávníček et al. 2010; Wendt and Øllgaard 2015).

##### Distribution in Poland.

**Figure 23.**
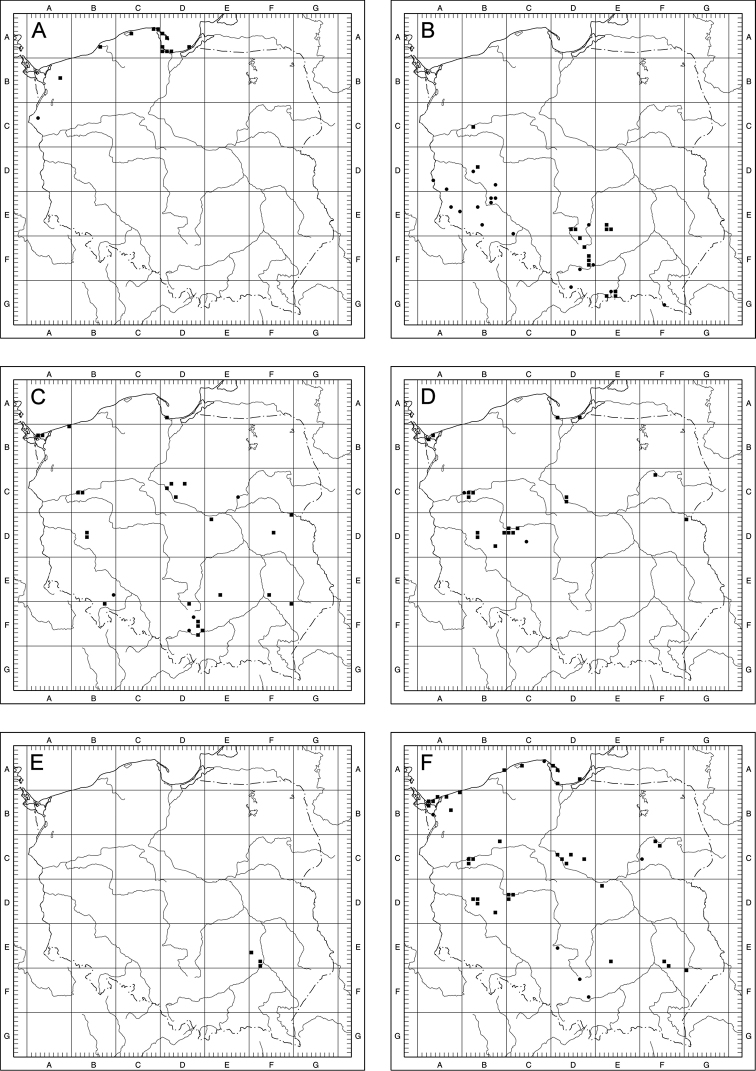
Distribution maps of Taraxacumsect.Erythrosperma in Poland **A***T.lacistophyllum***B***T.parnassicum***C***T.plumbeum***D***T.proximum***E***T.sandomiriense***F***T.scanicum*; black square – localities recorded during field studies, black circle – other localities known from herbarium data.

Species noted only in Pomerania, chiefly on the coast of the Baltic Sea (Fig. 23A).

##### Specimens examined.

**AB47** – vicinity of Unibórz, roadside ditch edge (edge of pine forest), 53°48'49"N, 15°04'53"E, 30 April 2017, *M. Wolanin* (003417 UR); **AC32** – vicinity of Chojna, roadside, 17 May 2015, *B. Kurnicki* (SZUB); **BA76** – Darłówkowo, pine forest edge, 54°26'32"N, 16°23'22"E, 1 May 2017, *M. Wolanin* (003401 UR); **CA38** – Jastrzębia Góra, lawn, 54°49'52"N, 18°17'42"E, 9 May 2016, *M. Wolanin* (003400 UR); Rozewie, sandy roadside close to lighthouse, 54°49'49"N, 18°19'57"E, 9 May 2016, *M. Wolanin* (003262 UR); 1,5 km E of Rozewie, cliff, 30 May 1969, Stasiak (152/03 UGDA); W of Chłapowska Valley outlet, loose scrubs of Hippophae, 25 June 1970, *W. Chojnacki* (153/04 UGDA); **CA39** – Władysławowo, lawn close to parking lot in forest, 54°47'19"N, 18°25'40"E, 9 May 2016, *M. Wolanin* (003301 UR); **CA43** – Łeba, dry lawn near amusement park, 54°46'05"N, 17°35'28"E, 3 May 2019, *M. Wolanin* (003574 UR); Łeba, clearing in pine forest, 54°45'49"N, 17°32'31"E, 2 May 2019, *M. Wolanin* (003577 UR); **DA40** – Jastrania, grassy path near parking lot in forest, 54°42'49"N, 18°38'15"E, 9 May 2016, *M. Wolanin* (003354 UR); **DA51** – Hel, Leśna street, sandy roadside in forest, 54°36'08"N, 18°48'55"E, 9 May 2016, *M. Wolanin* (003330 UR); Hel, Leśna street, pine scrub edge, 54°36'09"N, 18°48'49"E, 9 May 2016, *M. Wolanin* (003382 UR); Hel, meadow close to weather station, 8 May 1997, *K. Błaszkiewicz* (058185, 058190 KTU); Hel, dunes close to weather station, 8 May 1997, *H. Øllgaard* (527654 KRAM); **DA70** – Sopot, fissure in stone wall along promenade, 54°27'24"N, 18°33'44"E, 8 May 2016, *M. Wolanin* (003318 UR); Sopot, neglected lawn in park, 54°27'08"N, 18°33'49"E, 8 May 2016, *M. Wolanin* (003386 UR); **DA76** – Piaski, sandy roadside, 54°25'11"N, 19°34'00"E, 10 May 2016, *M. Wolanin* (003373 UR); **DA80** – Gdańsk (Roland pleasure ground), lawn on sandy soil, 54°24'45"N, 18°36'17"E, 8 May 2016, *M. Wolanin* (003328, 003329 UR); Gdańsk (Roland pleasure ground), lawn on sandy soil, 54°24'44"N, 18°36'28"E, 8 May 2016, *M. Wolanin* (003314 UR); Gdańsk (Westerplatte), gap in pavement, 54°24'23"N, 18°40'34"E, 7 May 2016, *M. Wolanin* (003372, 003398 UR); **DA81** – Gdańsk Stogi, along path in light pine forest, 54°22'24"N, 18°43'37"E, 7 May 2016, *M. Wolanin* (003288 UR); **DA82** – Świbno, sandy roadside in forest, 54°20'16"N, 18°56'12"E, 10 May 2016, *M. Wolanin* (003275, 003355 UR).

##### Notes.

**Figure 24.**
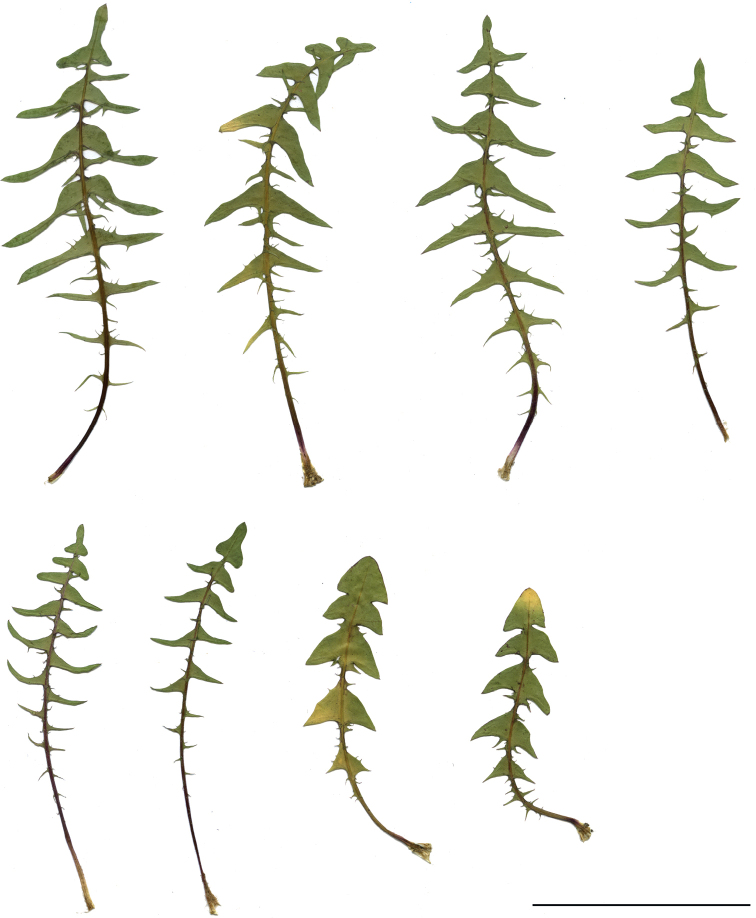
Variation in leaf shape in *T.lacistophyllum*; locality – Jastrania (*M. Wolanin* 2016 UR). Scale bar: 5 cm.

**Figure 25.**
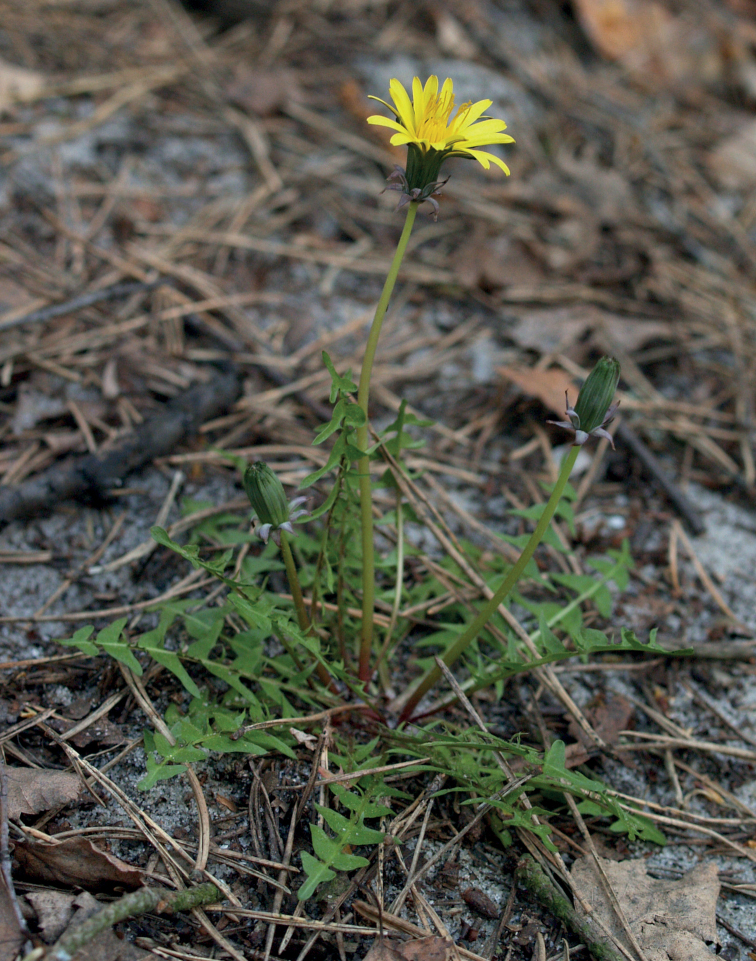
*Taraxacumlacistophyllum*; locality – Hel, 2016, photo by M. Wolanin.

Plant charming, gentle, with tasteful capitulum up to 4 cm in diameter, light yellow ligules, outer bracts spreading-arcuate, greyish-green/violet, pruinose. Leaves regularly lobed, side lobes most often falcate and interlobes often crisped. Species easy to recognise (morphological features of the leaves are highly visible, even for specimens growing in unusual places) (Figs 24, 25).

#### ﻿8. Taraxacum parnassicum

Taxon classificationPlantaeAsteralesAsteraceae

Dahlst., Acta Horti Berg. 9: 29. 1926.

3910C150-51AE-5AC4-9AAF-F04664F05FB3

= Taraxacumsilesiacum Dahlst. ex G. E. Hagl., Bot. Not. 500. 1938. Type: Poland, Slask (Silesia, Schlesien), Legnica (Liegnitz), (Callier, Fl. Sielesiaca Exsicc. No. 1224) – cult. in Sweden, Stockholm, bergian Bot. Garden 6, 3 June 1904, *H. Dahlstedt* (holotype in S; isotype in S).

##### Type.

Greece, Parnassos 1921 G. Samuelson – cult. In Hort. Bot. Upsaliensis, 22 May 1924, *G. Samuelson* (lectotype S, designated by Lundevall and Øllgaard 1999: 125; isolectotypes in S and BM).

##### Description.

Plants small, up to 5–10 cm tall. ***Leaves*** pure green, almost glabrous, approximately 3–5(–8) cm long and 1–2 cm wide, usually 3–4(–5) times longer than wide, blades oblanceolate to narrowly oblanceolate, usually broadest in upper 1/3, with 4–5(–7) pairs of lateral lobes; lateral lobes mostly opposite (to remote); lateral lobes of the inner leaves triangular, slightly recurved, with a somewhat convex, entire or barely toothed distal margin, proximal margin usually entire; lateral lobes of the outer leaves triangular, crowded, entire or with a few teeth at the distal margin; interlobes often narrow, with a solitary tooth; terminal lobe of the inner leaves tripartite, with a short subacute tip, entire on the margins; terminal lobe of the outer leaves triangular; petio­les unwinged, purple. ***Scapes*** as long as or shorter than leaves, somewhat hairy, especially under the involucres. ***Capitulum*** flat or convex, dense, 2.0–2.5 cm in diameter, light yellow, outer strips purple; inner bracts green, pruinose, corniculate; outer bracts usually 9–12, ovate to wide lanceolate, usually 5–6 mm long, 1.5–2.5 mm broad, greyish-green, suffused purple, with a white hyaline margin (0.1–0.2 mm broad), erect at the base, recurved at apex, somewhat corniculate; stigmas dark, greyish-green, pollen absent or very poorly developed (up to a few grains on the stigma). ***Achenes*** brown-red, with relatively short spinules in the upper part, (3–)3.5–4.1(–4.3) mm long (incl. the 0.7–1.1 mm long, cylindrical or slightly conical cone), rostrum 5–8.5 mm long, pappus white.

##### Flowering period.

April–May.

##### Habitat.

In the south of Poland, *T.parnassicum* usually grows in thermophilic grasslands on limestone rocks (most often in trampled or eroded places) and in rock crevices. In the north, this species was recorded in sandy grasslands and on a dry lawn. Plant communities with the participation of *T.parnassicum* are dominated by species characteristic to the *Festuco-Brometea*, *Molinio-Arrhenatheretea*, *Sedo-Scleranthetea* and *Trifolio-Geranietea sanguinei* classes. In Kraków-Częstochowa Upland (Kusięta) we observed this species in a grassland on limestone rocks together with *Alyssumalyssoides*, *Arenariaserpyllifolia*, *Arrhenatherumelatius*, *Artemisiacampestris*, *Asperulacynanchica*, *Carexcaryophyllea*, *Centaureastoebe*, *Cerastiumarvense*, *C.semidecandrum*, *Convolvulusarvensis*, *Coronillavaria*, *Erophilaverna*, *Euphorbiacyparissias*, *Festucarubra*, Helianthemumnummulariumsubsp.obscurum, *Hypericummaculatum*, *Medicagofalcata*, *Papaverargemone*, *Phleumphleoides*, *Plantagolanceolata*, *P.media*, *Poapratensis*, *Potentillaarenaria*, *Ranunculusbulbosus*, *Sanguisorbaminor*, *Silenenutans*, *S.vulgaris*, *Stachysrecta*, *Teucriumbotrys*, *Thymuspulegioides*, *Trifoliummontanum*, *Veronicaarvensis*, *V.spicata*, *Vincetoxicumhirundinaria*. In Pieniny Mts (Jaworki) we noted this species in a pastured rock grassland accompanied by *Acinosarvensis*, *Agrostiscapillaris*, *Alchemillaglaucescens*, *Anthyllisvulneraria*, *Arabishirsuta*, *Arenariaserpyllifolia*, *Botrychiumlunaria*, *Brizamedia*, *Bupleurumfalcatum*, *Calamagrostisvaria*, *Carexflacca*, *C.transsilvanica*, *Cerastiumholosteoides*, *Cotoneasterintegerrimus*, *Cruciataglabra*, *Euphorbiacyparissias*, *Festucapallens*, *F.pratensis*, *Fragariavesca*, *Galiummollugo*, *Hypericumperforatum*, *Jovibarbasobolifera*, *Leontodonhispidus*, *Leucanthemumvulgare*, *Medicagofalcata*, *M.lupulina*, *Phleumpratense*, *Pilosellaofficinarum*, *Plantagomedia*, *Polygalacomosa*, *Potentillaneumanniana*, *Ranunculuspolyanthemos*, *Salviaverticillata*, *Sanguisorbaminor*, *Sedumacre*, *Silenenutans*, *Thymuspulegioides*, *Verbascumnigrum*.

##### Somatic chromosome number.

24 (Wolanin and Musiał 2017), 26 (Małecka 1969).

##### General distribution.

Widespread European species reported in France, Ireland, Great Britain, Corsica, Italy, Switzerland, Belgium, the Netherlands, Germany, Denmark, Austria, Germany, the Czech Republic, Poland, Slovakia, Hungary, Ukraine, Romania, Bulgaria, Montenegro, Croatia, Greece and Macedonia. In Sweden, this plant is a naturalised alien (Rohlena 1942; Van Soest 1967; Doll 1973b; Tacik 1980; Gamisans 1985; Dostál 1989; Mosyakin and Fedoronchuk 1999; Uhlemann 2003; Conti et al. 2005; Trávníček et al. 2010; Dimopoulos et al. 2013; Wendt and Øllgaard 2015).

##### Distribution in Poland.

Scattered localities in southern and western Poland, frequent only in the western part of Lesser Poland (Fig. 23B).

##### Specimens examined.

**AD73** – Zasieki distr. Lubsko, roadside, 28 July 1972, *E. Kozioł* (32319 WRSL); **AD96** – Iłowa Żagańska distr. Żary, sandy roadside, 10 June 1971, *E. Kozioł* (34605 WRSL); **AE37** – Nowogrodziec distr. Bolesławiec, railway embankment close to cement plant, 18 May 1972, *E. Kozioł* (WRSL 32322); Nowogrodziec distr. Bolesławiec, slope of railway embankment E of city, 18 May 1972, *E. Kozioł* (34556 WRSL); **AE49** – Lwówek Śl., sunny hill W of city, 3 May 1972 *E. Kozioł* (32323 WRSL); Lwówek Śląski, sunny hill, opposite school, 1 km W of city, 30 April 1972, *E. Kozioł* (35454 WRSL); **BC52** – Stare Bielice, sandy roadside of asphalt road, 52°50'57"N, 15°55'04"E, 18 April 2016, *M. Wolanin* (003322 UR); **BD43** – Kębłowo, cemetery lawn, 52°03'04"N, 16°06'41"E, 19 April 2016, *M. Wolanin* (003517 UR); **BD52** – Sterlicz near Sława Śl., sandy roadside, 10 June 1976, *E. Kozioł* (WRSL); **BD87** – Bojanowo, green square, 10 June 1893, *C. Scholz* (WRSL); **BE16** – Gródek. Distr. Wołów, sandy hillock, 26 April 1972, *E. Kozioł* (211472 KRAM); **BE17** – Gródek near Strupina, sandy hillock, 3 May 1972, *E. Kozioł* (32321 WRSL); **BE26** – Grotki distr. Wołów, hillock on eastern edge of village, 15 May 1965, *Z. Głowacki* (31773 WRSL); **BE33** – Legnica, tournament place, 24 May 1895, *Callier* (WRSL); **BE74** – Świebodzice, 12 May 1955, (...) (281375 KRAM); **CE91** – Grodków, gravel pit, 3 May 1972, *E. Kozioł* (0388441, 0388442 KRA); **DE78** – Maluszyn, dry roadside, 27 April 2010, *M. Bielecki* (0396279 KRA); **DE84** – Kusięta, grassland on rock, 50°46'06"N, 19°16'16"E, 13 April 2014, *M. Wolanin* (003347 UR); Olsztyn, Góra Zamkowa, grassland on rock outcrop (NW slope), 50°44'55"N, 19°16'30"E, 13 April 2014, *M. Wolanin* (003297 UR); Olsztyn, grassland on rock, 50°44'55"N, 19°16'36"E, 12 April 2016, *M. Wolanin* (003487 UR); **DE85** – between Olsztyn and Przymiłowice, grassland on rock (SW exposure), 50°45'10"N, 19°17'05"E, 13 April 2014, *M. Wolanin* (003348 UR); **DF06** – Kroczyce, path on calcareous rocks, 50°34'20"N, 19°31'49"E, 12 April 2016, *M. Wolanin* (003502 UR); Kroczyce, path on SW slope, 50°34'20"N, 19°31'48"E, 1 May 2013, *M. Wolanin* (003277 UR); Mirów, grassland on rocks overgrown by shrubs, 50°36'53"N, 19°28'51"E, 1 May 2021, *M. Wolanin* (003597 UR); **DF27** – Żelazko, grassland on rock, SW slope, 50°25'14"N, 19°34'25"E, 12 April 2014, *M. Wolanin* (003349 UR); **DF48** – Gotkowice, xerothermic grassland, SW slope, 50°13'39"N, 19°43'31"E, 30 April 2013, *M. Wolanin* (003379 UR); Jerzmanowice, fissures on top of rock, 50°12'36"N, 19°45'19"E, 30 April 2013, *M. Wolanin* (003387 UR); Sąspów near Ojców, fissure in rock, near school, 50°13'21"N, 19°46'17"E, 21 April 2015, *M. Wolanin* (003310 UR); between Ojców and Grodzisko, calcareous rocks, 26 May 1929, *B. Pawłowski* (117613 KRA); between Ojców and Grodzisko, grassy slope, 26 May 1929, *B. Pawłowski* (189646 KRAM); Ojców, 27 July 1929, K. Piech (169652 KRAM); **DF58** – Bębło, grassland at the base of rock on SW slope, 50°10'51"N, 19°47'18"E, 30 April 2013, *M. Wolanin* (003406 UR); Duże Skałki, rocky paths, fissures in rocks, 50°11'20"N, 19°48'23"E, 30 April 2013, *M. Wolanin* (003414 UR); Słoneczne Skały, rock crumbs, SW rock side, 50°12'09"N, 19°45'51"E, 30 April 2013, *M. Wolanin* (003258 UR); Bolechowice, rocks in Bolechowice Ravine, 6 May 1948, *H. Błaszczyk* (113891 KRA); Bolechowice, path, 21 May 1975, *T. Tacik* (392462 KRAM); Bolechowice Valley, 6 May 1948, *Pogan* (0238571 KRA); **DF68** – Nielepice, grassland on S slope of calcareous rock, 50°06'13"N, 19°43'07"E, 12 April 2014, *M. Wolanin* (003271 UR); Bielany near Kraków, 24 April 1954, *Turnau* (0155143 KRA); between Kryspinów and Bielany, limestone hillock, 16 May 1976, *H.*, *T.* & *J. Tacik* (387574 KRAM); Skała Kmity, fissure in rock, 24 April 1991, *A. Woszczenko* (403319 KRAM); **DF69** – Las Wolski near Kraków, 24 April 1954, *Gromczakiewicz* (0155142 KRA); **DF76** – Grochowiec near Ryczów, 22 May 1926, *A. Kozłowska* (242483 KRAM); **DG14** – Grojec near Żywiec, grassland, 9 June 2000, *K. Nowak* (562018 KRAM); **EE72** – Miedzianka Hill near Chęciny, grassland on SE rock slope, 50°50'47"N, 20°21'37"E, 11 April 2016, *M. Wolanin* (003511 UR); **EE82** – Grząby Bolmińskie, field road, 50°48'46"N, 20°21'44"E, 22 April 2016, *M. Wolanin* (003321, 003388 UR); **EE83** – Sosnówka Hill, fissures in calcareous rocks, 50°48'32"N, 20°25'54"E, 18 April 2012, *M. Wolanin* (003405 UR); **EG23** – Łącko, dry grassland close to Dunajec River, 3 May 1970, *K. Towpasz* (80794 KRA); **EG24** – Czerszlowe Skałki (Pieniny Mts.), grassland on SW rock slope, 49°32'54"N, 20°32'34"E, 10 April 2014, *M. Wolanin* (003368 UR); **EG32** – “Pod Kirą” (Pieniny National Park), rock near road from Czorsztyn to Sromowce, 49°25'04"N, 20°20'36"E, 9 April 2014, *M. Wolanin* (003367 UR); Wżar Hill (Gorce Mts.), grassland on rock, E side, 22 April 1966, *H. Trzcińska-Tacik* (092621 KRAM); Wżar, rocks, 22 April 1966, *A. Zielińska* (063790 KRA); **EG34** – Jaworki, grassland on SE rock slope, 49°24'19"N, 20°32'37"E, 10 April 2014, *M. Wolanin* (003284 UR); **FG55** – Łupków, dry slope at altitude of 610 m asl, 2 May 1961, *A. Jasiewicz* (018948, 437761 KRAM).

##### Notes.

**Figure 26.**
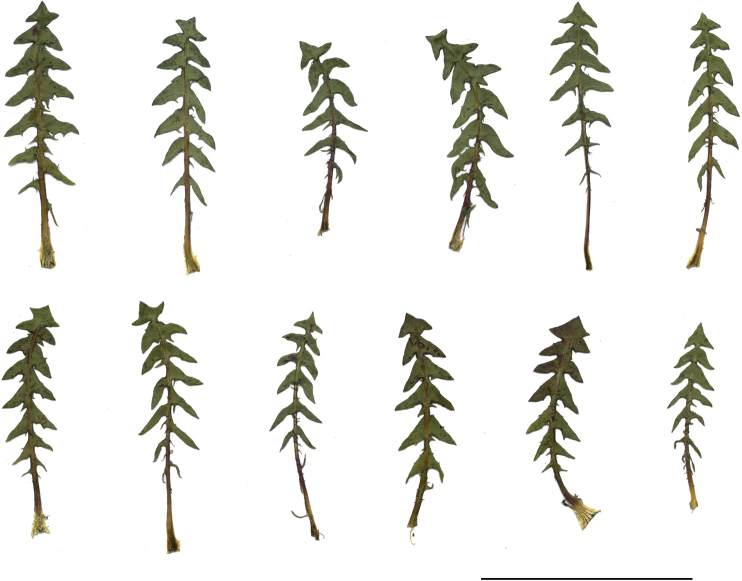
Variation in leaf shape in *T.parnassicum*; locality – Duże Skałki (*M. Wolanin* 2016 UR). Scale bar: 5 cm.

**Figure 27.**
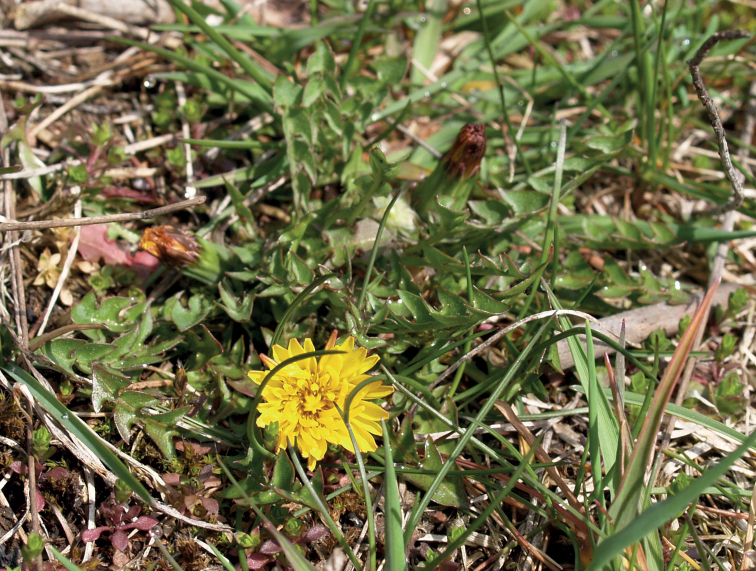
*Taraxacumparnassicum*; locality – Jaworki, 2014, photo by M. Wolanin.

Plant usually small. Leaves with 4–7 pairs of uniform lateral lobes and narrow interlobes, side lobe distal margin often convex and entire. Capitulum small, with light yellow ligules, no pollen or only rarely a few poorly developed grains present. Fruit with relatively short spinules. Species not very morphologically variable, easy to recognise, charming (Figs 26, 27).

#### ﻿9. Taraxacum plumbeum

Taxon classificationPlantaeAsteralesAsteraceae

Dahlst. Ark. Bot. 10(6): 2. 1911.

8320BBB5-1D00-54A3-B159-178ED0CB060F

= Taraxacumfranconicum Sahlin, Ber. Bayer. Bot. Ges. 55: 49. 1984. Type: Germany, Bayern, Südosthang des Hessekberges, Gem. Röckingen, Verbandsgemeinde Hesselberg, Krs. Ansbach, (MTB 6929/1), mit Malmschotter befestigter Weg im Opalinuston, June 1983, *E. J. Krach* (holotype in M 0152765; isotype in S 05-8711).

##### Type.

Sweden, Gotland, Visby, the old harbour, 6 June 1909, *H. Dahlstedt* (lectotype in S, designated by Doll 1973: 123; isolectotypes in S).

##### Description.

Plants small to middle-sized, 5–10(–15) cm tall. ***Leaves*** dark green, dull, sparsely hairy, approximately 5–12 cm long and 1.5–2.5(–3.0) cm wide, usually 5–7 times longer than wide, blades narrowly elliptical to narrowly oblanceolate, with 4–6 pairs of lateral lobes; lateral lobes opposite to remote; lateral lobes of the inner leaves narrowly triangular, usually falcate, acute, with a somewhat convex, often denticulate distal margin, proximal margin usually entire, concave; lateral lobes of the outer leaves triangular, distinctly falcate, with an entire or denticulate distal margin; interlobes often long and narrow, plicate and denticulate, blackish rimmed; terminal lobe of the inner leaves with lingulate apex, denticulate margins and/or incised at the base; terminal lobe of the outer leaves often small, triangular/subsagitate, quite often with short, subacute apical lobule; petioles unwinged or narrowly winged, purple, hairy at base. ***Scapes*** usually as long as leaves and hairy. ***Capitulum*** convex, 2.5–3.0 cm in diameter, yellow, outer strips grey-purple; inner bracts glaucous greyish-green, pruinose; outer bracts usually 9–12, ovate to lanceolate, usually 6–7(–8) mm long, 2.0–2.5(–3.0) mm broad, greyish-green, suffused red-purple at the apex, with a white hyaline margin, (0.5)–0.1(–0.2) mm broad, erect to subspreading, corniculate; stigmas olive-green, pollen present. ***Achenes*** yellowish light red-brown, often with relatively short spinules in the upper part, 3–3.6 mm long (incl. the 0.6–0.8 mm long, subconical cone), rostrum 8–9 mm long, pappus white.

##### Flowering period.

April–May.

##### Habitat.

Rocky grasslands (in trampled or eroded areas), dry sandy roadsides. In Kraków-Częstochowa Upland (Kraków Kostrze place) *T.plumbeum* grew on a rocky dry roadside together with *Arenariaserpyllifolia*, *Brizamedia*, *Bromushordeaceus*, *Dianthusdeltoides*, *D.carthusianorum*, *Euphorbiacyparissias*, *Festucapratensis*, *Fragariavesca*, *Medicagofalcata*, *M.lupulina*, *Plantagolanceolata*, *P.major*, *Poapratensis*, *Potentillaarenaria*, *Trifoliumrepens*, *Veronciaarvensis*. In Wielkopolska Lowland (Stare Bielice place) we noticed this species on the sandy roadside, accompanied by *Achilleamillefolium*, *Artemisiacampestris*, *Cerastiumsemidecandrum*, *Elymusrepens*, *Festucarubra*, *Lamiumpurpureum*, *Medicagofalcata*, *Plantagolanceolata*, *P.major*, *Potentillaargentea*, *Rosacanina*, *Rumexacetosa*, *Sedumsexangulare*, *Taraxacumproximum*, *Tragopogonpratensis*.

##### Somatic chromosome number.

24 (Wolanin and Musiał 2017).

##### General distribution.

Switzerland, Italy, Austria, Germany, the Czech Republic, Poland, Ukraine, Slovakia, Denmark (Van Soest 1967; Tacik 1980; Lundevall and Øllgaard 1999; Uhlemann 2003; Vašut 2003; Conti et al. 2005; Trávníček et al. 2010; Nobis et al. 2020b).

##### Distribution in Poland.

Scattered localities in northern and southern Poland, moderately frequent (Fig. 23C).

##### Specimens examined.

**AB09** – Dźwirzyno, lawn on sandy soil, 54°09'45"N, 15°25'59"E, 2 May 2017, *M. Wolanin* (003358, 003369 UR); **AB22** – Warszów, ditch at forest edge, 53°53'40"N, 14°18'54"E, 30 April 2017, *M. Wolanin* (003334, 003350 UR); **AB23** – Międzyzdroje, klomb, 53°56'04"N, 14°27'13"E, 30 April 2017, *M. Wolanin* (003333, 003335 UR); **BC51** – Drezdenko, lawn close to cemetery fence, 52°50'06"N, 15°49'19"E, 18 April 2016, *M. Wolanin* (003285 UR); **BC52** – Stare Bielice, sandy roadside, 52°50'57"N, 15°55'04"E, 18 April 2016, *M. Wolanin* (003298 UR); **BD43** – Kębłowo, lawn in cemetery, 52°03'04"N, 16°06'40"E, 19 April 2016, *M. Wolanin* (003380 UR); between Kębłowo and Świętno, dry field road side, 52°02'34"N, 16°05'24"E, 19 April 2016, *M. Wolanin* (003259 UR); **BD53** – Kaszczor, lawn in cemetery, 51°57'19"N, 16°10'01"E, 19 April 2016, *M. Wolanin* (003563 UR); **BE89** – Strzelin: (...) near Wyszonowice, 23 May 1942, *E. Schalow* (WRSL); **BF07** – Stolec distr. Ząbkowice Śląskie, paths near calcareous rock, 50°35'49"N, 16°52'26"E, 18 April 2017, *M. Wolanin* (003443 UR); Stolec distr. Ząbkowice Śląskie, limestone hill near Stolec, roadside, 04 June 1972, *E. Kozioł* (34508 WRSL); **DC32** – vicinity of Golub-Dobrzyń, roadside close to Okonin Lake, 53°04'27"N, 18°57'40"E, 29 April 2018, *M. Wolanin* (003476 UR); **DC35** – Rypin (Bukowa street 7), lawn, 53°04'17"N, 19°25'11"E, 28 April 2018, *M. Wolanin* (003452 UR); **DC41** – Sąsieczno, pine forest edge, 52°57'03"N, 18°50'38"E; 29 April 2018, *M. Wolanin* (003471 UR); **DC63** – Winduga near Włocławek, roadside in pine forest, 52°43'18"N, 19°01'17"E, 29 April 2018, *M. Wolanin* (003459 UR); **DF06** – Kroczyce, path on calcareous rock, 50°34'20"N, 19°31'49"E, 12 April 2016, *M. Wolanin* (003502 UR); Rzędkowice, path on S slope of calcareous rock, 50°34'31"N, 19°29'07"E, 14 April 2014, *M. Wolanin* (003403 UR); Mirów, grassland on rocks overgrown by shrubs, 50°36'53"N, 19°28'51"E, 1 May 2021, *M. Wolanin* (003596 UR); **DF37** – Klucze, sandy roadside, 15 June 1953, *T. Tacik* (570166 KRAM); **DF48** – Sąspów near Ojców, sunny W slope near church, 50°13'24"N, 19°46'19"E, 21 April 2015, *M. Wolanin* (003404 UR); **DF58** – Słoneczne Skały, rock crumbs at rock base, on SW side of rock, 50°12'09"N, 19°45'51"E, 30 April 2013, *M. Wolanin* (003258 UR); Bolechowice, calcareous rocks, 1 May 1976, *H.*, *T.* & *J. Tacik* (388114 KRAM); Dolina Kluczwody, rocky cliff, 24 April 1977, *H. Trzcińska-Tacik* (388109, 575842 KRAM); Ojców, calcareous rocks near Ciemna Cave (near the path), 3 May 1952, *A. Jasiewicz* (439041 KRAM); **DF66** – Wygiełzów near Chrzanów, grassland near military bunker, 19 May 1975, *T. Tacik* (387559 KRAM); **DF69** – Kraków (Kostrze), roadside, 50°02'08"N, 19°51'58"E, 12 April 2016, *M. Wolanin* (003498 UR); Pychowicka Górka, grassland on rocky-humus soil, 50°01'53"N, 19°52'48"E, 29 April 2013, *M. Wolanin* (003445 UR); Pychowicka Górka, grassland on rock, 50°01'50"N, 19°53'00"E, 29 April 2013, *M. Wolanin* (003464 UR); Pychowice, sunny hillock, 11 May 1975, *T. Tacik* (392447 KRAM); **DF78** – Tyniec Podgórki (Góra Wielkanoc), path on rocky-humus soil, 50°01'02"N, 19°48'57"E, 29 April 2013, *M. Wolanin* (003320 UR); Skawina, sandy square near road, 5 May 1976, *H. Trzecińska-Tacik* (388116 KRAM); **EC67** – Pawłówek near Pułtusk, grassland, 8 May 2015, *J. Marciniuk*, *P. Marciniuk* (Herb. J&P Marciniuk); Szygłówek near Pułtusk, grassland near pine forest, 8 May 2015, *J. Marciniuk*, *P. Marciniuk* (Herb. J&P Marciniuk); **ED11** – Młodzieszyn, pine forest edge, 52°19'00"N, 20°12'23"E, 28 April 2018, *M. Wolanin* (003456 UR); **EE83** – Góra Zalejowa, fissure in rock, 50°49'07"N, 20°27'30"E, 20 April 2016, *M. Wolanin* (003415 UR); **FD09** – Bużka, sandy roadside, 52°21'27"N, 22°53'56"E, 25 April 2016, *M. Wolanin* (003272 UR); Kózki, pastured grassland near Kózki reserve, 52°21'39"N, 22°52'10"E, 25 April 2016, *M. Wolanin* (003311 UR); **FD45** – between Biardy and Grezówka-Kolonia, field road hardened with crushed concrete, 52°00'36"N, 22°18'33"E, 27 April 2016, *M. Wolanin* (003533 UR); **FE84** – between Zaklików and Lipa, sandy square at pine forest edge, 50°42'34"N, 22°04'39"E, 19 April 2019, *M. Wolanin* (003583 UR); **FF09** – Wola Mała, sandy square at pine forest edge, 50°32'50"N, 22°45'48"E, 13 April 2019, *M. Wolanin* (003581 UR).

##### Notes.

**Figure 28.**
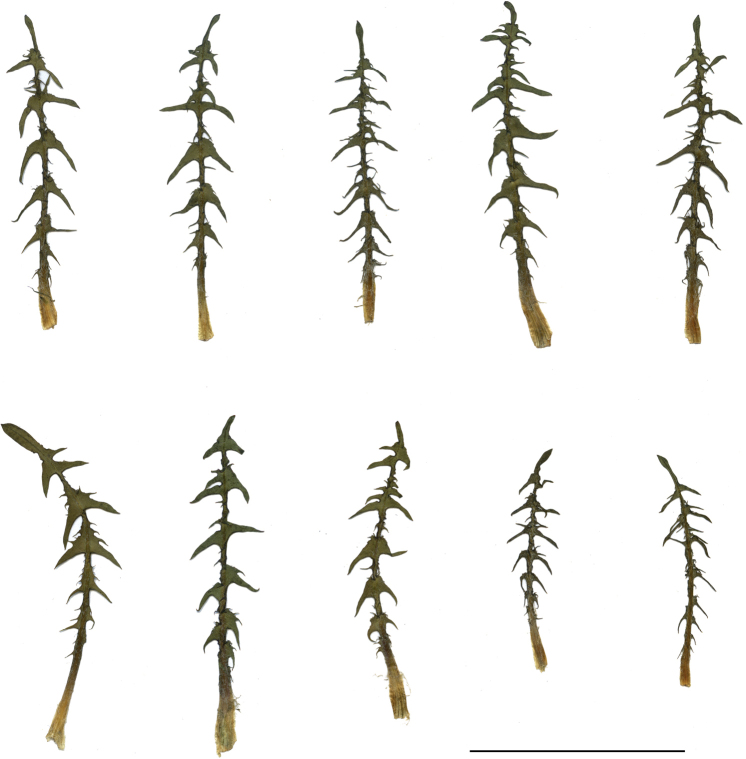
Variation in leaf shape in *T.plumbeum*; locality – Kraków Kostrze (*M. Wolanin* 2016 UR). Scale bar: 5 cm.

**Figure 29.**
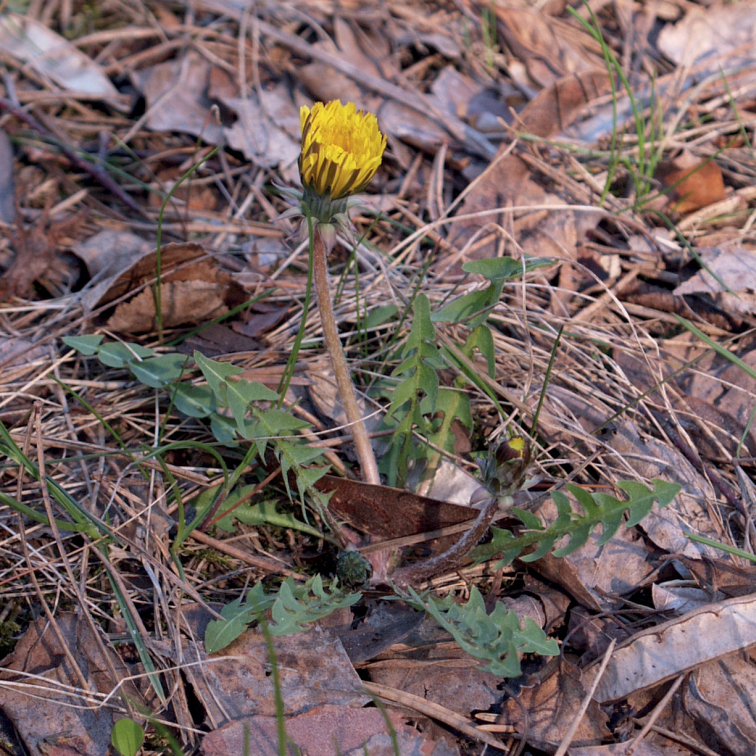
*Taraxacumplumbeum*; locality – between Zaklików and Lipa, 2019, photo by M. Wolanin.

Plant very variable in morphology, which can cause problems in determination where achenes are absent. In the Polish lowlands, we noted this species mainly in dry and warm semi-ruderal habitats (usually on sandy soils), and the specimens were relatively homogeneous in their morphological features, such as: leaves with quite wide, entire or slightly toothed side lobes and suberect or patent outer phyllaries. In upland areas, in rock grasslands, the species show greater variability of morphology compared to lowland populations. In general, side lobes are narrower, more numerous and slightly more serrated, interlobia are wider and incised, and outer phyllaries are narrower and more recurved. Specimens growing in extremely dry and rocky habitats, usually with strongly dissected leaves, may resemble *T.tenuilobum*, however the terminal lobe in *T.plumbeum* has a slightly different shape, with a tongue-shaped apex slightly incised on both sides in the base, and much smaller teeth and lobules in the interlobes in relation to the side lobes. The yellowish light red-brown hue of the achenes is a very useful diagnostic feature typical of *T.plumbeum* (Figs 28, 29).

#### ﻿10. Taraxacum proximum

Taxon classificationPlantaeAsteralesAsteraceae

(Dahlst.) Raunk., Dansk Exkurs.-Fl., ed. 2: 258. 1906.

7B2554B6-1476-5628-9621-350EEBF96BAD

Taraxacum erythrospermum subsp. proximum Dahlst., Bot. Not. 1905: 165. 1905. Basionym.
= Taraxacumattenuatum Brenner, Meddeland. Soc. Fauna Fl. Fenn. 32: 114. 1906. Type: Finland, Nylandia, Ingå (Inkoo), Svartbäck, dry hill, 17 August 1905, *M. Brenner* (lectotype in H 660607, designated by Lundevall and Øllgaard 1999: 60).

##### Type.

Sweden, Stockholm, Djurgårdsfrescati, under oaks, 5 June 1903, *H. Dahlstedt* (lectotype in S [lower plant], selected G. Haglund and designated by Doll 1973: 74).

##### Description.

Plants small to middle-sized, 10–20(–30) cm tall. ***Leaves*** greyish-green, almost glabrous, approximately 7–15(–25) cm long and 2–4 cm wide, usually 3–4 times longer than wide; leaf blade elliptical, regularly lobate, with 4–8 pairs of lateral lobes; lateral lobes opposite to remote; lateral lobes of the inner leaves triangular, acute, patent, with a regularly dentate, slightly convex distal margin, proximal margin usually entire; lateral lobes of the outer leaves triangular, usually toothed at the distal margin; interlobes often short, blade often toothed in lower part of leaf; terminal lobe subacute or subsaginate, quite often with elongate apex; petioles unwinged, purplish. ***Scapes*** as long as or longer than leaves, somewhat hairy. ***Capitulum*** convex, 2.5–3.0 cm in diameter, greenish-yellow, with numerous tubular inner flowers, outer strips purple-brownish; inner bracts greyish-green, with lumps or small cornicules; outer bracts usually 11–14, lanceolate, usually 7–9 mm long, 2–3.0 mm broad, bright greyish-green suffused with purple, narrowly bordered (up to 0.05 broad), recurved, with lumps or small cornicules; stigmas greyish-green, pollen absent or very poorly developed (up to a few grains on the stigma). ***Achenes*** reddish-brown, narrow, with erect thin spinules in the upper part, 3.5–4.1(–4.5) mm long (incl. the 0.8–1.1(–1.4) mm long, cylindrical cone), rostrum (6–)7–8(–8.5) mm long, pappus white.

##### Flowering period.

April–May.

##### Habitat.

Sandy grasslands, dry sandy roadsides, lawns. In plant communities with the participation of *T.proximum*, we reported a significant presence of species characteristic to the *Molinio-Arrhenatheretea* and *Sedo-Scleranthetea* classes. Moreover, species characteristic to the class *Galio-Urticenea* and antropophytes appeared quite often. In Wielkopolska Lowland (Olsza place) we noted this species growing on sandy field roads together with *Achilleamillefolium*, *Anthemisarvensis*, *Calamagrostisepigejos*, *Capsellabursa-pastoris*, *Cerastiumholosteoides*, *Erophilaverna*, *Festucarubra*, *Plantagomajor*, *Potentillaargentea*, *Stellariamedia*, Taraxacumsect.Taraxacum, *Trifoliumrepens*, *Veronicahederifolia* s.l., *Violaarvensis*.

##### Somatic chromosome number.

24 (Wolanin and Musiał 2017).

##### General distribution.

Widespread European species reported from France, Great Britain, Iceland, Switzerland, Belgium, the Netherlands, Austria, Germany, Denmark, the Czech Republic, Poland, Hungary, Slovakia, Moldova, Bulgaria, Ukraine, Norway, Finland, Sweden, Lithuania, Latvia, Estonia and European Russia (Van Soest 1957, 1967; Doll 1973b; Tacik 1980; Dostál 1989; Fedorov 1989; Lundevall and Øllgaard 1999; Mosyakin and Fedoronchuk 1999; Uhlemann 2003; Vašut 2003; Trávníček et al. 2010; Wendt and Øllgaard 2015).

##### Distribution in Poland.

Scattered localities in northern Poland, quite frequent in Greater Poland (Fig. 23D).

##### Specimens examined.

**AB23** – Międzyzdroje, lawn on sandy soil, 53°56'15"N, 14°27'30"E, 30 April 2017, *M. Wolanin* (003267 UR); **AB32** – W of Przybór, parking lot in forest, 53°52'39"N, 14°18'37"E, 30 April 2017, *M. Wolanin* (003254 UR); **BC50** – Gościm, cemetery, 6 May 2013, *A. Czarna* (POZNB); **BC51** – Drezdenko, lawn near cemetery, 52°50'12"N, 15°49'31"E, 18 April 2016, *M. Wolanin* (003363 UR); Drezdenko, lawn, 52°50'11"N, 15°49'19"E, 18 April 2016, *M. Wolanin* (003362 UR); Drezdenko, neglected place near cemetery fence, 52°50'06"N, 15°49'19"E, 18 April 2016, *M. Wolanin* (003392 UR); **BC52** – Chełst, lawn in cemetery, 52°49'27"N, 15°57'35"E, 18 April 2016, *M. Wolanin* (003420 UR); Stare Bielice, sandy roadside near asphalt road, 52°50'57"N, 15°55'04"E, 18 April 2016, *M. Wolanin* (003393 UR); Kwiejce, old cemetery in forest, 6 May 2013, *A. Czarna* (POZNB); **BC61** – Sowia Góra, young pine forest edge, 52°42'01"N, 15°50'42"E, 18 April 2016, *M. Wolanin* (003551 UR); **BD43** – Kębłowo, ruderal area near cemetery fence, 52°03'06"N, 16°06'35"E, 19 April 2016, *M. Wolanin* (003394 UR); **BD49** – Olsza, sandy field road, 52°04'21"N, 17°05'54"E, 17 April 2016, *M. Wolanin* (003503 UR); **BD53** – Kaszczor, lawn in cemetery, 51°57'19"N, 16°10'01"E, 19 April 2016, *M. Wolanin* (003364, 003563 UR); **BD77** – Bojanowo, Półwiejska street 12, gap between pavement and kerb, 51°42'36"N, 16°44'53"E, 19 April 2016, *M. Wolanin* (003516 UR); **CD30** – Majdany, sandy roadside in pine forest, 52°08'02"N, 17°10'58"E, 17 April 2016, *M. Wolanin* (003504 UR); Zaniemyśl, cemetery lawn, 52°09'01"N, 17°10'09"E, 17 April 2016, *M. Wolanin* (003505 UR); **CD32** – Nowe Miasto nad Wartą, cemetery lawn, 52°05'14"N, 17°23'57"E, 17 April 2016, *M. Wolanin* (003549 UR); **CD40** – Błażejewo, parking lot hardened with slag close to cemetery, 52°00'01"N, 17°08'41"E, 18 April 2016, *M. Wolanin* (003365 UR); Jarosławki, sandy place at forest edge, 52°03'09"N, 17°10'17"E, 16 April 2016, *M. Wolanin* (003411 UR); Kiełczynek, sandy roadside, 52°04'16"N, 17°12'43"E, *M. Wolanin* (003412 UR); Kiełczynek, lawn, 52°03'57"N, 17°13'57"E, 16 April 2016, *M. Wolanin* (003341 UR); Książ Wielkopolski, roadside in forest, 52°04'02"N, 17°14'47"E, 16 April 2016, *M. Wolanin* (003513 UR); Książ Wielkopolski, sandy grassland in cemetery, 52°03'55"N, 17°14'10"E, 16 April 2016, *M. Wolanin* (003280, 003306 UR); **CD41** – Chwałkowo Kościelne, roadside in forest, 51°59'41"N, 17°18'12"E, 16 April 2016, *M. Wolanin* (003410, 003512 UR); between Radoszkowo and Chromiec, sandy embankment near closed railway track, 52°02'20"N, 17°16'30"E, 16 April 2016, *M. Wolanin* (003343 UR); Rado­szkowo, young pine forest edge, 52°02'18"N, 17°16'06"E, 16 April 2016, *M. Wolanin* (003293 UR); **CD64** – Taczanowski Forest near Ostrów Wielkopolski, 11 May 2012, *A. Czarna* (POZNB); **DA81** – Gdańsk (Stogi), scrub edge, 54°22'24"N, 18°43'41"E, 7 May 2016, *M. Wolanin* (003376, 003391 UR); Krynica Morska, sandy roadside, 54°23'35"N, 19°28'46"E, 10 May 2016, *M. Wolanin* (003375 UR); **DC63** – Winduga near Włocławek, roadside in pine forest, 52°43'18"N, 19°01'17"E, 29 April 2018, *M. Wolanin* (003461 UR); **DC73** – Włocławek, lawn, 52°40'29"N, 19°05'12"E, 29 April 2018, *M. Wolanin* (003480 UR); **FC13** – Piątnica (Fort Łomża), pastured grassland, 53°11'50"N, 22°06'52"E, 25 April 2016, *M. Wolanin* (003342 UR); **GD10** – Serpelice, lawn, 52°16'49"N, 23°03'01"E, 25 April 2016, *M. Wolanin* (003294 UR).

##### Notes.

**Figure 30.**
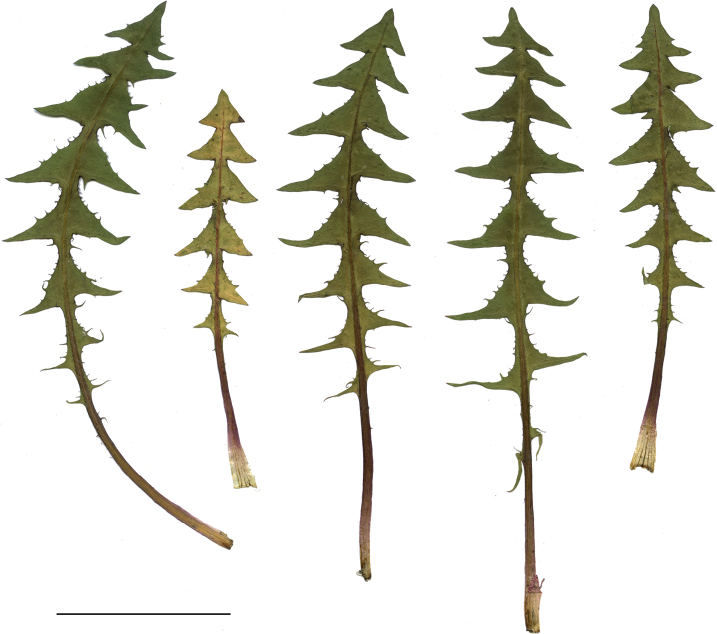
Variation in leaf shape in *T.proximum*; locality – Zaniemyśl (*M. Wolanin* 2016 UR). Scale bar: 5 cm.

**Figure 31.**
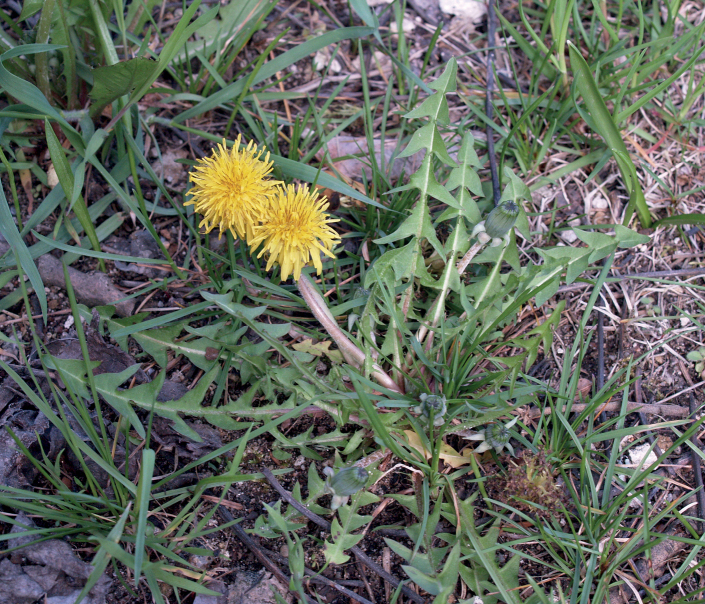
*Taraxacumproximum*; locality – Drezdenko, 2016, photo by M. Wolanin.

*T.proximum* is distinguished by elongated leaves with quite numerous side lobes (4–8 pairs) that are uniform, triangular-deltate, entire to denticulate. The capitulum is small (2–3 cm), convex, with numerous greenish-yellowish tubular ligules. Pollen absent. Achenes slender, reddish-brown (Figs 30, 31).

#### ﻿11. Taraxacum sandomiriense

Taxon classificationPlantaeAsteralesAsteraceae

Wolanin, Phytotaxa 375(2): 159. 2018.

8E2C3003-DD39-5D91-BA4A-270C21255F76

##### Type.

Poland, Kamień Łukawski, path on the edge of the loess slope (near the reserve), 50°41'N, 21°47'E, 6 May 2013, *M. Wolanin* (holotype KRA 476861; isotype KRA 476862).

##### Description.

Plants small to middle-sized, 6–10(–12) cm tall. ***Leaves*** greyish-green, dull, hairy on the upper side, approximately (3–)5–10(–15) cm long and 1–2.5 cm wide, usually 3–5 times longer than wide, blades narrowly oblanceolate, usually broadest in upper 1/3, with 6(–7) pairs of lateral lobes; lateral lobes opposite to remote; lateral lobes of the inner leaves narrowly triangular, patent, with an entire or slightly dentate, somewhat convex distal margin, proximal margin usually entire or with a few small teeth; lateral lobes of the outer leaves triangular, entire or with a few teeth at the distal margin; interlobes often toothed; terminal lobe of the inner leaves tripartite, mostly lingulate and entire on the margins; terminal lobe of the outer leaves triangular; petioles unwinged, pale purple, hairy. ***Scapes*** as long as or longer than leaves, hairy, especially just under the capitulum. ***Capitulum*** convex, 2.5–3.0 cm in diameter, yellow, outer strips grey-violet; inner bracts greyish-green, corniculate; outer bracts usually 10–14, lanceolate, usually 5–7 mm long, 2–2.5 mm broad, purplish green, with a distinct white hyaline margin (0.1–0.2 mm broad), recurved and strongly corniculate; stigmas greyish-yellow, pollen present. ***Achenes*** brown, with thin spinules in the upper part, 3.4–4.0 mm long (incl. the 0.8–1.2 mm long, narrowly conical pyramid) and (0.7–)0.8(–0.9) mm broad, rostrum 8.5–10 mm long, pappus white.

##### Flowering period.

April (May).

##### Habitat.

Rocky and loess slopes of river valleys with south-western exposure, in plant phytocenoses dominated by species characteristic to the *Festuco*-*Brometea* classes, usually in eroded or trampled areas. In Kamienna river valley (Sandomierz Upland, Gałkowice-Ocin place) this species was noted on the path and accompanied by *Achilleamillefolium*, *Alyssumalyssoides*, *Bromusinermis*, *Campanulasibirica*, *Centaureascabiosa*, *Clinopodiumvulgare*, *Cornussanguinea*, *Dactylisglomerata*, *Dianthuscarthusianorum*, *Euphorbiacyparissias*, *Falcariavulgaris*, *Festucarupicola*, *F.trachyphylla*, *Fragariavesca*, *Galiumverum*, *Hypericummaculatum*, *Inulahirta*, *Juniperuscommunis*, *Koeleriamacrantha*, *Leucanthemumvulgare*, *Linariavulgaris*, *Medicagofalcata*, *Peucedanumoreoselinum*, *Phleumphleoides*, *Plantagomedia*, *Poapratensis*, *Polygalacomosa*, *Potentillaarenaria*, *Prunusspinosa*, *Pyruspyraster*, *Rosacanina*, *R.dumalis*, *R.rubiginosa*, *Salviapratensis*, *Scabiosaochroleuca*, *Stachysrecta*, *Thymusmarschallianus*, *Ulmusminor*.

##### Somatic chromosome number.

24 (Wolanin et al. 2018).

##### General distribution.

Poland.

##### Distribution in Poland.

Species very rare, found only around Sandomierz (Wolanin et al. 2018; Fig. 23E).

##### Specimens examined.

**FE60** – Podgrodzie near Ćmielów, xerothermic grassland on rock outcrop, 50°54'24"N, 21°32'44"E, 17 April 2012, *M. Wolanin*, *M. Nykiel* (003431 UR); Podgrodzie, xerothermic grassland on rock outcrop, 50°54'24"N, 21°32'44"E, 13 April 2017, *M. Wolanin*, *M. N. Wolanin* (003421, 003422 UR); **FE82** – Gałkowice-Ocin, xerothermic grassland on SW slope, 50°44'43"N, 21°43'52"E, 17 April 2012, *M. Wolanin*, *M. Nykiel* (003442 UR); **FE92** – Kamień Łukawski, path on edge of loess slope near reserve, 50°41'04"N, 21°47'09"E, 6 May 2013, *M. Wolanin* (476861, 476862 KRA); Sandomierz (Strzelecka Hill), loess slope, 10 May 1924, *A. Kozłowska* (76947 KRA).

##### Notes.

**Figure 32.**
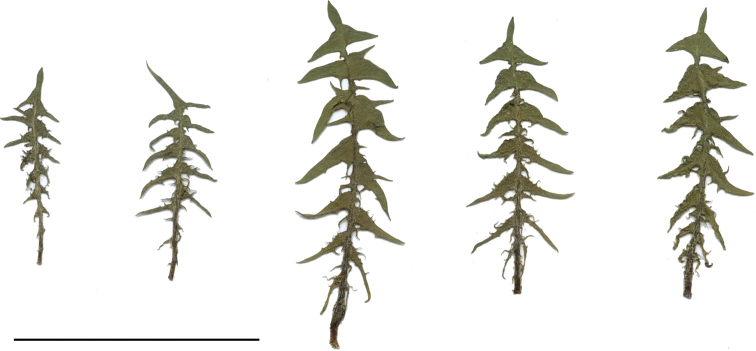
Variation in leaf shape in *T.sandomiriense*; locality – Podgrodzie (*M. Wolanin* 2012 UR). Scale bar: 5 cm.

**Figure 33.**
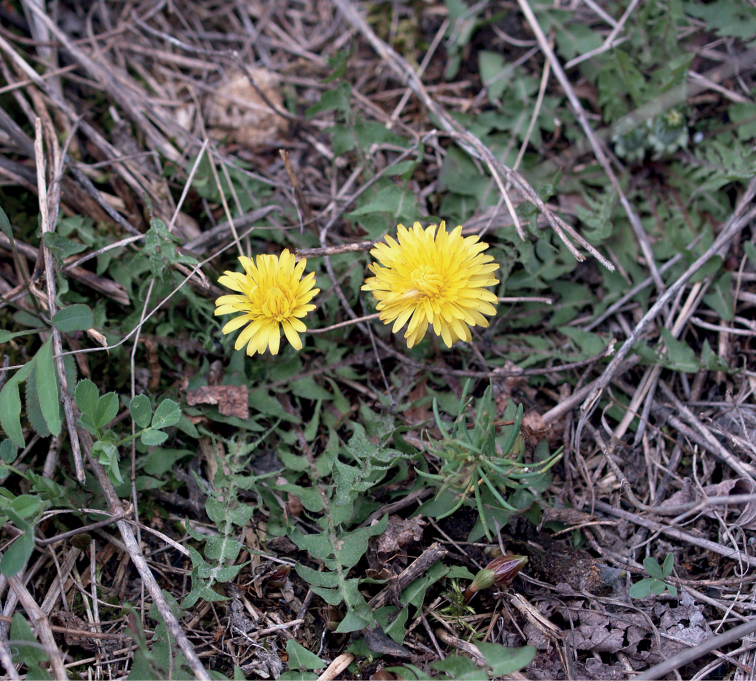
*Taraxacumsandomiriense*; locality – Podgrodzie, 2012, photo by M. Wolanin.

A distinct species with noticeably hairy leaves, purplish-green outer bracts with distinct white hyaline margin, recurved and strongly corniculate. Achenes are brown, with slender spinules in the upper part (Figs 32, 33).

#### ﻿12. Taraxacum scanicum

Taxon classificationPlantaeAsteralesAsteraceae

Dahlst., Ark. Bot. 10(11): 21. 1911.

BE5E5BD2-288D-5E67-9C18-A1A8F3876064

##### Type.

Sweden, Skåne, Lund, the garden of the infectious-diseases hospital, 21 May 1910, *E. L. Ekman* (lectotype in S [upper specimen], designated by Lundevall and Øllgaard 1999: 143).

##### Description.

Plants small to middle-sized, 10–20(–25) cm tall. ***Leaves*** dark green, almost glabrous, approximately (5–)7–12(–15) cm long and (1–)1.5–3(–4) cm wide, usually 6–7 times longer than wide, blades narrowly oblanceolate, usually broadest in upper 1/3, with (3–)4–6 pairs of lateral lobes, midribs often purple-brownish; lateral lobes opposite to remote; lateral lobes of the inner leaves usually dissected and toothed at the distal margin, slightly recurved, proximal margin usually entire; lateral lobes of the outer leaves triangular, usually toothed or/and incised at the distal margin; interlobes not or slightly crisped, often toothed only at the upper part of the leaf blade; terminal lobe of the inner leaves elongate with a protracted apex, quite often incised at the base; terminal lobe of the outer leaves with obtuse apical lobule; petioles unwinged, purple. ***Scapes*** as long as or longer than leaves, hairy, especially under the capitulum, often suffused purple-brownish. ***Capitulum*** convex, 3.0–3.5 cm in diameter, yellow, outer strips red-grey; inner bracts dark, glaucous greyish-green, somewhat pruinose, with or without a small lump; outer bracts usually 10–15, widely lanceolate, usually 7–9 mm long, 1.5–3.0 mm broad, grey-green, quite often suffused red-violet, with a narrow white hyaline margin (0.1–0.2 mm broad), recurved or patent, usually with small cornicules; stigmas dark greyish-green, pollen present. ***Achenes*** brown-red, with slender spinules in the upper part, 3.5–4.1 mm long (incl. the 0.8–1.2 mm long cone), rostrum (6.5–)7–8 mm long, pappus white.

##### Flowering period.

April–May.

##### Habitat.

Pine forest edges, forest clearings, paths, roadsides, most often in sunny, dry and sandy places. Plant communities participated by *T.scanicum* were dominated most often by species typical of sandy grasslands (*Sedo-Scleranthetea* class). In Wielkopolska Lowland (Ługi place) we recorded this species on a dry sandy roadside together with *Achilleamillefolium*, *Anthemisarvensis*, *Artemisiacampestris*, *A.vulgaris*, *Berteroaincana*, *Erophilaverna*, *Helichrysumarenarium*, *Plantagolanceolata*, *P.major*, *Poaannua*, *Potentillaargentea*, *Sedumacre*, Taraxacumsect.Taraxacum, *Veronicaarvensis*.

##### Somatic chromosome number.

24 (Grzesiuk et al. 2008; Wolanin and Musiał 2017), 25 (Małecka 1967, 1969).

##### General distribution.

Widespread European species reported from France, Great Britain, Italy, Croatia, Switzerland, Belgium, the Netherlands, Germany, Austria, Denmark, Hungary, the Czech Republic, Poland, Norway, Sweden, Finland, Moldova, Crimea, Ukraine, Lithuania, Latvia, Estonia and European Russia (Van Soest 1957, 1967; Doll 1973b; Tacik 1980; Fedorov 1989; Lundevall and Øllgaard 1999; Mosyakin and Fedoronchuk 1999; Uhlemann 2003; Głowacki et al. 2004; Trávníček et al. 2010; Wendt and Øllgaard 2015; Nobis et al. 2020b).

##### Distribution in Poland.

Scattered localities mainly in northern Poland, quite frequent (Fig. 23F).

##### Specimens examined.

**AB09** – Dźwirzyno, lawn on sandy soil, 54°09'45"N, 15°25'59"E, 2 May 2017, *M. Wolanin* (003273 UR); Grybowo, roadside ditch edge, 54°09'51"N, 15°28'21"E, 2 May 2017, *M. Wolanin* (003299 UR); **AB14** – Międzywodzie, roadside in forest, 54°00'35"N, 14°41'41"E, 30 April 2017, *M. Wolanin* (003331 UR); **AB16** – Pustkowo, pine forest edge, 54°04'08"N, 14°58'05"E, 2 May 2017, *M. Wolanin* (003312 UR); **AB22** – Przytór, roadside near pine forest edge, 53°53'25"N, 14°20'13"E, 30 April 2017, *M. Wolanin* (003351 UR); Warszów, roadside ditch near forest edge, 53°53'40"N, 14°18'54"E, 30 April 2017, *M. Wolanin* (003356 UR); **AB23** – Międzyzdroje, lawn on sandy soil, 53°56'15"N, 14°27'30"E, 30 April 2017, *M. Wolanin* (003353 UR); Międzyzdroje, lawn near beach entrance, 53°56'00"N, 14°26'59"E, 30 April 2017, *M. Wolanin* (003352 UR); Międzyzdroje, lawn, 53°56'10"N, 14°27'24"E, 30 April 2017, *M. Wolanin* (003332 UR); **AB32** – Ognica, sandy roadside, 53°52'47"N, 14°17'05"E, 30 April 2017, *M. Wolanin* (003319 UR); **AB47** – vicinity of Unibórz, roadside at pine forest edge, 53°48'48"N, 15°04'57"E, 30 April 2017, *M. Wolanin* (003371 UR); **AB53** – Trzebież, distr. Police, psammophilous grassland, 14 May 1999, *Z. Głowacki* (527640 KRAM); **BA59** – Ustka, sandy square in pine forest, 54°35'15"N, 16°52'48"E, 1 May 2017, *M. Wolanin* (003286 UR); Ustka, sandy roadside, 54°35'17"N, 16°53'07"E, 1 May 2017, *M. Wolanin* (003260 UR); **BC18** – Piła, sandy roadside, 53°09'01"N, 16°47'24"E, 29 April 2017, *M. Wolanin* (003357, 003370 UR); **BC51** – Drezdenko, ruderal square in cemetery, 52°50'07"N, 15°49'25"E, 18 April 2016, *M. Wolanin* (003494 UR); **BC52** – Chełst, cemetery lawn, 52°49'17"N, 15°57'35"E, 18 April 2016, *M. Wolanin* (003544 UR); **BC61** – Sowia Góra, roadside No 160, 52°41'55"N, 15°50'41"E, 18 April 2016, *M. Wolanin* (003493 UR); **BD42** – Świętno, roadside, 52°00'29"N, 16°03'29"E, 19 April 2016, *M. Wolanin* (003518 UR); **BD43** – Kębłowo, ruderal area close to cemetery fence, 52°03'06"N, 16°06'35"E, 19 April 2016, *M. Wolanin* (003381 UR); **BD53** – Kaszczor, cemetery lawn, 51°57'20"N, 16°10'01"E, 19 April 2016, *M. Wolanin* (003384 UR); **BD77** – Bojanowo, Półwiejska street 12, gap between pavement and kerb, 51°42'36"N, 16°44'53"E, 19 April 2016, *M. Wolanin* (003385 UR); **CA38** – W of Chłapowska Valley outlet, cliff slope, 25 June 1970, *W. Chojnacki* (153/01 UGDA); **CA43** – Łeba, along path in pine forest, 54°46'13"N, 17°35'28"E, 3 May 2019, *M. Wolanin* (003573 UR); Łeba, clearing in pine forest, 54°45'49"N, 17°32'31"E, 2 May 2019, *M. Wolanin* (003576 UR); **CD30** – Majdany, sandy roadside in pine forest, 52°08'02"N, 17°10'58"E, 17 April 2016, *M. Wolanin* (003508 UR); Zaniemyśl, cemetery lawn, 52°09'01"N, 17°10'09"E, 17 April 2016, *M. Wolanin* (003509 UR); **CD31** – Murzynowo Leśne, sandy square close to shop, 52°09'17"N, 17°20'25"E, 17 April 2016, *M. Wolanin* (003507 UR); **CD40** – Błażejewo, sandy grassland in cemetery, 52°00'01"N, 17°08'37"E, 18 April 2016, *M. Wolanin* (003383 UR); Jarosławki, sandy roadside at pine forest edge, 52°03'07"N, 17°10'22"E, 16 April 2016, *M. Wolanin* (003416 UR); Kiełczynek, lawn, 52°04'11"N, 17°12'49"E, 16 April 2016, *M. Wolanin* (003418 UR); Książ Wielkopolski, roadside in forest, 52°04'02"N, 17°14'44"E, 16 April 2016, *M. Wolanin* (003537 UR); Ługi, sandy roadside, 51°59'17"N, 17°11'13"E, 16 April 2016, *M. Wolanin* (003536 UR); **DA40** – Jastrania, gap between pavement and kerb, 54°42'49"N, 18°38'17"E, 9 May 2016, *M. Wolanin* (003555 UR); **DA51** – Hel (Leśna street), sandy square at pine forest edge, 54°36'08"N, 18°48'55"E, 8 May 2016, *M. Wolanin* (003501 UR); Hel, meadow close to church, 8 May 1997, *K. Błaszkiewicz* (058153 KTU); **DA76** – Piaski, sandy roadside, 54°25'11"N, 19°34'00"E, 10 May 2016, *M. Wolanin* (003402 UR); **DA81** – Gdańsk (Stogi), path in light pine forest, 54°22'24"N, 18°43'38"E, 7 May 2016, *M. Wolanin* (003399, 003556 UR); **DC41** – Sąsieczno, pine forest edge, 52°57'03"N, 18°50'38"E, 29 April 2018, *M. Wolanin* (003472 UR); **DC44** – vicinity of Adamów, dry square on pine forest edge, 52°56'52"N, 19°17'45"E, 29 April 2018, *M. Wolanin* (003478 UR); **DC52** – Dąbrówka, roadside in forest, 52°53'42"N, 18°57'51"E, 29 April 2018, *M. Wolanin* (003470 UR); between Wakole and Dąbrówka, roadside in pine forest, 52°51'40"N, 18°58'09"E, 29 April 2018, *M. Wolanin* (003449 UR); **DC57** – Borkowo Kościelne near Sierpc, cemetery lawn, 52°50'53"N, 19°42'39"E, 28 April 2018, *M. Wolanin* (003455 UR); **DC63** – Rachcinek, dry roadside, 52°44'23"N, 19°01'15"E, 29 April 2018, *M. Wolanin* (003458 UR); **DE51** – St. Genowefa Hill, distr. Wieluń, 12 June 1965, *H. Błaszczyk* (94960 KRA); **DF26** – Ogrodzieniec near Zawiercie, calcareous rocks, 21 May 1965, *A. Jasiewicz* (439038 KRAM); **DF68** – between Kryspinów and Bielany, sunny hillock, 16 May 1976, *H.*, *T.* & *J. Tacik* (392442 KRAM); **ED11** – Młodzieszyn, pine forest edge, 52°19'00"N, 20°12'23"E, 28 April 2018, *M. Wolanin* (003474, 003475 UR); **EE83** – Zalejowa Mt., fissure in rock, 50°49'07"N, 20°27'30"E, 20 April 2016, *M. Wolanin* (003558 UR); **FC13** – Piątnica (Fort Łomża), pastured grassland, 53°11'50"N, 22°06'53"E, 25 April 2016, *M. Wolanin* (003374, 003567 UR); **FC24** – Zbrzeźnica, sandy bank close to road No 63, 53°02'23"N, 22°09'57"E, 22 April 2016, *M. Wolanin* (003531 UR); Wygoda, sandy bank close to road No 63, 53°04'59"N, 22°08'42"E, 22 April 2016, *M. Wolanin* (003559 UR); **FC50** – Kobylin distr. Goworowo, grassland close to road, 19 May 1992, *Z. Głowacki* (0386425 KRA); **FE85** – Łysaków – Kolonia, sandy roadside close to pine forest edge, 50°45'23"N, 22°07'29"E, 19 April 2019, *M. Wolanin* (003586 UR); **FE96** – Domostawa, scrub on sandy soil, near S19 road parking lot, 50°37'14"N, 22°17'14"E, 22 April 2018, *M. Wolanin* (003463, 003465, 003466 UR); **GF00** – Tereszpol (Zygmunty), sandy path in pine forest, 50°33'56"N, 22°53'56"E, 2 May 2018, *M. Wolanin* (003467 UR).

##### Notes.

**Figure 34.**
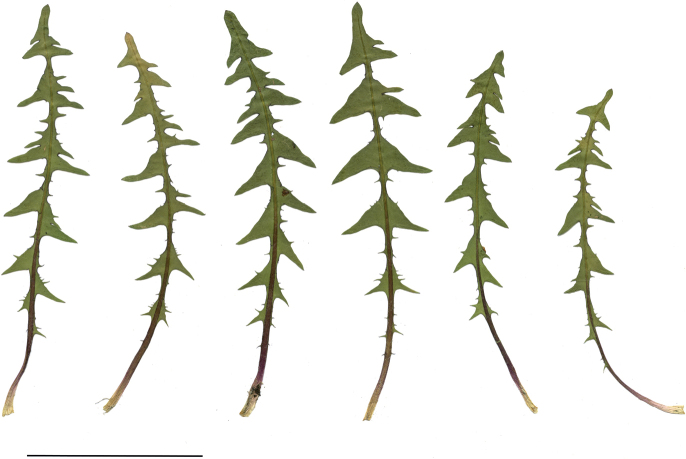
Variation in leaf shape in *T.scanicum*; locality – Gdańsk Stogi (*M. Wolanin* 2016 UR). Scale bar: 5 cm.

**Figure 35.**
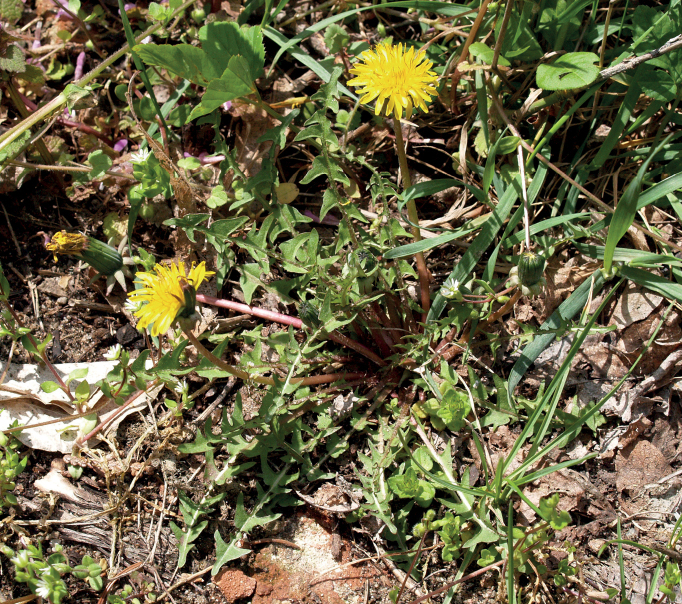
*Taraxacumscanicum*; locality – Drezdenko, 2016, photo by M. Wolanin.

Plants with narrow leaves. Lobes tend to be dissected and petioles suffused purple. Outer bracts grey-green with white hyaline margin, often lightly suffused red-violet, recurved or patent. Fully flowering capitulum convex, yellow, sigmas dark. Fruits brown-red, deeply coloured (Figs 34, 35).

#### ﻿13. Taraxacum tenuilobum

Taxon classificationPlantaeAsteralesAsteraceae

(Dahlst.) Dahlst., Acta Fl. Sueciae 1: 47, 50, 85. 1921.

3845B10A-078B-5C59-BE7C-F046DEC75E5B

Taraxacum erythrospermum subsp. tenuilobum Dahlst., Bot. Not., 1905: 167. 1905.Basionym.

##### Type.

Sweden, Dalsland, Mo, Ojersbyn, on rock, 24 May 1901, *P.A. Larsson* (lectotype in S, designated by G. Haglund in Doll 1973: 86; isolectotype in S).

##### Description.

Plants small to middle-sized, (5–)10–15 cm tall. ***Leaves*** grey-green, almost glabrous or with few barely visible hairs, approximately (5–)7–12(–15) cm long and (1.5–)2.5–3.5(–4) cm wide, usually 4–5 times longer than wide, blades narrowly oblanceolate, usually broadest in upper 1/3, with 3–7 pairs of lateral lobes; lateral lobes opposite to remote; lateral lobes of the inner leaves strongly dissected, somewhat recurved and twisted, quite often slightly widening at the apex, often with parallel small and acute lobes at the distal margin base; lateral lobes of the outer leaves narrowly triangular, slightly recurved or patent, quite often incised or toothed at the distal margin base; interlobes long, often lobulate; terminal lobe of the inner leaves elongate with a protracted apex; terminal lobe of the outer leaves tripartite-subsagitate, quite often with the apical lobule slightly widening at the apex; petioles unwinged, purple. ***Scapes*** as long as or shorter than leaves, hairy, especially just under the capitulum. ***Capitulum*** convex, 2.5–3.5 cm in diameter, yellow, outer strips grey-violet; inner bracts greyish-green, often with small lumps; outer bracts usually 10–15, narrowly lanceolate, usually 6–8 mm long, 1.5–2 mm broad, bright greyish-green, suffused with violet, faintly bordered (up to 0.05 broad), arcuate, without corniculation or sometimes with small cornicules; stigmas dark greyish-green, pollen present. ***Achenes*** red-brown, in the upper part with slender spinules, 3.5–4.0 mm long (incl. the 0.8–1.1 mm long, cylindrical cone), rostrum 6–7 mm long, pappus white.

##### Flowering period.

April–May.

##### Habitat.

In the northern part of Poland, this species grows most often in dry and sandy habitats, such as sandy grasslands, roadsides of forest roads, edges of pine fo­rests, paths, cliffs, dunes, and lawns. In southern Poland, we noticed this species most often in rock grasslands (in eroding and trampled areas). Plant communities with the participation of *T.tenuilobum* were predominated by species typical to the *Festuco-Brometea* (especially in S Poland) and *Sedo-Scleranthetea* classes. In Świętokrzyskie Mts (Miedzianka place) we noted this species in rocky grassland growing together with *Alliummontanum*, *Arenariaserpyllifolia*, *Artemisiacampestris*, Camelinamicrocarpasubsp.sylvestris, *Campanulasibirica*, *Centaureastoebe*, *Cerastiumsemidecandrum*, *Dianthuscarthusianorum*, *Euphorbiacyparissias*, *Galiummollugo*, *Holosteumumbellatum*, *Medicagofalcata*, *Plantagomedia*, *Poacompressa*, *P.pratensis*, *Potentillaargentea*, *Ranunculusbulbosus*, *Salviapratensis*, *Sanguisorbaminor*, *Sedumacre*, *Silenenutans*, *Stachysrecta*, *Thymuspulegioides*, *Verbascumnigrum*, *Vincetoxicumhirundinaria*, *Violaarvensis*. On the coast of the Baltic Sea (Łeba place) we observed these species on the sandy edge of the pine forest, accompanied by *Carexarenaria*, *Cerastiumsemidecandrum*, *Erophilaverna*, *Potentillaargentea*, *Vicialathyroides*.

##### Somatic chromosome number.

24 (Wolanin and Musiał 2017), 25 (Małecka 1969).

##### General distribution.

Mainly NE part of Europe. Species reported from Switzerland, Germany, the Netherlands, Denmark, Poland, Sweden, Norway, Crimea, Moldova, Ukraine, Latvia, Estonia and European Russia (Van Soest 1969; Tutin et al. 1976; Tacik 1980; Fedorov 1989; Lundevall and Øllgaard 1999; Mosyakin and Fedoronchuk 1999; Uhlemann 2003; Wendt and Øllgaard 2015).

##### Distribution in Poland.

**Figure 36.**
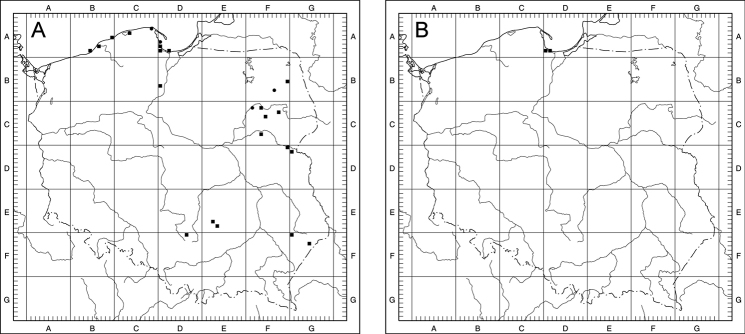
Distribution maps of Taraxacumsect.Erythrosperma in Poland **A***T.tenuilobum***B***T.tortilobum*; black square – localities recorded during field studies, black circle – other localities known from herbarium data.

Localities grouped in 4 separate areas in northern and southern Poland; quite frequent only in Podlachia and on the coast of the Baltic Sea (Fig. 36A).

##### Specimens examined.

**BA59** – Ustka, path, 54°35'25"N, 16°53'07"E, 1 May 2017, *M. Wolanin* (003309 UR); **BA76** – Darłówko, sands, 54°26'45"N, 16°23'41"E, 1 May 2017, *M. Wolanin* (003323 UR); **BA84** – Łazy, path in pine forest, 54°18'16"N, 16°11'16"E, 1 May 2017, *M. Wolanin* (003346 UR); **CA38** – 1,5 km E of Rozewie, cliff, 30 May 1961, *Monk*/*Mark* (152/2 UGDA); **CA43** – Łeba, path in pine forest, 54°46'05"N, 17°34'06"E, 1 May 2017, *M. Wolanin* (003336 UR); **DA60** – south of Babie Doły, cliff, 12 May 1970, *W. Chojnacki* (153/01 UGDA); **DA70** – Sopot, lawn near promenade, 54°27'26"N, 18°33'43"E, 8 May 2016, *M. Wolanin* (003296 UR); **DA80** – Wisłoujście (Gdańsk), lawn, 54°23'37"N, 18°40'36"E, 7 May 2016, *M. Wolanin* (003552 UR); **DA82** – Świbno, sandy roadside in forest, 54°20'18"N, 18°56'12"E, 10 May 2016, *M. Wolanin* (003283 UR), **DB60** – near Jaszczerek, sandy roadside, 53°37'02"N, 18°35'16"E, 1 May 2019, *M. Wolanin* (003575 UR), **DF06** – Kroczyce, grassland and paths on calceolus rock (SW slope), 50°34'18"N, 19°31'47"E, 1 May 2013, *M. Wolanin* (003359 UR); Podlesice, rock close to cave, grassland on rock, 50°34'30"N, 19°31'32"E, 1 May 2021, *M. Wolanin* (003593 UR); Mirów, grassland on rock overgrown by shrubs, 50°36'53"N, 19°28'51"E, 1 May 2021, *M. Wolanin* (003595 UR); **EE72** – Miedzianka hill, grassland on rock, 50°50'49"N, 20°21'32"E, 11 April 2016, *M. Wolanin* (003514 UR); **EE83** – Sosnówka hill, grassland on rock, 50°48'24"N, 20°26'15"E, 11 April 2016, *M. Wolanin* (003500 UR); **FB59** – between Wrotki and Mogielnica, dry pasture, 53°39'45"N, 22°58'11"E, 24 April 2016, *M. Wolanin* (003270 UR); **FB76** – Sośnia, dunes on pasture, 8 May 2003, *Z. Głowacki* (0388261 KRA); **FC11** – Czartoria, hillock close to river, 8 May 2016, *T. Grużewska* (MPD); **FC13** – Piątnica (Fort Łomża), pastured sandy grassland, 53°11'48"N, 22°07'00"E, 24 April 2016, *M. Wolanin* (003539 UR); **FC27** – Truskolasy-Lachy, stoned field road close to old excavation, 53°02'23"N, 22°42'11"E, 2 May 2018, *M. Wolanin* (003481); **FC34** – Zbrzeźnica, psammophilous grassland, 53°01'24"N, 22°10'11"E, 26 April 2016, *M. Wolanin* (003546 UR); **FC73** – Borowe nad Bugiem, Bug river sandy terrace, 52°40'22"N, 22°00'41"E, 26 April 2016, *M. Wolanin* (003545 UR); **FD09** – Bużka, sandy roadside, 52°21'26"N, 22°53'55"E, 25 April 2016, *M. Wolanin* (003484 UR); **GD10** – Serpelice, lawn, 52°16'49"N, 23°03'01"E, 25 April 2016, *M. Wolanin* (003257 UR); **GF00** – Tereszpol near Biłgoraj, lawn, 50°33'13"N, 22°55'54"E, 25 April 2016, *M. Wolanin* (003468 UR); **GF24** – Bełżec, grassland on closed railway track, 50°22'45"N, 23°26'51"E, 18 April 2019, *M. Wolanin* (003587 UR).

##### Notes.

**Figure 37.**
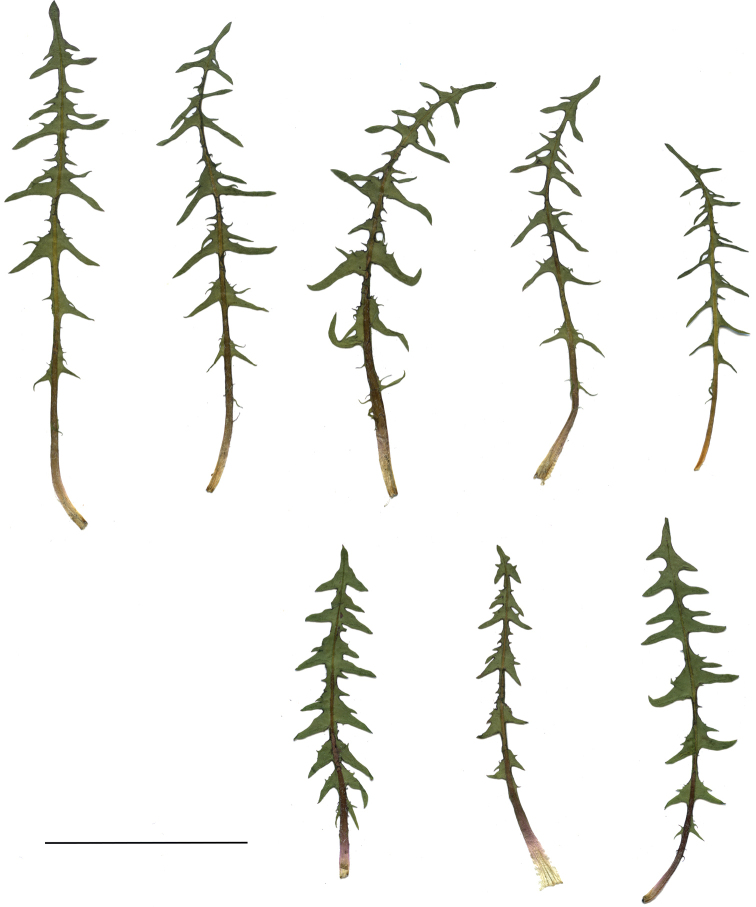
Variation in leaf shape in *T.tenuilobum*; locality – Kroczyce (*M. Wolanin* 2016 UR). Scale bar: 5 cm.

**Figure 38.**
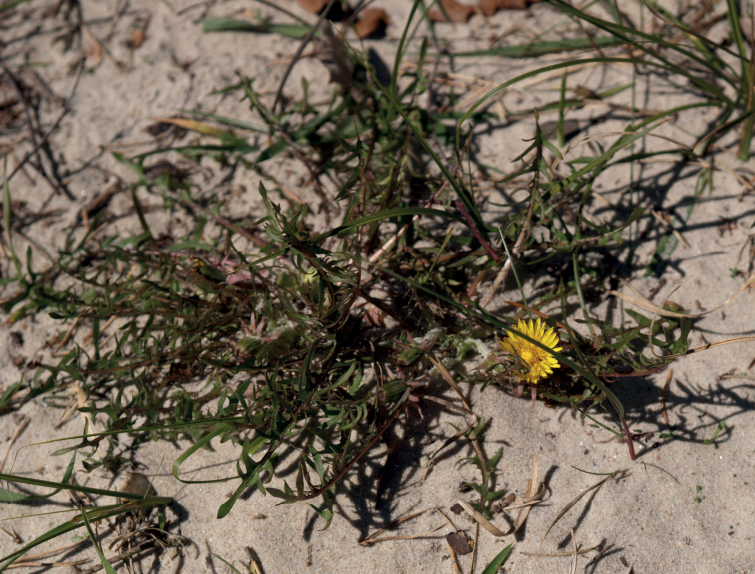
*Taraxacumtenuilobum*; locality – Łeba, 2017, photo by M. Wolanin.

Plant with distinct, strongly incised and narrow side lobes, and tongue-shaped terminal lobe apex. Outer phyllaries are narrowly lanceolate, arcuate, faintly bordered. Juvenile plants can be confused with *T.scanicum*, which has significantly less dissected side lobes, and the outer bracts of which are wider (with distinct hyaline margin, 0.1–0.2 mm broad), recurved or patent (Figs 37, 38).

#### ﻿14. Taraxacum tortilobum

Taxon classificationPlantaeAsteralesAsteraceae

Florstr., Acta Soc. Fauna Fl. Fenn. 39(4): 11. 1915.

7E68A406-19A0-57AA-8A16-CA93A3145ECB

##### Type.

Finland, Satakunta, Pori (Bjomeborg), Rafso (Reposaari), the cemetery area, 8 June 1911, *B. Florstrom* (lectotype H 068135, designated by Doll 1973: 49; isolectotype in H 202526, 202527, 202538, 202541-202543 and in S).

##### Description.

Plants small to middle-sized, up to 15(–20) cm tall. ***Leaves*** greyish-green to light green, sparsely hairy, approximately (5–)7–10(–14) cm long and (1.5–)2–3(–4.5) cm wide, usually 3–5 times longer than wide, blades elliptical to oblanceolate, with 4–6 pairs of lateral lobes; lateral lobes opposite to remote; lateral lobes of the inner leaves patent or recurved, widened at the base, crisped, with nume­rous filiform teeth at the distal and proximal margins, usually curled at the apex; lateral lobes of the outer leaves triangular, entire or with a few filiform teeth at margins; interlobes frequently dentate; terminal lobe of the inner leaves tripartite with an elongate tongue-shaped tip; terminal lobe of the outer leaves triangular or tripartite, often with a tongue-shaped tip; petioles narrowly winged, pale purplish-red. ***Scapes*** as long as or longer than leaves, somewhat hairy, green or suffused pale purplish-red. ***Capitulum*** convex, 3–4 cm in diameter, pale yellow, outer strips grey-purple; inner bracts greyish-green, corniculate; outer bracts usually 12–14, ovate to lanceolate, usually 6–7(– 8) mm long and (–1.5)2–3 mm broad, grey-green, suffused with purple, with a white hyaline margin 0.1–0.2 mm broad, loosely adpressed to obliquely spreading, purple at apex, corniculate; stigmas grey-purple, pollen present. ***Achenes*** pale grey-brown, achene body spinulose above, 4.7–5.3 mm long (incl. the 1.4–1.9 mm long, narrowly conical cone); for specimens growing in full light achene measurements are 4–4.4 mm (incl. the 0.9–1.3 mm long cone).

##### Flowering period.

(April) May.

##### Habitat.

Sandy pine forest edges, dry lawns, scrubs. On the coast of the Baltic Sea (Gdańsk Stogi) we noticed this species on a sandy path at the edge of a pine–false acacia forest (growing together with *Elymusrepens*) and on the scrub edge, accompanied by *Ballotanigra*, *Dactylisglomerata*, *Erigeronannuus*, *Ficariaverna*, *Geraniumpusillum*, *G.robertianum*, *Lamiumpurpureum*, *Poanemoralis*, *P.pratensis*, *Stellariamedia*, *Taraxacumproximum*, *Veronicahederifolia* s.l.

##### Somatic chromosome number.

24 (Wolanin and Musiał 2018).

##### General distribution.

Widespread European species reported from Spain, Great Britain, France, Corsica, Italy, Switzerland, Germany, Belgium, the Netherlands, Germany, Denmark, Poland, Sweden, Finland, Ukraine, Latvia, Estonia (Van Soest 1967; Richards 1969; Adema et al. 1982; Lundevall and Øllgaard 1999; Mosyakin and Fedo­ronchuk 1999; Uhlemann 2003; Wendt and Øllgaard 2015; Wolanin and Musiał 2018).

##### Distribution in Poland.

Species scarce, noted only in Gdańsk (Baltic Sea seashore) (Fig. 36B).

##### Specimens examined.

**DA80** – Gdańsk, Roland pleasure ground, lawn on sandy ground, 54°24'45"N, 18°36'18"E, 08.05.2016, *M. Wolanin* (003289 UR); **DA81** – Gdańsk (Stogi), sandy place on pine forest edge (along concrete walkway), 54°22'27"N, 18°43'40"E, 7 May 2016, *M. Wolanin* (003263, 003276, 003302 UR); Gdańsk (Stogi), sands, 54°22'08"N, 18°43'31"E, 8 May 2016, *M. Wolanin* (003315 UR).

##### Notes.

**Figure 39.**
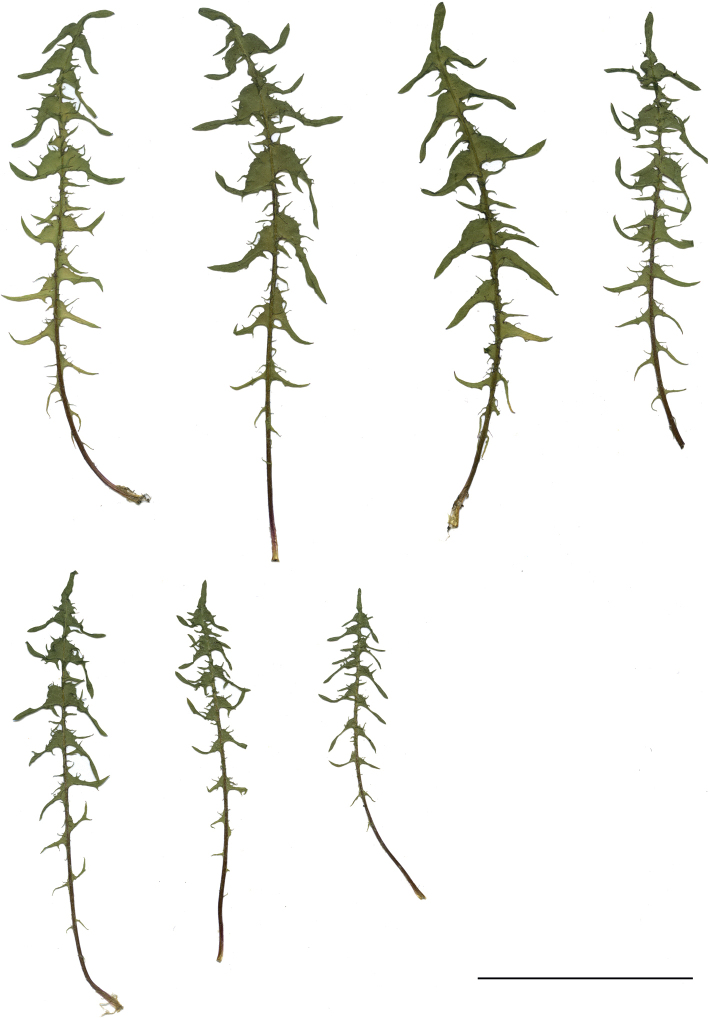
Variation in leaf shape in *T.tortilobum*; locality – Gdańsk Stogi (*M. Wolanin* 2016 UR). Scale bar: 5 cm.

**Figure 40.**
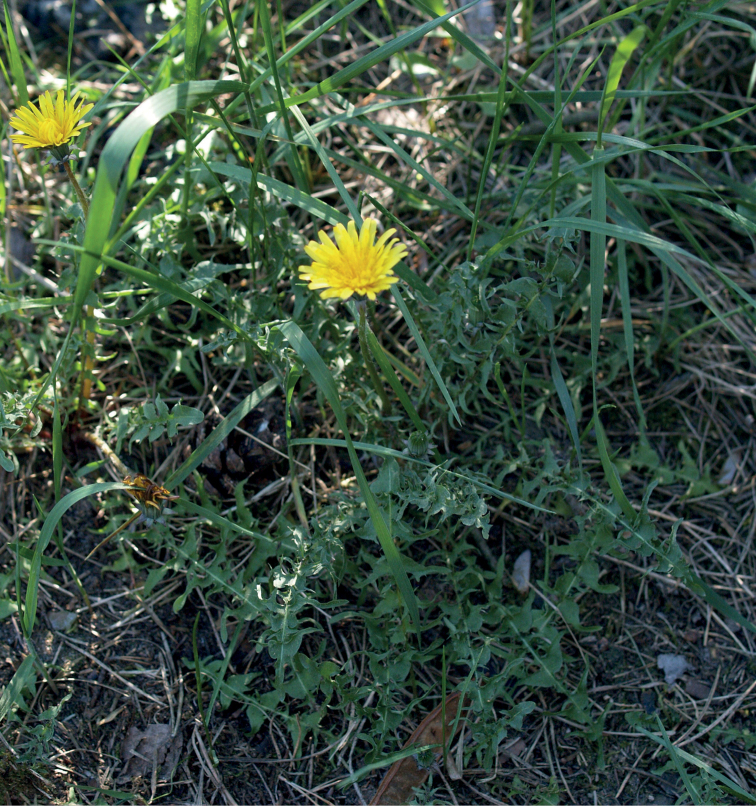
*Taraxacumtortilobum*; locality – Gdańsk Stogi, 2016, photo by M. Wolanin.

Species included in the *Dissimilia* group, easily identified by a combination of pale grey-brown achenes, leaves strongly crisped, lateral lobes often toothed and curled, outer phyllaries loosely adpressed to obliquely spreading (Figs 39, 40).

## ﻿Species not confirmed in Poland

Based on the literature data, 25 Taraxacum species from the section Erythrosperma have been reported from Poland to date (Table 1). However, the presence of 10 species listed by Tacik (1980), i.e.: *T.austriacum* Soest (=*T.erythrospermum* Andrz. & Besser), *T.brunneum* Soest, *T.laetiforme* Dahlst. (=*T.discretum* H. Øllg.), *T.falcatum* Brenner, *T.fulvum* Raunk., *T.gracillimum* Soest, *T.laetum* (Dahlst.) Dahlst., *T.leptocarpum* Saarsoo, *T.marginatum* Dahlst., *T.simile* Raunk, and included in successive editions of the checklist (Mirek et al. 1995, 2002, 2020), were not confirmed during the revision of the herbarium materials. All of the above-mentioned species had been misidentified with other species. Moreover, the occurrence of the species was also not confirmed during the field studies. It is worth menti­oning that, with the exception of *T.erythrospermum*, all of these species are also absent in the neighbouring countries/regions (eastern Germany, Czech Republic) (Uhlemann 2003; Trávníček et al. 2010). The specimens of *T.erythrospermum* collected in the vicinity of Kraków (Tacik 1980) were misidentified and actually belonged to *T.danubium* (Wolanin and Musiał 2018). In 2005, Vašut et al. (2005) reported the presence of *T.maricum* in Solec, however, their determination of the specimens was uncertain. During field studies, we could not confirm the presence of *T.maricum* in Solec, despite intensive field penetration at the locality. Thus, in the face of the lack of additional herbarium material of this species from Poland, we decided to exclude it from the list.

## ﻿Threats

**Figure 41.**
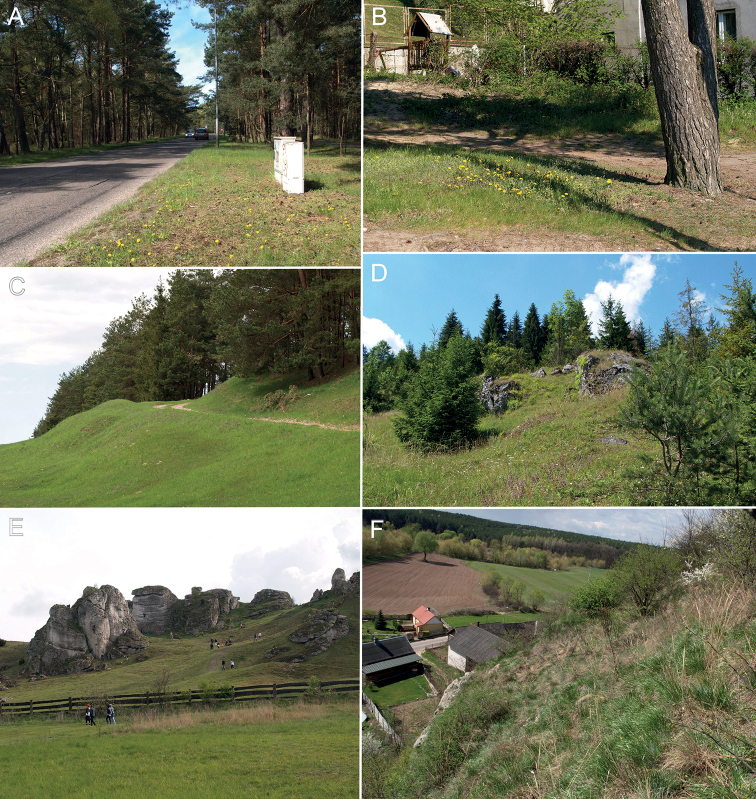
Habitats of dandelions of the sect. Erythrosperma in various parts of Poland **A***Taraxacumplumbeum* on sandy roadside in Dźwirzyno, 2016 **B***T.lacistophyllum* on dry lawn in Jastrzębia Góra, 2016 **C** dry rocky SW slope in Jeleniewo – habitat of *T.bellicum*, 2018 **D** xerothermic grassland in Jaworki (Pieniny Mts) – habitat of *T.parnassicum*, 2015 **E** limestone rocks in Olsztyn – habitat of *T.danubium*, *T.parnassicum* and *T.brachyglossum*, 2020 **F** rocky grassland in Podgrodzie – habitat of *T.sandomiriense* and *T.bellicum*, 2018, photos by M. Wolanin.

Using the IUCN (2022) threat categories, most of the investigated species (*T.bellicum*, *T.brachyglossum*, *T.lacistophyllum*, *T.plumbeum*, *T.proximum*, *T.scanicum*, *T.tenuilobum*) should be considered as of least concern (LC) in Poland. The species listed above are rare in Poland, growing in large dispersion (*T.bellicum*, *T.brachyglossum*, *T.plumbeum*, *T.scanicum*, *T.tenuilobum*), the others (*T.lacistophyllum*, *T.proximum*) occur more regionally, often abundantly, in semi-ruderal habitats (Fig. 41A–C). One gets the impression that human activity causes them more benefits than harm, for example, in Wielkopolska Lowland, *T.scanicum*, *T.proximum*, *T.plumbeum*, *T.bellicum* grow on intensively trampled paths, on roadsides or even in cemeteries. We also included the rarer plants (*T.disseminatum*, *T.dissimile*) in this category, due to the relatively large area of occurrence and the lack of noticeable factors that could threaten them at present. *T.parnassicum* is considered a species near to threat (NT) due to its close relation to specific, rare habitats and observed unfavourable habitat transformations that clearly threaten this species, e.g. xerothermic rock grasslands overgrowing due to lack of grazing (Fig. 41D). A significant part of the private property limestone rocks in the Kraków-Częstochowa Upland is also successively fenced, which definitely accelerates the overgrowing by shrubs due to the lack of touristic exploration. In the Lower Silesia, many localities of *T.parnassicum* reported in the nineteenth and second half of the twentieth century are now most likely historical, which may have been caused by the intensification of agriculture in this area and secondary succession in closed sand mines. *T.tortilobum* is classified as a vulnerable species (VU) due to very small and limited populations (the species was found only in Gdańsk). *T.danubium* should be recognised as an endangered species (EN) due to its limited range in Poland, a very low number of sites and very significant fluctuations observed in the number of mature individuals. It seems that a significant factor preserving this species in the largest localities (Olsztyn, Skały Twardow­skiego in Kraków) is their being re­creational destinations (Fig. 41E). *T.cristatum* should be considered critically endangered (CR) in Poland due to the extremely low number of its localities, small populations and the observed decline in the number of individuals. The same risk category (CR) was assigned to *T.sandomiriense* due to the extremely small number of sites and mature plants within them as well as the tendency for its habitats to decline (very rare and specific to this species; Fig. 41F).

## Supplementary Material

XML Treatment for Taraxacum sect. Erythrosperma

XML Treatment for Taraxacum bellicum

XML Treatment for Taraxacum brachyglossum

XML Treatment for Taraxacum cristatum

XML Treatment for Taraxacum danubium

XML Treatment for Taraxacum disseminatum

XML Treatment for Taraxacum dissimile

XML Treatment for Taraxacum lacistophyllum

XML Treatment for Taraxacum parnassicum

XML Treatment for Taraxacum plumbeum

XML Treatment for Taraxacum proximum

XML Treatment for Taraxacum sandomiriense

XML Treatment for Taraxacum scanicum

XML Treatment for Taraxacum tenuilobum

XML Treatment for Taraxacum tortilobum

## Supplementary materials

Supplementary material 1 Genetic distance between *Taraxacum* genotypes Data type genetic https://binary.pensoft.net/file/833378This dataset is made available under the Open Database License (http://opendatacommons.org/licenses/odbl/1.0/). The Open Database License (ODbL) is a license agreement intended to allow users to freely share, modify, and use this Dataset while maintaining this same freedom for others, provided that the original source and author(s) are credited.Mateusz Wolanin, Ewelina Klichowska, Iwona Jedrzejczyk, Monika Rewers, Marcin Nobis
