# Supplementary material for: ﻿Taxonomy and distribution of Taraxacum sect. Erythrosperma (Asteraceae) in Poland
Source: PhytoKeys. 2023 Apr 5;224:1–88. doi: 10.3897/phytokeys.224.99463 (PMC10311454; doi:10.3897/phytokeys.224.99463)
Supplement: Supplementary material 1 — Genetic distance between Taraxacum genotypes [file phytokeys-224-001_article-99463__-s001.docx]

| Genotype | 1 | 2 | 3 | 4 | 5 | 6 | 7 | 8 | 9 | 10 | 11 | 12 | 13 | 14 | 15 | 16 | 17 | 18 | 19 | 20 | 21 | 22 | 23 | 24 | 25 | 26 | 27 | 28 | 29 | 30 | 31 | 32 | 33 |
| --- | --- | --- | --- | --- | --- | --- | --- | --- | --- | --- | --- | --- | --- | --- | --- | --- | --- | --- | --- | --- | --- | --- | --- | --- | --- | --- | --- | --- | --- | --- | --- | --- | --- |
| 1. 56 *T. bellicum* (Siematycze) | 0 |  |  |  |  |  |  |  |  |  |  |  |  |  |  |  |  |  |  |  |  |  |  |  |  |  |  |  |  |  |  |  |  |
| 1. 57 *T. bellicum* (Klimaszewnica 2) | 0.04 | 0 |  |  |  |  |  |  |  |  |  |  |  |  |  |  |  |  |  |  |  |  |  |  |  |  |  |  |  |  |  |  |  |
| 1. 58 *T. bellicum* (Klimaszewnica) | 0.09 | 0.08 | 0 |  |  |  |  |  |  |  |  |  |  |  |  |  |  |  |  |  |  |  |  |  |  |  |  |  |  |  |  |  |  |
| 1. 59 *T. bellicum* (Arbasy) | 0.10 | 0.10 | 0.14 | 0 |  |  |  |  |  |  |  |  |  |  |  |  |  |  |  |  |  |  |  |  |  |  |  |  |  |  |  |  |  |
| 1. 2 *T. brachyglossum* (Kusieta) | 0.22 | 0.23 | 0.25 | 0.23 | 0 |  |  |  |  |  |  |  |  |  |  |  |  |  |  |  |  |  |  |  |  |  |  |  |  |  |  |  |  |
| 1. 31 *T. brachyglossum* (Kusieta) | 0.23 | 0.25 | 0.25 | 0.23 | 0.11 | 0 |  |  |  |  |  |  |  |  |  |  |  |  |  |  |  |  |  |  |  |  |  |  |  |  |  |  |  |
| 1. 13 *T. cristatum* (Grzaby Bolminskie) | 0.26 | 0.27 | 0.31 | 0.23 | 0.32 | 0.33 | 0 |  |  |  |  |  |  |  |  |  |  |  |  |  |  |  |  |  |  |  |  |  |  |  |  |  |  |
| 1. 28 *T. danubium* (Olsztyn) | 0.31 | 0.31 | 0.33 | 0.31 | 0.31 | 0.32 | 0.34 | 0 |  |  |  |  |  |  |  |  |  |  |  |  |  |  |  |  |  |  |  |  |  |  |  |  |  |
| 1. 51 *T. danubium* (Gora Sfinks) | 0.16 | 0.17 | 0.20 | 0.16 | 0.23 | 0.24 | 0.27 | 0.22 | 0 |  |  |  |  |  |  |  |  |  |  |  |  |  |  |  |  |  |  |  |  |  |  |  |  |
| 1. 52 *T. danubium* (Kostrze, Krakow) | 0.27 | 0.26 | 0.30 | 0.24 | 0.31 | 0.31 | 0.27 | 0.22 | 0.16 | 0 |  |  |  |  |  |  |  |  |  |  |  |  |  |  |  |  |  |  |  |  |  |  |  |
| 1. 10 *T. disseminatum* (Chwalkowo Koscielne) | 0.27 | 0.28 | 0.32 | 0.27 | 0.29 | 0.31 | 0.32 | 0.34 | 0.27 | 0.28 | 0 |  |  |  |  |  |  |  |  |  |  |  |  |  |  |  |  |  |  |  |  |  |  |
| 1. 5 *T. dissimile* (Osowiec) | 0.33 | 0.33 | 0.37 | 0.32 | 0.32 | 0.30 | 0.32 | 0.34 | 0.31 | 0.29 | 0.24 | 0 |  |  |  |  |  |  |  |  |  |  |  |  |  |  |  |  |  |  |  |  |  |
| 1. 21 *T. jugiferum* (Blazowa) | 0.42 | 0.44 | 0.43 | 0.43 | 0.43 | 0.49 | 0.39 | 0.46 | 0.43 | 0.45 | 0.42 | 0.43 | 0 |  |  |  |  |  |  |  |  |  |  |  |  |  |  |  |  |  |  |  |  |
| 1. 11 *T. lacistophyllum* (Gdansk, Stogi) | 0.40 | 0.41 | 0.45 | 0.40 | 0.42 | 0.42 | 0.42 | 0.43 | 0.40 | 0.38 | 0.38 | 0.39 | 0.45 | 0 |  |  |  |  |  |  |  |  |  |  |  |  |  |  |  |  |  |  |  |
| 1. 27 *T. parnassicum* (Miedzianka) | 0.38 | 0.39 | 0.43 | 0.39 | 0.38 | 0.37 | 0.35 | 0.35 | 0.33 | 0.28 | 0.37 | 0.36 | 0.47 | 0.35 | 0 |  |  |  |  |  |  |  |  |  |  |  |  |  |  |  |  |  |  |
| 1. 30 *T. parnassicum* (Kusieta) | 0.37 | 0.37 | 0.41 | 0.38 | 0.37 | 0.36 | 0.33 | 0.34 | 0.32 | 0.28 | 0.36 | 0.37 | 0.48 | 0.34 | **0.03** | 0 |  |  |  |  |  |  |  |  |  |  |  |  |  |  |  |  |  |
| 1. 9 *T. parnassicum* (Kusieta) | 0.36 | 0.37 | 0.41 | 0.38 | 0.36 | 0.36 | 0.34 | 0.34 | 0.31 | 0.28 | 0.36 | 0.37 | 0.47 | 0.36 | 0.05 | **0.03** | 0 |  |  |  |  |  |  |  |  |  |  |  |  |  |  |  |  |
| 1. 14 *T. plumbeum* (Dzwirzyno) | 0.32 | 0.32 | 0.35 | 0.32 | 0.35 | 0.37 | 0.31 | 0.33 | 0.29 | 0.31 | 0.33 | 0.35 | 0.46 | 0.42 | 0.32 | 0.31 | 0.30 | 0 |  |  |  |  |  |  |  |  |  |  |  |  |  |  |  |
| 1. 48 *T. plumbeum* (between Keblowo and Swietno) | 0.30 | 0.30 | 0.29 | 0.29 | 0.33 | 0.35 | 0.30 | 0.32 | 0.25 | 0.28 | 0.33 | 0.35 | 0.41 | 0.42 | 0.32 | 0.33 | 0.32 | 0.25 | 0 |  |  |  |  |  |  |  |  |  |  |  |  |  |  |
| 1. 49 *T. plumbeum* (Sasieczno) | 0.31 | 0.32 | 0.30 | 0.31 | 0.32 | 0.34 | 0.34 | 0.35 | 0.27 | 0.31 | 0.34 | 0.34 | 0.45 | 0.42 | 0.34 | 0.34 | 0.34 | 0.28 | 0.09 | 0 |  |  |  |  |  |  |  |  |  |  |  |  |  |
| 1. 50 *T. plumbeum (near Golub-Dobrzyn)* | 0.30 | 0.31 | 0.35 | 0.30 | 0.32 | 0.35 | 0.29 | 0.36 | 0.27 | 0.28 | 0.31 | 0.32 | 0.42 | 0.39 | 0.27 | 0.28 | 0.28 | 0.21 | 0.19 | 0.22 | 0 |  |  |  |  |  |  |  |  |  |  |  |  |
| 1. 38 *T. cf. plumbeum* (Wola Mala) | 0.32 | 0.36 | 0.34 | 0.34 | 0.33 | 0.32 | 0.35 | 0.34 | 0.27 | 0.30 | 0.24 | 0.34 | 0.48 | 0.39 | 0.32 | 0.31 | 0.33 | 0.26 | 0.27 | 0.25 | 0.27 | 0 |  |  |  |  |  |  |  |  |  |  |  |
| 1. 33 *T. sandomiriense* (Kamien Lukawski) | 0.39 | 0.41 | 0.39 | 0.41 | 0.36 | 0.35 | 0.41 | 0.38 | 0.33 | 0.34 | 0.37 | 0.40 | **0.56** | 0.48 | 0.37 | 0.36 | 0.38 | 0.33 | 0.35 | 0.34 | 0.34 | 0.27 | 0 |  |  |  |  |  |  |  |  |  |  |
| 1. 36 *T. scanicum* (Lysakow Kolonia) | 0.31 | 0.32 | 0.33 | 0.33 | 0.37 | 0.34 | 0.38 | 0.37 | 0.32 | 0.36 | 0.37 | 0.35 | 0.47 | 0.43 | 0.38 | 0.37 | 0.36 | 0.27 | 0.33 | 0.31 | 0.28 | 0.30 | 0.33 | 0 |  |  |  |  |  |  |  |  |  |
| 1. 44 *T. scanicum* (Pila) | 0.33 | 0.33 | 0.32 | 0.31 | 0.39 | 0.39 | 0.37 | 0.39 | 0.32 | 0.34 | 0.34 | 0.39 | 0.47 | 0.47 | 0.43 | 0.40 | 0.41 | 0.34 | 0.29 | 0.29 | 0.31 | 0.33 | 0.39 | 0.28 | 0 |  |  |  |  |  |  |  |  |
| 1. 45 *T. scanicum* (Mlodzieszyn) | 0.30 | 0.28 | 0.32 | 0.30 | 0.35 | 0.34 | 0.36 | 0.35 | 0.28 | 0.30 | 0.35 | 0.34 | 0.46 | 0.42 | 0.32 | 0.31 | 0.30 | 0.22 | 0.28 | 0.27 | 0.23 | 0.30 | 0.35 | 0.15 | 0.23 | 0 |  |  |  |  |  |  |  |
| 1. 46 *T. scanicum* (Sowia Gora) | 0.33 | 0.34 | 0.34 | 0.35 | 0.35 | 0.35 | 0.38 | 0.34 | 0.30 | 0.31 | 0.35 | 0.36 | 0.45 | 0.43 | 0.35 | 0.34 | 0.33 | 0.28 | 0.27 | 0.27 | 0.23 | 0.29 | 0.36 | 0.20 | 0.21 | 0.12 | 0 |  |  |  |  |  |  |
| 1. 6 *T. scanicum* (Zbrzeznica) | 0.31 | 0.32 | 0.33 | 0.32 | 0.35 | 0.34 | 0.37 | 0.36 | 0.30 | 0.31 | 0.35 | 0.35 | 0.46 | 0.42 | 0.32 | 0.31 | 0.31 | 0.25 | 0.28 | 0.28 | 0.24 | 0.28 | 0.34 | 0.17 | 0.22 | 0.07 | 0.09 | 0 |  |  |  |  |  |
| 1. 20 *T. stridulum* (Blazowa) | 0.37 | 0.36 | 0.39 | 0.39 | 0.41 | 0.41 | 0.36 | 0.45 | 0.35 | 0.39 | 0.37 | 0.36 | 0.39 | 0.39 | 0.40 | 0.39 | 0.37 | 0.36 | 0.35 | 0.36 | 0.31 | 0.39 | 0.42 | 0.37 | 0.34 | 0.32 | 0.33 | 0.33 | 0 |  |  |  |  |
| 1. 4 *T. tenuilobum* (Gora Miedzianka) | 0.41 | 0.40 | 0.43 | 0.42 | 0.42 | 0.42 | 0.41 | 0.41 | 0.37 | 0.36 | 0.42 | 0.44 | 0.52 | 0.42 | 0.35 | 0.34 | 0.34 | 0.42 | 0.38 | 0.42 | 0.39 | 0.44 | 0.45 | 0.39 | 0.43 | 0.34 | 0.37 | 0.36 | 0.40 | 0 |  |  |  |
| 1. 53 *T. tortilobum* (Gdansk Stogi) | 0.34 | 0.35 | 0.36 | 0.32 | 0.34 | 0.37 | 0.40 | 0.38 | 0.33 | 0.35 | 0.39 | 0.38 | 0.49 | 0.37 | 0.34 | 0.34 | 0.34 | 0.36 | 0.33 | 0.32 | 0.31 | 0.36 | 0.40 | 0.37 | 0.36 | 0.33 | 0.36 | 0.34 | 0.38 | 0.41 | 0 |  |  |
| 1. 54 *T. tortilobum* (Gdansk Stogi 2) | 0.37 | 0.36 | 0.37 | 0.34 | 0.34 | 0.38 | 0.43 | 0.39 | 0.35 | 0.36 | 0.42 | 0.40 | 0.50 | 0.36 | 0.36 | 0.36 | 0.36 | 0.36 | 0.35 | 0.34 | 0.33 | 0.37 | 0.42 | 0.38 | 0.37 | 0.34 | 0.36 | 0.35 | 0.40 | 0.42 | 0.04 | 0 |  |
| 1. 55 *T. tortilobum* (Gdansk Park Rolanda) | 0.36 | 0.36 | 0.37 | 0.34 | 0.34 | 0.37 | 0.42 | 0.38 | 0.35 | 0.36 | 0.41 | 0.39 | 0.51 | 0.36 | 0.34 | 0.34 | 0.33 | 0.38 | 0.35 | 0.34 | 0.33 | 0.37 | 0.42 | 0.38 | 0.37 | 0.34 | 0.36 | 0.35 | 0.40 | 0.42 | **0.03** | 0.04 | 0 |

Table S1 Genetic distance between *Taraxacum* genotypes.
